# Differentiating salmonid migratory ecotypes through stable isotope analysis of collagen: Archaeological and ecological applications

**DOI:** 10.1371/journal.pone.0232180

**Published:** 2020-04-28

**Authors:** Eric Guiry, Thomas C. A. Royle, R. G. Matson, Hillary Ward, Tyler Weir, Nicholas Waber, Thomas J. Brown, Brian P. V. Hunt, Michael H. H. Price, Bruce P. Finney, Masahide Kaeriyama, Yuxue Qin, Dongya Y. Yang, Paul Szpak

**Affiliations:** 1 Department of Anthropology, Trent University, Peterborough, Ontario, Canada; 2 School of Archaeology and Ancient History, University of Leicester, Leicester, United Kingdom; 3 Department of Anthropology, University of British Columbia, Vancouver, British Columbia, Canada; 4 Department of Archaeology, Ancient DNA Laboratory, Simon Fraser University, Burnaby, British Columbia, Canada; 5 Ministry of Forests, Lands, Natural Resource Operations and Rural Development, Government of British Columbia, Penticton, British Columbia, Canada; 6 Institute for the Oceans and Fisheries, University of British Columbia, Vancouver, British Columbia, Canada; 7 Department of Earth, Ocean and Atmospheric Sciences, University of British Columbia, Vancouver, British Columbia, Canada; 8 Hakai Institute, Heriot Bay, British Columbia, Canada; 9 Department of Biological Sciences, Earth to Ocean Research Group, Simon Fraser University, British Columbia, Canada; 10 Department of Biological Sciences, Idaho State University, Pocatello, Idaho, United States of America; 11 Department of Geosciences, Idaho State University, Pocatello, Idaho, United States of America; 12 Arctic Research Center, Hokkaido University, Sapporo, Hokkaido, Japan; 13 School of Marine Science and Environmental Engineering, Dalian Ocean University, Dalian, Liaoning, China; Senckenberg Gesellschaft fur Naturforschung, GERMANY

## Abstract

The ability to distinguish between different migratory behaviours (e.g., anadromy and potamodromy) in fish can provide important insights into the ecology, evolution, and conservation of many aquatic species. We present a simple stable carbon isotope (*δ*^13^C) approach for distinguishing between sockeye (anadromous ocean migrants) and kokanee (potamodromous freshwater residents), two migratory ecotypes of *Oncorhynchus nerka* (Salmonidae) that is applicable throughout most of their range across coastal regions of the North Pacific Ocean. Analyses of kokanee (*n* = 239) and sockeye (*n* = 417) from 87 sites spanning the North Pacific (Russia to California) show that anadromous and potamodromous ecotypes are broadly distinguishable on the basis of the *δ*^13^C values of their scale and bone collagen. We present three case studies demonstrating how this approach can address questions in archaeology, archival, and conservation research. Relative to conventional methods for determining migratory status, which typically apply chemical analyses to otoliths or involve genetic analyses of tissues, the δ^13^C approach outlined here has the benefit of being non-lethal (when applied to scales), cost-effective, widely available commercially, and should be much more broadly accessible for addressing archaeological questions since the recovery of otoliths at archaeological sites is rare.

## Introduction

Shifting migratory modes are an important behavioural and evolutionary characteristic of many economically and environmentally important fish species [1–3]. The flexibility to switch between life history strategies, such as migrating to the ocean and back (anadromy) [4], on the one hand, and living solely in freshwater (potamodromy) [5], on the other hand, can contribute to species’ adaptability to new environments and resilience under difficult and changing conditions [2, 6]. For this reason, there has been long standing, multidisciplinary interest in developing independent techniques for distinguishing between anadromous and potamodromous ecotypes [e.g., 7–9]. For instance, the ability to independently assess ecotype can allow for more detailed studies of how sympatric populations respond differently to changing environmental conditions through time. This may, in turn, help to guide future conservation policy and enhance environmental restoration efforts. Techniques for differentiating ecotypes could also be applied to specimens in natural history museum collections to validate key aspects of archival metadata or to study the historical ecology of endangered or extinct populations. Differentiating these ecotypes is important not only for biological and conservation research but also for archaeologists interested in understanding past fishing practices [e.g., 10–12].

Isotopic and elemental analyses have been at the forefront of efforts to distinguish between anadromous and potamodromous ecotypes. This is because there can be large differences between the isotopic and elemental composition of typical freshwater and marine environments that are passed along the food web to consumers, like fish, providing a useful marker for anadromous and potamodromous behaviours. Among these, the most commonly used approaches have been measuring the Sr/Ca and Sr/Ba ratios [8, 13] as well as the isotopic composition of radiogenic strontium (^87^Sr/^86^Sr) [14, 15], stable sulfur isotopes (δ^34^S) [7, 16], and stable oxygen isotopes (δ^18^O) [17] in fish tissues. These approaches, which are typically applied to fish otoliths, have been deployed by ecologists [8], palaeontologists [17, 18], and archaeologists [19, 20] for decades to establish whether fish capable of anadromy have migrated to the ocean and back as part of their life history or if they had remained in a freshwater environment their entire lives.

Despite their popularity and highly successful track record, there are several challenges associated with the application of these conventional approaches, the magnitude of which will depend on the research field. From an archaeological perspective, fish otoliths, in general, are not frequently recovered [21] due to their small size. Although researchers have successfully applied otolith-based techniques to archaeological specimens [e.g., 22, 23], their use will be circumscribed to the limited number of archaeological sites where preservation conditions and excavation practices have allowed for the recovery of fish otoliths in larger quantities [21]. Moreover, applying strontium-based analyses (either isotopic composition or elemental concentration) to bones, which are typically the most abundant fish remains recovered archaeologically, as an alternative to otoliths, is potentially complicated by the greater susceptibility of fish bone to diagenetic alterations (i.e., addition or loss of Sr and other elements in the burial environment [18, 24]). Tooth enamel, which is less porous than bone, and may be relatively more resistant to diagenetic contamination, has also been used [20]. However, not all fish species have suitable teeth and tooth bearing elements with intact teeth are often much less frequently recovered archeologically. From an ecological perspective using otoliths could also be problematic. Extracting otoliths is fatal and while otoliths in many cases can be collected after a spawning run, when some species of fish are already near the end of their lifecycle, many fish do not die after spawning. From a conservation perspective, research methods that minimize invasive procedures that kill fish have a wide range of other benefits [e.g., 25–27]. Finally, for ecologists and archaeologists, relatively high labour, financial, and other resource costs are associated with undertaking sectioning and elemental and/or isotopic analyses of otoliths, which may be prohibitive for smaller research or conservation budgets. While the analysis of otoliths provides a powerful and unparalleled tool for reconstructing fish life histories in intricate detail, a simpler approach for distinguishing between potamodromous and anadromous behavioural strategies may be sufficient to meet the needs of many fisheries management conservation programs or archaeological and ecological research questions.

**Fig 1:**
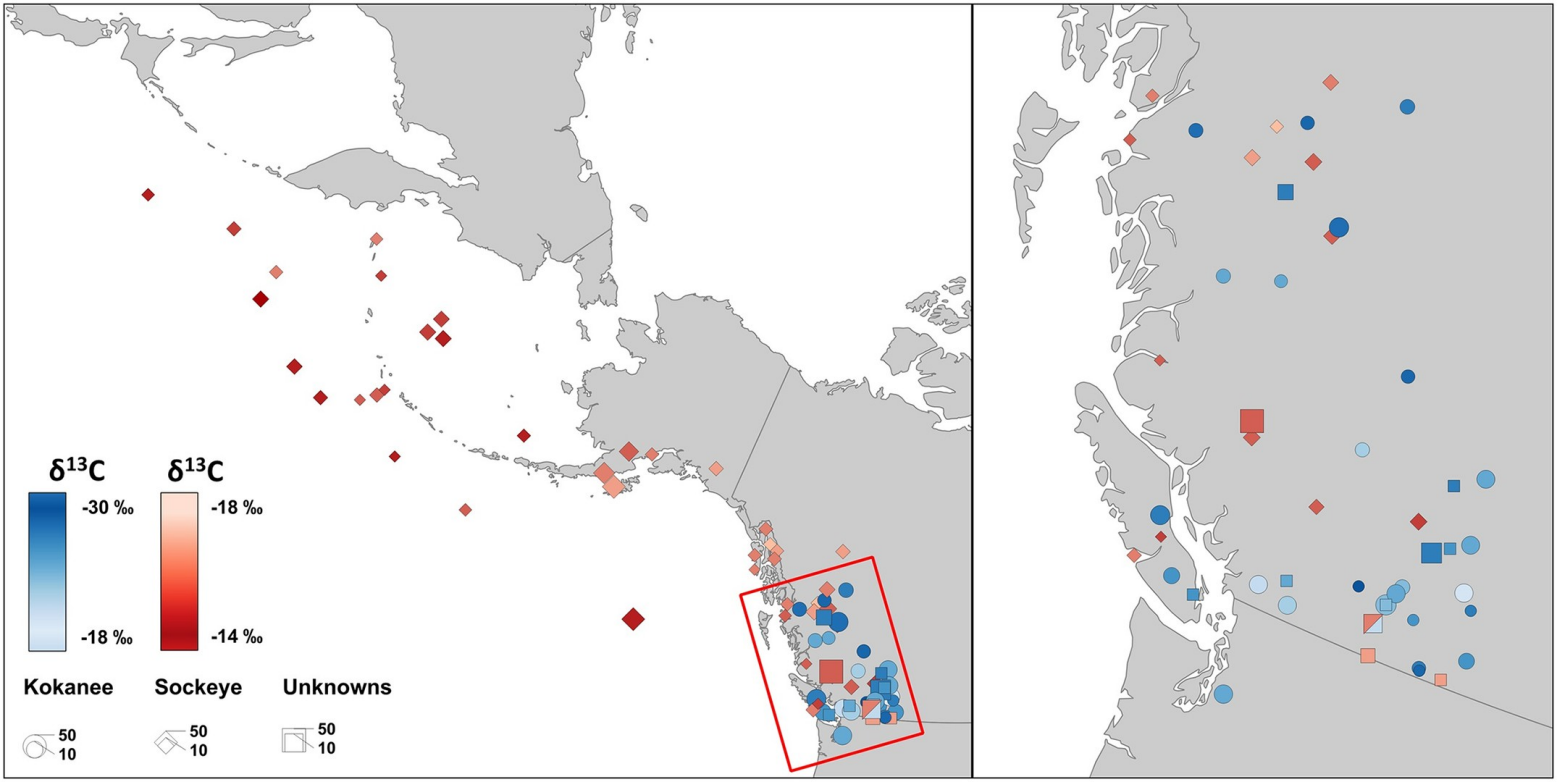
Map showing collection locations for samples. Known kokanee (circles) and sockeye (diamonds) as well as unknown (squares) samples are shaded in blue and red, respectively, according to the mean δ^13^C values.

Using stable carbon isotope (δ^13^C) compositions of collagen from 656 anadromous sockeye and potamodromous kokanee (both *Oncorhynchus nerka*, Salmonidae) from 87 sites across the northern Pacific Rim (Fig 1), we demonstrate a simple and cost-effective alternative approach for establishing the migratory life history of fish that are capable of anadromy. Because collagen can be extracted from both fish scales and bones, it offers an additional option for ecologists to sample fish non-lethally and to archaeologists for whom bone is often the only sample material available in great abundance. As collagen is also available in historical scale archives and preserved fish specimens curated at museums, this approach can also enable historical reconstructions of fish behaviours over the recent past. *O*. *nerka* provides an ideal test species to validate this approach because their pelagic planktivorous feeding behaviour mitigates potential issues with isotopic overlap between potamodromous and anadromous ecotypes (see Section, Stable carbon isotopes in aquatic environments). Moreover, throughout their wide geographic range, numerous freshwater resident *O*. *nerka* populations exist, allowing for a spatially comprehensive assessment of the effect of diverse environmental (differing carbon sources and productivity levels) and behavioural variability on kokanee stable carbon isotope compositions. To demonstrate the broader applicability of this approach we present three case studies in which stable carbon isotope analyses of *O*. *nerka* of unknown ecotype are used to distinguish between kokanee and sockeye at (1) archaeological sites, (2) in natural history collections, and (3) in modern conservation work. We also include stable nitrogen isotope (δ^15^N) compositions, which were measured in tandem with δ^13^C, to explore variation in freshwater and marine nitrogen inputs and cycling. In addition to stable isotope analyses, we also conducted ancient DNA (aDNA) analysis in order to confirm the species identity of a subset of the analyzed archaeological remains.

## Context

### Stable carbon isotopes in aquatic environments

Many previous studies have used δ^13^C of fish tissues as a basis for distinguishing between sympatric marine and freshwater ecotypes [e.g., 7, 28–30]. These studies have relied partly on the observation that food webs in marine environments can have elevated δ^13^C relative to their freshwater counterparts, which provides a useful marker for distinguishing between fish with anadromous and potamodromous life histories. All these applications, however, have been context-specific and often rely at least partly on additional evidence from other isotopic proxies, such as δ^34^S and δ^15^N measurements.

An important reason for why δ^13^C has not previously been deployed for use as a spatiotemporally broad-scale approach for distinguishing between freshwater and marine fish is that freshwater food webs can have an enormous range of isotopic variability [31]. This variability can create overlap between δ^13^C values for freshwater and marine conspecifics, making the utility of δ^13^C as a universal means of differentiating between potamodromous and anadromous ecotypes uncertain, particularly across broader geographical regions [31]. The stable carbon isotope composition of aquatic food webs is governed by a complex and highly interconnected set of processes, contributing to this variability (for reviews see [32–35]). While some environmental variables, such as temperature [36], may have similar impacts on δ^13^C of food webs in both marine and freshwater ecosystems, many factors have more potential to influence the isotopic composition of fish in freshwater environments due to their smaller size and constrained resource pools (for review see [31]). Despite these complexities, and potential for overlap between the stable carbon isotope compositions of fish inhabiting both marine and freshwater environments, it may still be possible to use δ^13^C (a simpler and more cost-effective alternative; see Section Introduction) to differentiate between marine and freshwater ecotypes within the same taxon provided the dietary behaviour of the species analyzed is sufficiently specialized (i.e., the species is not a dietary generalist with potential to feed across other major axes of systematic δ^13^C variations, such as nearshore and offshore areas).

### Stable nitrogen isotopes in aquatic environments

Although not the focus of this study, δ^15^N was obtained in tandem with δ^13^C measurements, and we provide these isotopic compositions here to give additional insights into freshwater and marine environments. Stable nitrogen isotope compositions undergo a stepwise increase by roughly 3 to 5‰ at each trophic step within a food web [37] and have therefore been used to explore predator-prey relationships and trophic dynamics [38, 39]. However, the δ^15^N of key nitrogenous nutrients that form the baseline of both marine and freshwater environments is controlled by a wide range of variables (e.g., microbial activity, productivity, oxygenation, alkalinity) that can result in large shifts in ecosystems’ stable nitrogen isotope baselines, even over relatively small spatiotemporal spans [31, 40, 41]. As with δ^13^C, this is particularly the case in freshwater ecosystems where, in comparison with larger marine environments, the smaller overall pool of dissolved inorganic nitrogen available in a given local nitrogen cycle, may be more sensitive to fluctuations in key processes governing isotopic fractionations [31]. In this context, and given that our analyses focus on adults from a single species of fish (mostly feeding at a similar trophic level) from a very wide range of environments, we expect variation in δ^15^N to primarily reflect regional differences in nitrogen inputs and cycling [42].

### Collagen and temporal resolution of diet

Biologically, collagen has excellent properties for assessing questions about animal life histories. Isotopic compositions of tissues can vary based on differing time scales that are specific to each tissue’s turnover rate [43]. For example, the muscle or organ tissues typically analyzed in ecological research will have isotopic compositions reflecting diet over a period of months or weeks [44,45]. In contrast, the slow turnover of collagen in bone, which remodels over the course of an organism’s lifetime, provides an average perspective on diet during the period over which a fish underwent the bulk of its growth [46, 47]. For this reason, the isotopic composition of bone collagen from shorter lived fish species should provide a lifetime average perspective on dietary intake (weighted towards the period when their growth-rate was fastest). From an isotopic and compositional perspective, scale collagen is directly comparable with bone collagen [48] and is also similar in that it has an isotopic composition reflecting a long-term average of diet because it is incrementally laid down during growth [49, 50]. For migratory fish like salmon, research comparing δ^13^C in scales from smolts (juveniles) departing their natal streams, and adults, returning to those same streams (after only one winter at sea), has shown that the diets of adults undergoing substantial growth quickly ‘overwrites’ the isotopic signal from their natal freshwater environments [51]. Therefore, in addition to being a readily available and potentially non-lethal source of samples, scale and bone collagen provides an ideal material for testing hypotheses about potamodromous *versus* anadromous life histories in fish because it gives a time-averaged perspective [52] that is less susceptible to short-term fluctuations resulting from seasonal change or occasional behavioural aberrations.

### Species identification of archaeological Pacific salmonid remains

Throughout the northern Pacific Rim, Pacific salmonid (*Oncorhynchus* spp.) bones are commonly recovered from archaeological sites, often in large quantities [53–55]. At most sites in the region, most archaeologically recovered Pacific salmonid bones are vertebrae due to their high bone density relative to cranial and other post-cranial elements mitigating destructive taphonomic processes [56]. With the exception of a few cranial elements, most Pacific salmonid skeletal elements, including vertebrae, lack inter-specific morphological variation meaning it is not possible to assign species-level identifications to most archaeological Pacific salmonid remains through conventional comparative morphological approaches [57, 58]. Consequently, metric [59], morphometric [60], radiographic [61], and stable isotope [62, 63] methods have been developed for the taxonomic identification of archaeological Pacific salmonid vertebrae. However, these methods often yield inaccurate results or are unable to refine identifications to a particular species rather than groups of species [62–67]. Due to the limitations of other species identification approaches, both aDNA analysis [e.g., 12, 58, 59, 64–66, 68–70] and peptide mass fingerprinting [e.g., 71], more commonly known as zooarchaeology by mass spectrometry (ZooMS) [72] are increasingly being used to assign species identities to archaeological Pacific salmonid remains. Both of these approaches can assign reliable species-level identifications to Pacific salmonid remains, making them useful for taxonomic identification. For these reasons, we used aDNA analysis to confirm the species identities of our archaeological samples (see Section Ancient DNA analysis).

## Methods

### Sampling

**Fig 2:**
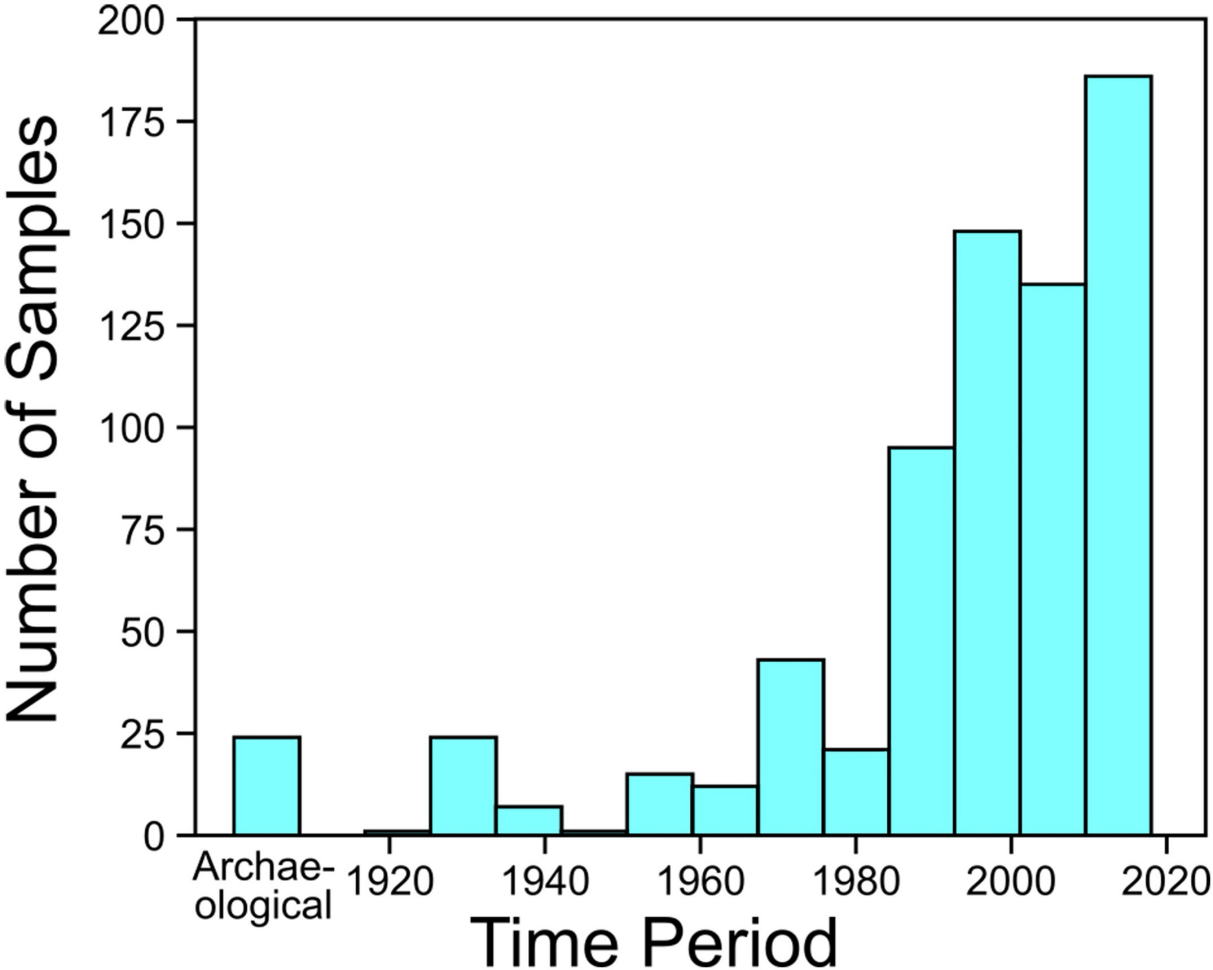
Number of samples versus collection date.

Samples included in this study are summarized in Table 5 in S1 Text. We collected scale samples from 213 potamodromous kokanee and 43 anadromous sockeye (hereafter referred to as kokanee and sockeye, respectively) from museums and biologists for isotopic analysis, with a view to maximizing geographical coverage for our δ^13^C baseline. Fish were assigned to either sockeye, kokanee, or unknown groups using information provided in associated records (i.e., identifications made by the biologists collecting or cataloguing specimens) or collection location (i.e., a kokanee identification could be assigned to fish collected in watersheds not draining into the Pacific Ocean or lacking anadromous sockeye). Temporally, these samples span the last century (Fig 2), with all decades since the 1920s represented but more prominent representation from the last three decades. Where possible, we also sourced δ^13^C data for muscle (only lipid corrected δ^13^C used), scale, and bone from the literature [73–82], including 380 sockeye and 17 kokanee. For our three case studies we worked with museums, archaeologists, First Nations, and conservation biologists to source samples. In all cases, samples (*n* = 58) came from specimens with no associated information about ecotype (i.e., whether they are potamodromous or anadromous). No live fish were sampled in this study. All materials from modern specimens were sourced from fish collected for previous studies, donated or purchased from licensed commercial fishers, or collected as part of government conservation programs. No permits were required for the described study, which complied with all relevant regulations. Complete repository information for all samples is provided in Table 5 in S1 Text and includes the following: the Royal British Columbia Museum (Victoria, BC, Canada), the Beaty Biodiversity Museum (Vancouver, BC Canada), the Royal Ontario Museum (Toronto, ON, Canada), the British Columbia Ministry of Forests, Lands and Natural Resource Operations (Penticton, BC, Canada), and the Xeni Gwet’in First Nation (curated at the Department of Anthropology, University of British Columbia, Vancouver, BC, Canada).

### Sample pre-treatment

In many cases, scales came from specimens curated in natural history collections that have been in long-term storage and treated with preservatives, typically formalin and ethanol. Therefore, scale collagen pre-treatments sought to maximize collagen purity and the removal of potential contaminants. Following Guiry and colleagues [83], scales were cleaned prior to isotopic analyses with a scalpel and sonicated in deionized water for 3 × 15 min to remove potential adhering contaminant materials (tissue, guanine). Cleaned scales were soaked in 1.0 M HCl for 2 min followed by additional rinses in an ultrasonic bath of deionized water 2 × 3–5 min. This acid pre-treatment removes the mineralized external plate from scales, leaving behind the underlying acid insoluble, collagen-rich fibrillar plate. This process should also help loosen any materials that may have settled on or become attached to the external surface of scale samples during their long-term storage. Scales were then air dried.

Bone collagen from modern and archaeological samples was extracted following established methods [84]. Bones surfaces were cleaned of debris and cut into small, ⁓3x3 mm chunks. Samples were then treated with a 2:1 chloroform-methanol solution in an ultrasonic bath (5–10 min each) to remove lipids [80, 85]. Following lipid removal, samples were demineralized by soaking in 0.5 M HCl. Samples were then rinsed to neutrality in deionized water. For archaeological samples, which may have become contaminated with humic acids and other base-soluble contaminants from their burial environment, an additional treatment was applied. These samples were soaked in 0.1 M NaOH multiple times in an ultrasonic bath, with the solution refreshed every 15 min until the solution remained clear, and then rinsed again in deionized water to neutrality. All samples were then solubilized in a 10^−3^ M HCl (pH ~3) solution in a heating oven (at 70 °C) for 48 h. Samples were then centrifuged and the solution transferred into a new tube, frozen, and lyophilized.

### Isotopic analyses

Scale and bone collagen stable carbon and nitrogen isotope analyses were performed on 0.5 mg subsamples using an elemental analyser (EA) coupled via continuous flow to an isotope ratio mass spectrometer (CF-IRMS) at the Department of Anthropology at The University of British Columbia (UBC; Vancouver, BC, Canada; *n* = 266) and at the Water Quality Research Centre at Trent University (TU; Peterborough, ON, Canada; *n* = 48). Duplicate or triplicate analyses were performed on 17% of samples (S1 Text). Collagen samples were combusted in tin capsules in a Vario MICRO cube EA coupled to an Isoprime IRMS (Elementar, Hanover, Germany) at UBC and an EA 300 (Eurovector, Pavia, Italy) coupled to a Horizon IRMS (Nu Instruments, Wrexham, UK) at TU. Carbon and nitrogen isotopic compositions were calibrated relative to VPDB and AIR, respectively, using USGS-40 and USGS-41 or USGS-41a [86, 87]. Instrumental accuracy and precision were monitored using internal collagen standards (S1 Text). Following Szpak and colleagues [88], analytical uncertainty was calculated to be ±0.13‰ and ±0.21‰ for *δ*^13^C and *δ*^15^N, respectively (S1 Text).

### Collagen quality

There has been some suggestion in the literature that fish bone collagen is particularly susceptible to diagenesis [89] and it may therefore be useful to outline factors relevant to assessing collagen quality (QC) for specimens of different ages. Because the amino acid composition of collagen is well understood [89], the percent elemental concentrations (%C and %N) and carbon-to-nitrogen ratio (C:N_atomic_) of collagen provides a robust proxy for the presence of exogenous carbon and nitrogen, which could skew isotopic measurements. These indicators can therefore be used to screen for collagen diagenesis and assess overall collagen preservation.

It is worth highlighting that, while archaeological fish bone collagen is often found to have higher C:N_atomic_ than bone from other taxa, we can still expect that the isotopic compositions of archaeological and historical specimens will be directly comparable with data from modern specimens, provided they have C:N_atomic_ falling within the acceptable range [89]. Owing to their higher proline and hydroxyproline content, fish collagens have a lower melting point and less stability relative to those of mammals [90] and may therefore be more prone to the effects of leaching and selective amino acid loss [89]. However, because this kind of diagenetic alteration would leave collagen enriched in amino acids like glycine and alanine, which have lower C:N_atomic_ [91], diagenetic processes such as selective amino acid loss are not likely to be behind the trend in higher C:N_atomic_ observed in archaeological fish. Instead, factors contributing to higher C:N_atomic_ likely involve the greater susceptibility of fish bone (being porous, with loosely packed collagen fibrils) to exogenous contaminants [89], a factor that would also apply to historical specimen fixed with formalin.

To assess collagen quality, we used established ranges for carbon (>13.8%) and nitrogen (>4.0%) as well as C:N_atomic_ (2.9–3.6) [92, 93]. These collagen quality indicators are routinely used to screen for collagen degradation and diagenesis in archaeological studies and can also serve as an indicator for issues related to residual museum preservatives (i.e., formalin).

### Carbon isotope corrections

Comparing the stable carbon isotope compositions of *O*. *nerka* from a range of time-periods as well as with varying curatorial histories requires additional isotopic corrections.

#### Suess effect correction

Globally, the δ^13^C of CO_2_, which supplies dissolved inorganic carbon (DIC) for primary production in both freshwater and marine environments, has been declining since industrial processes began releasing additional ^13^C-depleted carbon into the atmosphere through fossil fuel combustion [94]. To compare data from different time periods, we calculated the correction for the Suess effect [94] on δ^13^C values for tissues from modern and historical samples (Δ^13^C_Suess_) as outlined below. Because the residence time for DIC differs between water bodies based on several factors, different Δ^13^C_Suess_ are applied for tissues from freshwater and marine organisms [95].

For δ^13^C from anadromous fish, we applied a correction for the Suess effect following Hilton and colleagues [96, 97] as follows:

$$\Delta^{13}C_{Suess}=a_{waterbody}*e^{\left(yrs•b\right)}$$

where “*a*”is the annual rate of decrease in δ^13^C for the water body, “*yrs*” is the number of years since 1850 CE, and “*b*” is the shape of the exponential curve (0.027) defined by the decrease in oceanic δ^13^C observed by Gruber and colleagues [98] between 1945 and 1997 CE. Sockeye analyzed as part of this study span a wide geographical area across the North Pacific Ocean. Reflecting this broad distribution, and following others working in this region [e.g., 97, 99], we use Quay and colleagues’ [100] estimate of –0.014 for the annual rate of decrease in δ^13^C in the North Pacific Ocean.

Following others [e.g., 101] working in freshwater environments, for δ^13^C from potamodromous fish, we applied a correction for the Suess effect following Verberg [102] as follows:

$$\Delta^{13}C_{Suess}=({7.7738118e}^{-16}\times {yrs}^{6})-({1.2222044e}^{-11}\times {yrs}^{5})+\;({7.1612441e}^{-8}\times {yrs}^{4})-({2.1017147e}^{-4}\times {yrs}^{3})+({3.3316112e}^{-1}\times {yrs}^{2})-(273.715025\times yrs)+91703.261$$

where “*yrs*” is the number of years since 1850 CE. It is important to note that, unlike the correction used for anadromous fish, the equation used for potamodromous fish does not account for temporal offsets related to the complexities of freshwater aquatic carbon cycling. An increasing number of _14_C studies highlight the wide range of variation that can occur in the age and reservoir period for DIC used by primary producers at the base of freshwater environments, which can extend to hundreds and even thousands of years [e.g., 103–110]. This offset means that there may be considerable temporal lag between the impact of the Suess effect on the isotopic composition of atmospheric and freshwater DIC pools. Unfortunately, data are not available to calculate the offsets for each freshwater system in this study. It is therefore likely that the correction applied for the Suess effect on freshwater fish overestimates Δ^13^C_Suess_, which would serve to decrease the magnitude of potential difference between anadromous and potamodromous *O*. *nerka*.

#### Muscle correction

Several studies have observed a systematic offset [e.g., 48, 81, 111–113] between fish scale collagen and muscle δ^13^C, which has previously been characterized for Pacific salmonid species (⁓ +3.69 ‰) [81]. This correction has been applied to data for muscle that we have included in our baseline from the literature.

#### Formalin fixation correction

Samples from museum archived specimens have often been preserved using formalin and ethanol. Typically, this is achieved via a multiday soaking in a 10% formalin solution followed by long-term storage in ethanol. This process is known to introduce carbon from formalin to the preserved tissues [114, 115]. Numerous studies have investigated the effects of formalin fixation on fish muscle tissues [e.g., 116–121] and typically show offsets for δ^13^C (⁓0 to –2 ‰) and δ^15^N (⁓0 to +1‰). To date, we are unaware of any studies that have investigated the impact of formalin fixation or other preservation techniques on bone or scale collagen. However, based on recent experimental results from analyses of salmonid tissues (*Salmo salar* and *Oncorhynchus tshawytscha*) formalin fixation appears to have little effect on the δ^13^C (–0.4‰) and δ^15^N (0.0‰) composition of bone and scale collagen. In this context, we have used a conservative δ^13^C correction of +0.5‰ for museums specimens that have been formalin fixed. In comparison with the range for δ^13^C that we are considering in this study (>25‰), these effects should be inconsequential for distinguishing between ecotypes. Moreover, any specimens with excessive carbon contamination resulting from formalin fixation should be identifiable based on an elevated C:N_atomic_ ratio and excluded from the study (see Section Collagen quality).

#### Statistical analyses

Statistical comparisons of sockeye and kokanee stable isotope compositions were performed using PAST version 3.22 [122]. To establish whether δ^13^C and δ^15^N differed significantly between our baseline sockeye and kokanee groups, we used Mann-Whitney U tests with Bonferroni corrected *p* values. The Shapiro-Wilk’s test showed that δ^13^C and δ^15^N values for both sockeye (*p* <0.005) and kokanee (*p* <0.003) were not normally distributed. Results of the Mann-Whitney U test showed that sockeye and kokanee differed significantly from one another in both δ^13^C and δ^15^N values (*p* <0.0001).

In order to assess whether individual specimens of unknown ecotype could be assigned to either sockeye or kokanee ecotypes according to their δ^13^C values, we used the ‘single-case’ test, which can test whether a single value comes from the same population as a given sample. With this test we evaluated whether values from individual specimens in the unknown group were statistically more similar to values in sockeye or kokanee baseline groups using a modified *t*-test. While this test is imperfect because it assumes that the individual value given is an estimate, with variance and standard deviation similar to the sample with which it is being compared, it is a useful way to assess relationships between individual and sample values [123, 124].

### Ancient DNA analysis

Our isotopic analysis included 25 archaeological salmonid vertebrae from two archaeological sites located within the Interior Plateau of British Columbia, Canada, in Xeni Gwet’in territory dating to between ca. 2400 and 50 years before present [125]. This sample consisted of 8 vertebrae from the Plateau Pithouse Tradition-associated Shields site (EkSa-13), and 17 vertebrae from the ancestral Tsilhqot’in Bear Lake site (EkSa-36) (Fig 1). These vertebrae were preliminarily identified as *O*. *nerka* (*n* = 24) or *Oncorhynchus* sp. (*n* = 1; much larger) based on their size. However, as speciating Pacific salmonid vertebrae through non-biomolecular methods is difficult (See Section Species identification of archaeological Pacific salmonid remains), we used aDNA analysis to confirm species identifications and obtain sex identifications [70, 126] for a subset of the archaeological vertebrae (*n* = 10). Detailed descriptions of the methods employed in the aDNA analysis are provided in the supplementary materials (S2 Text).

All pre-PCR procedures (decontamination, DNA extraction, and PCR setup) were conducted at the Department of Archaeology at Simon Fraser University (Burnaby, BC, Canada), in a dedicated aDNA laboratory and followed strict contamination control protocols [127]. All of the analyzed samples were decontaminated prior to DNA extraction using established protocols [128]. DNA was extracted from the decontaminated samples using a silica-spin column method [129] as modified by Yang and colleagues [130]. Sex identities were assigned to the samples using two PCR sex identification assays (termed *clock1a*/*sdY* and D-loop/*sdY)* designed by Royle and colleagues [126]. Following Royle and colleagues [126], we sought to assign species identifications to the remains by sequencing a 249 bp fragment of the mitochondrial D-loop co-amplified as an internal positive control in one of the sex identification assays (D-loop/*sdY* assay). To confirm the species identifications assigned to the samples, we also sequenced a 168 bp fragment of *cytochrome b* (*cytb*), which was amplified in a singleplex PCR [70, 131]. D-loop was also amplified from a single sample (IUBC 5226) through a singleplex PCR in order to improve sequencing quality. To monitor for contamination, blank extraction controls were processed alongside the samples and negative PCR controls were included in each PCR run.

The obtained sequences were visually edited, truncated to remove the primer sequences, and compiled in ChromasPro v 2.1.8 (http://technelysium.com.au). Following Yang and colleagues [70], the obtained sequences were compared with Pacific salmonid reference sequences through BLASTn searches [132] against GenBank [133], and neighbour-joining trees constructed in MEGA X [134]. Species-level identifications were assigned to samples if the obtained *cytb* and D-loop sequences matched or closely resembled sequences from a single species and differed from closely related species [70].

## Results

### Isotopic analyses

**Fig 3:**
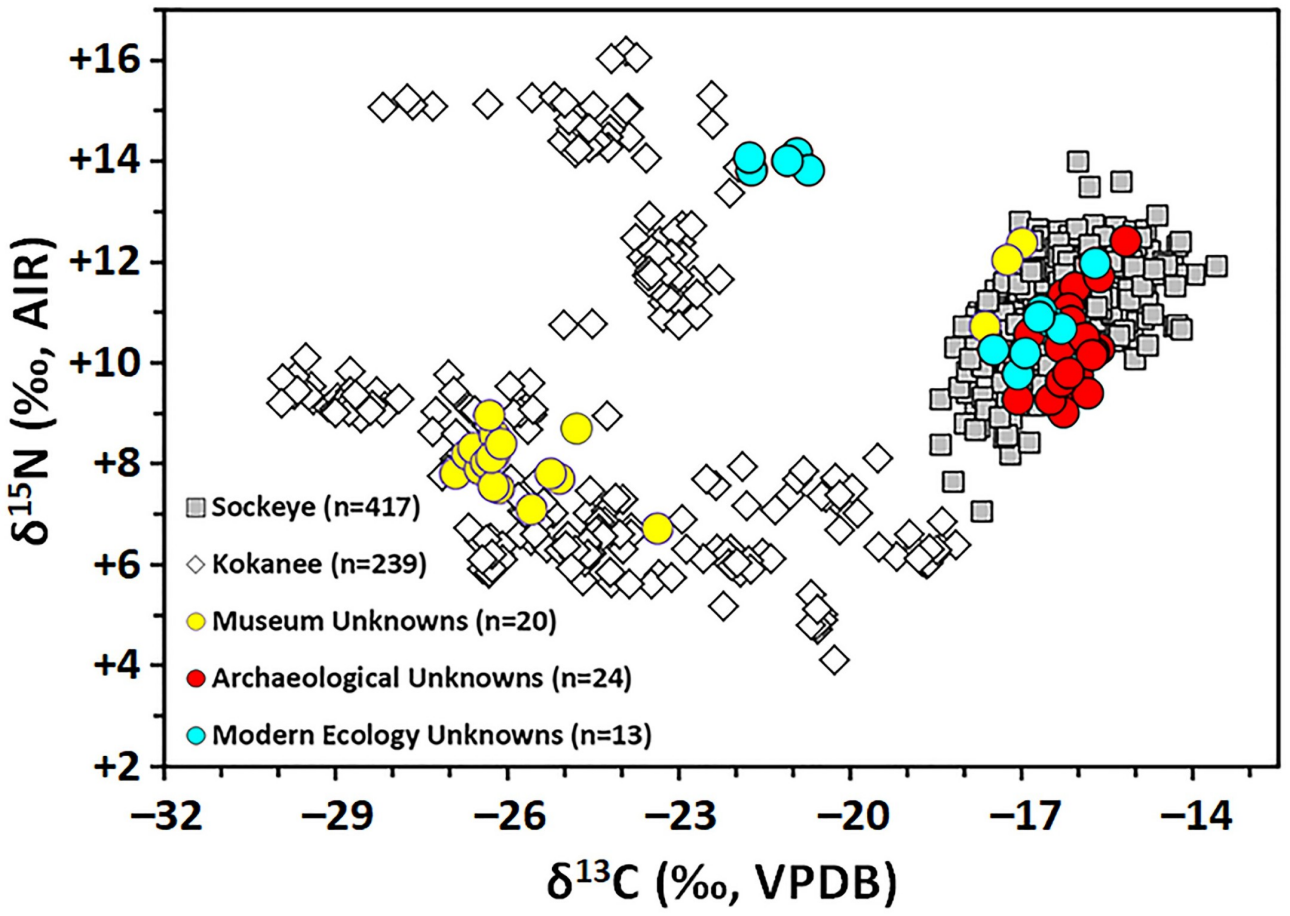
Stable carbon and nitrogen isotope compositions of *O*. *nerka* samples.

The results of the isotopic analyses are presented in Fig 3 and Table 5 in S1 Text. We analyzed bone or scale collagen samples from 342 specimens, 92% (*n* = 314) of which produced acceptable collagen quality criteria. Our baseline, including data sourced from the literature, is comprised of 239 kokanee and 417 sockeye collected at 87 sampling localities (Fig 1). These sampling localities included sites in the North Pacific Ocean as well as lakes and rivers in Canada, Russia, and the United States of America (Fig 1). As expected based on their behavioural ecology and use of freshwater environments, kokanee (δ^13^C range = 11.8‰, between –29.9 and –18.1‰, mean = –24.3‰; δ^15^N range = 12.1‰, between +4.1 and +16.2‰, mean = +9.0‰) have a much wider range of isotopic compositions relative to sockeye (δ^13^C range = 4.8‰, between –18.4 and –13.6 ‰, mean = –16.3‰; δ^15^N range = 6.9‰, between +7.1 and +14.0‰, mean = +11.0‰). While the food webs that kokanee inhabit are subject to a highly diverse set of carbon sources and carbon cycling processes (for review see [31]), those of marine sockeye δ^13^C appear to be much less variable.

Notably, the ranges for δ^13^C of sockeye and kokanee collagen overlap by only 0.3‰. Kokanee samples with δ^13^C values falling within the range observed for sockeye (i.e., > –18.4‰) are all derived from a population that inhabited a single system, the Alouette Reservoir, British Columbia, Canada (see Table 5 in S1 Text for details), in which ^13^C-enriched carbon sources or environmental variables have contributed to high δ^13^C baseline values. The Alouette Reservoir ecosystem is part of a nutrient addition program, aimed at supporting its kokanee population [135, 136], and the increased productivity resulting from the addition of agricultural fertilizers could be responsible for the higher δ^13^C values observed in kokanee from this location. It is important to note, however, that the freshwater Suess effect corrections (see Section Suess effect correction) applied to this population (+2.1‰; for comparison, marine Suess correction is only +1.1‰ for the same year) are also large. In not accounting for the possibility of old carbon sources (i.e., from non-atmospheric DIC and reservoir offsets), δ^13^C for the Alouette Reservoir kokanee has potentially been overcorrected, thereby pushing them into the baseline range for sockeye. Nonetheless, the Alouette Reservoir specimens demonstrate that, while atypical, it is possible for kokanee and sockeye to have similar δ^13^C values.

Despite slight overlap between ecotypes, kokanee and sockeye δ^13^C values are significantly different (Mann Whitney U, *p* <0.0001) at a broad temporal and geographical scale, suggesting that these data can provide a robust baseline for distinguishing between these *O*. *nerka* ecotypes on the basis of collagen δ^13^C values. Using δ^13^C values, the single-case test found that all values from the unknown ecotype group that were equal to or lower than –19‰ were statistically similar to the kokanee baseline group, while values of –18‰ or greater were statistically similar to the sockeye baseline group. Therefore, the test confirmed visual inspection of the data suggesting that *O*. *nerka* with collagen δ^13^C values either side of –18 to –19‰ are broadly distinguishable as either the sockeye or kokanee ecotypes. This test also confirmed that collagen δ^13^C values between –18 to –19‰ cannot be reliably assigned to either ecotype.

Based on *O*. *nerka* behavioural ecology as well as the ways in which nitrogen is sourced and cycled through aquatic ecosystems [31], we had not anticipated that δ^15^N would provide a viable basis for distinguishing between freshwater and marine behavioural types. Due to a stepwise enrichment of 3–4‰ between trophic levels [37], δ^15^N is typically associated with trophic position in ecological and archaeological studies [38, 39]. Adult size for kokanee can range widely between populations and size variation could offer a possible partial explanation for the wide range observed here in kokanee δ^15^N, with larger individuals feeding at a higher trophic level. However, the δ^15^N range observed in kokanee, which would be consistent with a span of at least two to three trophic levels, is not consistent with what is known about the dietary behaviour of this species, which are typically planktivores (although sockeye have also been noted to sometimes eat small fish and squid [137]). Instead, a more parsimonious explanation would be relative differences in baseline δ^15^N for food webs in the freshwater environments inhabited by each kokanee population. The δ^15^N compositions of dissolved inorganic nitrogen that is assimilated by primary producers at the base of freshwater food webs is subject to a much wider range of processes (relative to DIC) that alter nitrogen isotopic compositions (for reviews see [31, 41, 138]). In this context, given the more recent collection date and location (interior regions that may have been logged or subjected to substantial urban development), it seems likely that, rather than feeding at a much higher trophic level, elevated δ^15^N for certain kokanee populations is the result of higher local δ^15^N baselines that have been altered through twentieth and twenty-first century anthropogenic activities [139, 140]. Therefore, while these δ^15^N data are not considered as a means of distinguishing between ecotypes for the purposes of our study, we include them here as they may be useful for future research on anthropogenic impacts such as urbanization and deforestation.

### Ancient DNA analysis

Table 1 summarizes the results of the aDNA analysis. Both *cytb* and D-loop were successfully amplified from each of the 10 samples included in the analysis. The results of the BLASTn searches and phylogenetic analyses indicate the *cytb* and D-loop sequences obtained from 9 of the 10 samples matched or closely resembled *O*. *nerka* references sequences (Figs 1 and 2 in S2 Text). Consequently, these samples were confidently identified as *O*. *nerka* (Table 1). The remaining sample, IUBC 5207 (ELS8), was identified as coho salmon (*O*. *kisutch*) as it yielded *cytb* and D-loop sequences that matched reference sequences from that species (Table 1; Figs 1 and 2 in S2 Text). This finding is consistent with the larger size of this vertebral specimen, relative to the others analyzed here, as well as its unusual isotopic composition, falling outside of the observed range for *O*. *nerka* δ^13^C (–13.6‰) and δ^15^N (+14.8‰) (Fig 1 in S1 Text). As sample IUBC 5207 was not identified as *O*. *nerka*, it was excluded from further analysis and discussion. DNA was amplified with both sex identification assays from 9 of the 10 salmonid remains. In total, 4 samples were identified as male and 4 were identified as female (Table 1). Two samples could not be assigned to a sex due to the assays generating conflicting results or one of the assays failing to amplify DNA (Table 1). No DNA was amplified from any of the blank extraction or negative PCR controls, indicating a lack of systematic contamination. The *cytb* and D-loop sequences obtained from each of the samples have been deposited in GenBank under accession numbers MN598987 to MN599006.

**Table 1:** Genetic species and sex identification results for the archaeological samples included in the ancient DNA analysis.

Isotope Lab Number	aDNA Lab Number	Archaeological Site	*clock1a*/*sdY* Assay Sex ID	D-loop/*sdY* Assay Sex ID	Consensus Sex ID	*cytb* Species ID	D-loop Species ID	Consensus Species ID
IUBC 5166	ELS1	Shields	Male	Male	Male	*O*. *nerka*	*O*. *nerka*	*O*. *nerka*
IUBC 5167	ELS6	Shields	PCR Failure	Female	Indeterminate	*O*. *nerka*	*O*. *nerka*	*O*. *nerka*
IUBC 5182	ELS7	Shields	Female	Female	Female	*O*. *nerka*	*O*. *nerka*	*O*. *nerka*
IUBC 5206	ELS2	Shields	Female	Male	Indeterminate	*O*. *nerka*	*O*. *nerka*	*O*. *nerka*
IUBC 5207	ELS8	Shields	Male	Male	Male	*O*. *kisutch*	*O*. *kisutch*	*O*. *kisutch*
IUBC 5215	ELS9	Bear Lake	Male	Male	Male	*O*. *nerka*	*O*. *nerka*	*O*. *nerka*
IUBC 5216	ELS3	Bear Lake	Female	Female	Female	*O*. *nerka*	*O*. *nerka*	*O*. *nerka*
IUBC 5218	ELS4	Bear Lake	Female	Female	Female	*O*. *nerka*	*O*. *nerka*	*O*. *nerka*
IUBC 5219	ELS10	Bear Lake	Female	Female	Female	*O*. *nerka*	*O*. *nerka*	*O*. *nerka*
IUBC 5226	ELS5	Bear Lake	Male	Male	Male	*O*. *nerka*	*O*. *nerka*	*O*. *nerka*

## Discussion

To demonstrate the utility of this approach, we undertook three case studies illustrating how this *O*. *nerka* δ^13^C baseline can be used in a range of research fields including archaeology, natural history, and conservation biology.

### Archaeology

Aquatic resources, and fish in particular, have been of tremendous importance both dietarily and culturally throughout much of the course of humanity’s evolution [141]. Consequently, understanding past fishing strategies—what, where, and how particular species were exploited—has long been a prominent archaeological research theme [142–144]. This is particularly true in North America’s Pacific Northwest where salmon and other fish have played a vital role in human cultural and dietary practices since the Late Pleistocene [12, 53, 55]. While numerous studies have demonstrated the importance of *O*. *nerka* to Indigenous fisheries in the Pacific Northwest [e.g., 59, 64, 66, 68], clear faunal evidence for the relative importance of sockeye *versus* kokanee has remained elusive.

The lack of information concerning the relative importance of sockeye and kokanee to Indigenous fisheries in the Pacific Northwest partially reflects methodological difficulties surrounding the differentiation of these ecotypes. As noted above (see Section Species identification of archaeological Pacific salmonid remains), Pacific salmonid vertebrae and most other elements cannot be confidently assigned to a species, let alone ecotype, through conventional morphology-based zooarchaeological approaches or a variety of other approaches. While useful for species identification, aDNA analysis and ZooMS likewise have limitations that hamper their ability to differentiate archaeological sockeye and kokanee specimens. In modern contexts, microsatellite and nuclear SNP loci are often used to assign *O*. *nerka* to populations [e.g., 145–147]. However, due to DNA degradation, the PCR amplification of these markers from archaeological specimens is prone to high rates of drop-out or is often not possible [e.g., 128, 148]. Although high-throughput sequencing methods are improving capacity for recovering genome-wide data useful for population assignment from ancient fish remains [149], these methods are expensive relative to isotopic approaches. Moreover, even when genome-wide data is available, genetically assigning bones from extinct populations to the correct ecotype may be difficult due to their polyphyly [150, 151]. Since they do not represent distinct lineages, genetically assigning individuals to the correct ecotype requires genetic reference data from local populations of the different ecotypes, which may be unavailable for extinct populations. In the case of ZooMS, type 1 collagen’s relatively slow rate of evolution amongst salmonids (ca. 6–12 amino acid substitutions per million years in *Oncorhynchus*) [71, 72] means recently diverged ecotypes, such as kokanee and sockeye (which diverged post-glacially [151]), cannot be differentiated by peptide mass fingerprinting techniques [e.g., 139, 152].

The applicability of our δ^13^C approach for assessing the ecotype of archaeological specimens requires a caveat. At the extreme end of their growth range, sockeye smolts can overlap in size with adult kokanee [153] and, because both have freshwater life histories, should therefore be indistinguishable based on δ^13^C. As adults and smolts can typically be differentiated morphologically, this issue would not present a problem for archival or modern ecological research. However, in cases where archaeological *O*. *nerka* bones are particularly small, this δ^13^C approach may not always be able to distinguish between adult kokanee and juvenile sockeye. Nevertheless, this approach would still provide evidence for the relative importance of freshwater *versus* marine resource use.

Based on the δ^13^C composition of archaeological bone collagen (Fig 3), we were able to assign all of the *O*. *nerka* specimens from the Shields and Bear Lake sites to the anadromous (sockeye) ecotype. Previous interpretations of whether these bones represent evidence of fishing strategies targeting kokanee, sockeye, both ecotypes, or other salmonids were inconclusive, in part because of the morphological and size overlap between vertebrae from different salmonids [154]. However, due to these sites’ proximity to Choelquoit Lake (known locally as Eagle or Big Eagle Lake), a known kokanee habitat, it was hypothesized that these bones might be derived from a kokanee fishery, assuming the ecotype also historically inhabited the lake [125, 154]. Our isotopic data not only provide novel evidence for the targeting of anadromous fish by Plateau Pithouse Tradition and Tsilhqot’in fisheries but also have broader implications for our understanding of salmon fisheries in the British Columbia Interior Plateau. In the case of the Bear Lake samples, our data contribute to the long-standing debate regarding the extent to which Tsilhqot’in fisheries were oriented towards resident lacustrine taxa as opposed to anadromous salmon [125, 155, 156]. Although our sample size is small, the identification of only sea-run sockeye at both sites do not support Matson and Magne’s [125] hypothesis that Tsilhqot’in fisheries may have been more freshwater-lake oriented than those of Plateau Pithouse Tradition peoples. At least in the case of *O*. *nerka*, our data indicate Tsilhqot’in and Plateau Pithouse Tradition fisheries may have targeted anadromous *O*. *nerka* to a similar degree. These data support Tyhurst’s [156] hypothesis that anadromous salmon were an important food resource for the Tsilhqot’in. At the same time, our data provide new support for early isotopic analyses of human remains that suggested people in the Chilcotin region had consumed large amounts of marine protein during the Late Holocene [157].

While interpreting the relative use of anadromous and potamodromous fish in the archaeological past, it is also important to remain cognizant of the broader range of economic, social, and sensorial factors that may have been taken into consideration when choosing between available fish stocks. For instance, there are significant differences in body size [153] and flavour profile [158, 159] between sockeye and kokanee. Moreover, these data could have further implications for reconstructing the seasonality of past fisheries because kokanee and sockeye migrate to spawn at slightly different times [137].

Our data also highlight the utility of conducting combined isotopic and aDNA analysis on single ancient fish bones. By applying both our *δ*^13^C approach and aDNA analysis to single vertebrae, we were able to generate more information about a single specimen than is possible with either method in isolation. While *δ*^13^C data provided information about archaeological specimens’ migratory behaviour, we were able to assign specimens to both a species and sex through aDNA analysis; information that contributes to a more nuanced understanding of past human fishing strategies. Moreover, through additional genetic analyses of ancient specimens isotopically identified as sockeye or kokanee, tracking natural and anthropogenic changes in the genetic diversity [e.g., 160] and phenotypic variation [e.g, 161] of these ecotypes is possible. Ultimately, by conducting multiple analyses on a single sample, this combined aDNA-isotopic approach will reduce the need for destructive sampling of different specimens for individual biomolecular analyses.

### Natural history collections

Globally, fish and other biological specimens archived in natural history collections represent an invaluable resource for reconstructing past species’ behaviours and environmental conditions [162–164]. The earliest specimens can be especially valuable for providing baseline evidence for species’ genetics, behaviour, and environmental conditions prior to the onset of major impacts from industrialization. However, early specimens may have lost their associated provenance information, or may have been collected at a time when fewer contextual details were typically recorded. In this context, methods for reconstructing aspects of a specimen’s life history may provide a useful tool for re-establishing key provenance or contextual details.

While collecting scales from sockeye and kokanee at museums, we observed a number of *O*. *nerka* specimens that did not have associated information regarding their ecotype or had limited provenance details. Some of these specimens were large enough to have been either kokanee or sockeye and many were collected in regions where these ecotypes are sympatric. We used scale δ^13^C from these behaviourally ambiguous *O*. *nerka* specimens to provide improved life history information for museum records. In total, we analyzed scales from 20 fish collected between 1926 and 1960 from 9 locations along a large latitudinal transect (Figs 1 and 3). Through our analyses, we were able to assign all specimens to either kokanee (*n* = 17) or sockeye (*n* = 3) behavioural types.

A number of specimens produced notable results. For instance, two fish from Osoyoos Lake collected in 1956 (IUBC 4380 and 4381) had museum labels suggesting that they were kokanee but were categorized more cautiously here as “unknowns” due to their large size (ca. 40 cm) for the drainage as well as a handwritten postscript in associated field notes that led to questions about certainty of their initial identification. At the time that these specimens were collected it was thought that human impacts on water flow in rivers in this region limited sockeye returns, a fact that may have influenced the biologists’ choice in identifying these specimens. Stable carbon isotope values indicate that these fish were, in fact, anadromous sockeye and provide a more complete record for these historical specimens, enhancing their potential value for future research. Application of this technique to additional museum specimens may identify extinct populations of sockeye or kokanee, allowing for the historic distribution of these ecotypes to be reconstructed.

### Conservation

Worldwide, freshwater environments are a key conservation focus due to their high biodiversity as well as their vulnerability to overfishing [165] and other human impacts [166]. In coastal regions of the North Pacific Ocean, kokanee and sockeye are a major focus of fisheries conservation efforts. In many places, monitoring programs are used to track the health and progress of rehabilitating *O*. *nerka* populations at a variety of scales and using a wide range of techniques. In areas where sockeye and kokanee populations are sympatric, having an independent and non-invasive (in the case of scales) means of distinguishing between these ecotypes may be important. In the past, genetic methods have been successfully used to differentiate kokanee and sockeye [147]. However, the genetic differentiation of kokanee from sockeye is confounded by the fact that these ecotypes not only interbreed [147] but can also undergo intergenerational shifts between anadromy and potamodromy [22]. In this context, stable carbon isotope analyses of collagen from *O*. *nerka* can provide a simple alternative method for establishing whether a fish was anadromous or potamodromous.

We measured the δ^13^C of bone and scale collagen from modern specimens collected during the 2018 spawning run on the Okanagan River between Skaha and Okanagan Lakes in British Columbia (Fig 1). Skaha Lake’s resident kokanee population has received long-term monitoring from fisheries management agencies and in 2003 was stocked with sockeye as part of conservation efforts aimed at reintroducing the anadromous ecotype to the region [167]. In this context, our δ^13^C approach could provide independent validation of other methods and would be particularly important if other methods result in uncertainty or conflicting estimates as may be the case when anadromous or potamodromous populations mix. Based on bone collagen δ^13^C, all 13 *O*. *nerka* specimens analyzed were identified as either potamodromous (*n* = 5) or anadromous (*n* = 8). A comparison of these isotopic ecotype identifications with forthcoming genetic assignments will provide a cost-effective (and therefore easily scalable) means for monitoring the extent to which ecotype information from genetic analyses reflects *O*. *nerka* migratory behaviour.

## Conclusion

Using the collagen δ^13^C baseline presented here, each case study has demonstrated some of the potential for using this approach for differentiating sockeye (anadromous) and kokanee (potamodromous) populations across the North Pacific Ocean and its coastal regions. Not only is this approach potentially less invasive (in using scales rather than otoliths) it relies on δ^13^C analyses, a well-established technique that is widely available at many commercial laboratories at a fraction of the cost of other methods. For this reason, our approach could provide a widely applicable alternative to conventional methods for ecologists and conservationists interested in identifying ecotypes for large numbers of *O*. *nerka*. Moreover, it could be possible to adapt this approach to other taxa as well provided they meet behavioural and environmental criteria (see Section Stable car bon isotopes in aquatic environments). For instance, other species for which the presence and relative importance of freshwater *versus* marine ecotypes could be discerned include cherry salmon (*Oncorhynchus masou*), cutthroat trout (*Oncorhynchus clarkii*), and rainbow trout (*Oncorhynchus mykiss*) in the Pacific Ocean; Atlantic salmon and brown trout (*Salmo trutta*) in the Atlantic Ocean; and Arctic char (*Salvelinus alpinus*) in the Arctic Ocean.

Given the considerable variability in freshwater food web δ^13^C, as this study shows, it is critical that the approach is ‘ground truthed’ with a large and geographically expansive baseline of δ^13^C data from individuals of known anadromous and potamodromous ecotypes. This is essential in order to confirm that δ^13^C of fish scale and bone collagen from freshwater environments within a given study region do not significantly overlap with their marine conspecifics. For retrospective applications, it is also important to remain cognizant of the potential for temporal shifts in aquatic δ^13^C baselines, particularly in freshwater ecosystems. For instance, as shown by samples from the Alouette Reservoir it is possible for environmental conditions (particularly those impacted by cultural eutrophication) to push δ^13^C baselines for freshwater specimens into the range observed for marine fish. Due to the context dependent nature of the factors that control aquatic δ^13^C baselines, it may be difficult or impossible to fully assess these kinds of issues *a priori* for past environments. Nonetheless, considering some of the key axes along which δ^13^C is known to vary in modern environments (e.g., carbon sources and cycling; for review see [31]) in the context of other relevant paleoenvironmental indicators, such as diatom, elemental, and isotopic records from sediment cores, may help to constrain interpretations.

This collagen δ^13^C approach to the differentiation of fish anadromy and potamodromy should also be particularly useful for archaeologists interested in understanding the relative importance for freshwater and marine resources, both for exploring ancient fisheries as well as generating more realistic isotopic baselines for paleodietary reconstructions using human isotopic compositions. While ZooMS and aDNA analysis offer a means of establishing from which taxon a bone originates, they are often unable to assign archaeological fish remains to an ecotype or do so in a cost-effective manner. While it is possible to establish this information using elemental concentration and isotopic (e.g., strontium and sulfur; see Section Introduction) analyses of otoliths, these elements are archaeologically rare and, relative to measuring the δ^13^C composition of archaeological bone collagen, require a more costly and laborious analytical process. Considering the broad significance of salmon and other anadromy-flexible species to human subsistence and cultural practices across the globe, wider adaptation of this approach to include other species could provide novel details about the seasonality of archaeological site use, clues about past fishing technologies, and possibly even reveal new evidence for trade in fish products.

## Supporting information

S1 Text Supporting information for the stable isotope analysis. (PDF)

S2 Text Supporting information for the ancient DNA analysis. (PDF)
