# Supplementary material for: Differentiating salmonid migratory ecotypes through stable isotope analysis of collagen: Archaeological and ecological applications
Source: PLoS One. 2020 Apr 28;15(4):e0232180. doi: 10.1371/journal.pone.0232180 (PMC7188214; doi:10.1371/journal.pone.0232180)
Supplement: S1 Text — (PDF) [file pone.0232180.s001.pdf]

S1 Text – Stable Isotope Analysis

δ^{13}C and δ^{15}N were measured with an elemental analyzer (EA) coupled via continuous flow to an isotope ratio mass spectrometer (CF-IRMS) at The University of British Columbia (UBC) (Vancouver, BC) and at Trent University (TU) (Peterborough, ON). A Vario MICRO cube EA coupled to an Isoprime IRMS (Elementar, Hanover, Germany) was used at UBC and n EA 300 (Eurovector, Pavia, Italy) coupled to a Horizon IRMS (Nu Instruments, Wrexham, UK) was used at TU. Isotopic measurements were calibrated relative to VPDB (δ^{13}C) and AIR (δ^{15}N) using USGS40 and USGS41 (Qi, et al. 2003) or USGS41a (Qi, et al. 2016) (Table 1). Internal check standards (Table 1) with known long-term averages were used to monitor analytical accuracy. Table 2 shows the standard deviations (1σ) and number of analyses for calibration standards for each analytical session. Table 3 shows mean and standard deviation (1σ) of δ^{13}C and δ^{15}N for check standards for all analytical sessions. Analytical uncertainty was calculated following (Szpak, et al. 2017). For δ^{13}C and δ^{15}N random errors ($uR_{(w)}$) was calculated to ±0.08 ‰ and ±0.15 ‰, respectively, systematic errors ($u_{(bias)}$) for δ^{13}C and δ^{15}N were calculated to be ±0.10 ‰ and ±0.15 ‰, respectively.. Standard uncertainty was calculated to be ±0.13 for δ^{13}C and ±0.21 for δ^{15}N. Isotopic composition, elemental concentration, and contextual data for samples analyzed in this study are shown in Table 5.

Table 1. Accepted δ values for calibrations standards and long-term averages (with 1σ) for collagen check standards used in this study.

Name	Material	Number	δ^{13}C (‰, VPDB)	δ^{15}N (‰, AIR)	Standard Type
USGS40	Glutamic acid	NA	−26.39	−4.52	Calibration standard
USGS41	Glutamic acid	NA	+37.63	+47.57	Calibration standard
USGS41a	Glutamic acid	NA	+36.55	+47.55	Calibration standard
MET	Methionine	1046	−28.62±0.11	−5.03±0.15	Check standard
SRM−1	Caribou bone collagen	461	−19.36±0.11	+1.81±0.10	Check standard
SRM−3	Commercial Gelatin	94	−15.30±0.17	+5.09±0.15	Check standard
SRM−14	Polar bear bone collagen	128	−13.66±0.06	+21.63±0.11	Check standard
SRM-15	Deer bone collagen	104	−26.88±0.05	+6.90±0.08	Check standard
SRM-16	Seal bone collagen	132	−14.81±0.10	+16.91±0.08	Check standard

Table 2. Standard deviations (1σ) for δ^{13}C and δ^{15}N of calibration standards for all analytical sessions included in this study.

**Run ID**	**Standard**	**Number**	**δ^{13}C (1σ)**	**δ^{15}N (1σ)**
CN19-13	USGS40	9	0.04	0.15
CN19-12	USGS40	9	0.02	0.17
CN19-09	USGS40	9	0.05	0.12
CN19-05	USGS40	9	0.07	0.16
CN19-02	USGS40	5	0.09	0.18
CN19-01	USGS40	4	0.07	0.24
CN18-16	USGS40	9	0.03	0.10
CN18-09	USGS40	11	0.07	0.05
CN18-08	USGS40	9	0.04	0.05
CN18-06	USGS40	9	0.06	0.05
CN17-32	USGS40	10	0.03	0.05
CN17-31	USGS40	11	0.05	0.03
CN17-30	USGS40	9	0.06	0.03
CN19-13	USGS41a	9	0.08	0.19
CN19-12	USGS41a	8	0.06	0.20
CN19-09	USGS41a	7	0.03	0.21
CN19-05	USGS41a	9	0.06	0.16
CN19-02	USGS41a	5	0.03	0.13
CN19-01	USGS41a	4	0.23	0.24
CN18-16	USGS41a	9	0.08	0.16
CN18-09	USGS41a	9	0.05	0.05
CN18-08	USGS41a	9	0.05	0.18
CN18-06	USGS41a	9	0.08	0.05
CN17-32	USGS41a	10	0.02	0.15
CN17-31	USGS41a	11	0.03	0.16
CN17-30	USGS41a	8	0.09	0.16

Table 3. Mean and standard deviation (1σ) of $\delta^{13}C$ and $\delta^{15}N$ for check standards for all analytical sessions.

Run ID	Standard	Number	$\delta^{13}C$ (1σ)	$\delta^{15}N$ (1σ)
CN19-13	MET	7	-28.61 ± 0.04	-5.16 ± 0.18
CN19-12	MET	7	-28.59 ± 0.03	-5.13 ± 0.15
CN19-09	MET	6	-28.57 ± 0.05	-5.23 ± 0.12
CN19-05	MET	7	-28.57 ± 0.03	-5.20 ± 0.25
CN19-01	MET	3	-28.64 ± 0.10	-5.19 ± 0.33
CN18-16	MET	7	-28.58 ± 0.03	-5.04 ± 0.14
CN18-09	MET	6	-28.63 ± 0.06	-5.04 ± 0.06
CN18-08	MET	7	-28.63 ± 0.04	-5.09 ± 0.13
CN18-06	MET	7	-28.56 ± 0.05	-5.09 ± 0.13
CN17-32	MET	8	-28.62 ± 0.03	-5.09 ± 0.10
CN17-31	MET	7	-28.62 ± 0.05	-5.07 ± 0.08
CN17-30	MET	7	-28.62 ± 0.06	-5.04 ± 0.04
CN17-18	SRM-1	4	-19.37 ± 0.02	1.77 ± 0.03
CN17-32	SRM-1	5	-19.37 ± 0.04	1.78 ± 0.02
CN17-31	SRM-1	6	-19.38 ± 0.03	1.78 ± 0.04
CN18-09	SRM-3	9	-15.23 ± 0.06	5.15 ± 0.09
CN18-08	SRM-3	6	-15.36 ± 0.13	5.02 ± 0.06
CN18-06	SRM-3	9	-15.38 ± 0.17	5.13 ± 0.09
CN19-02	SRM-14	5	-13.68 ± 0.05	21.80 ± 0.14
CN17-32	SRM-14	3	-13.66 ± 0.04	21.59 ± 0.05
CN17-31	SRM-14	5	-13.70 ± 0.07	21.55 ± 0.04
CN17-30	SRM-14	5	-13.68 ± 0.06	21.60 ± 0.06
CN19-13	SRM-15	6	-26.88 ± 0.02	6.94 ± 0.14
CN19-12	SRM-15	5	-26.88 ± 0.05	6.87 ± 0.19
CN19-09	SRM-15	6	-26.88 ± 0.03	6.94 ± 0.08
CN19-05	SRM-15	6	-26.88 ± 0.03	6.97 ± 0.12
CN19-02	SRM-15	3	-26.86 ± 0.03	6.77 ± 0.06
CN19-01	SRM-15	3	-26.94 ± 0.16	6.74 ± 0.38
CN18-09	SRM-15	6	-26.86 ± 0.03	6.83 ± 0.12
CN17-32	SRM-15	6	-26.90 ± 0.02	6.83 ± 0.07
CN17-31	SRM-15	3	-26.92 ± 0.01	6.80 ± 0.11
CN17-30	SRM-15	3	-26.88 ± 0.02	6.88 ± 0.01
CN19-13	SRM-16	5	-14.79 ± 0.02	16.96 ± 0.09
CN19-12	SRM-16	4	-14.80 ± 0.03	16.96 ± 0.13
CN19-09	SRM-16	3	-14.78 ± 0.07	17.01 ± 0.11
CN19-05	SRM-16	5	-14.80 ± 0.05	16.95 ± 0.12
CN18-16	SRM-16	5	-14.79 ± 0.03	16.97 ± 0.11
CN18-09	SRM-16	6	-14.78 ± 0.06	16.91 ± 0.08
CN18-08	SRM-16	5	-14.79 ± 0.03	16.85 ± 0.04
CN18-06	SRM-16	5	-14.79 ± 0.06	16.88 ± 0.04

CN17-32	SRM-16	6	-14.85 ± 0.05	16.85 ± 0.05
CN17-31	SRM-16	3	-14.88 ± 0.06	16.83 ± 0.05
CN17-30	SRM-16	3	-14.78 ± 0.02	16.89 ± 0.02

Table 4. Standard deviations (1σ) for all replicates.

Standard	Number	δ^{13}C (1σ)	δ^{15}N (1σ)
TEAL 1710	2	0.03	0.19
TEAL 1713	2	0.05	0.10
TEAL 1719	2	0.09	0.17
TEAL 3911	2	0.14	0.05
TEAL 3914	2	0.05	0.22
IUBC 4068	2	0.03	0.06
IUBC 4069	2	0.01	0.03
IUBC 4086	2	0.01	0.05
IUBC 4087	2	0.08	0.14
IUBC 4089	2	0.03	0.10
IUBC 4094	2	0.14	0.04
IUBC 4098	2	0.01	0.18
IUBC 4099	3	0.08	0.08
IUBC 4382	2	0.11	0.04
IUBC 5166	2	0.05	0.00
IUBC 5182	2	0.06	0.03
IUBC 5214	2	0.04	0.04
IUBC 5218	2	0.11	0.26
IUBC 5220	2	0.03	0.05
IUBC 5222	2	0.00	0.10
IUBC 5227	2	0.00	0.10
IUBC 5279	2	0.11	0.02
IUBC 5291	2	0.04	0.03
IUBC 5294	2	0.04	0.05
IUBC 5295	2	0.04	0.01
IUBC 5300	2	0.07	0.04
IUBC 5310	2	0.09	0.03
IUBC 5313	2	0.02	0.08
IUBC 5318	2	0.15	0.04
IUBC 5320	2	0.05	0.06
IUBC 5322	2	0.04	0.04
IUBC 5324	2	0.01	0.02
IUBC 5329	2	0.03	0.01
IUBC 5330	2	0.08	0.00
IUBC 5345	2	0.05	0.24
IUBC 5358	2	0.10	0.12

IUBC 5362	2	0.04	0.08
IUBC 5371	2	0.07	0.03
IUBC 5383	2	0.00	0.09
IUBC 5384	2	0.08	0.04
IUBC 5389	2	0.05	0.10
IUBC 5394	2	0.04	0.02
IUBC 5395	2	0.01	0.04
IUBC 5398	2	0.11	0.11
IUBC 5405	2	0.01	0.05
IUBC 5406	2	0.03	0.05
IUBC 5410	2	0.05	0.09
IUBC 5413	2	0.08	0.06
IUBC 5417	2	0.05	0.01
IUBC 6950	2	0.02	0.11
IUBC 6952	2	0.08	0.23
IUBC 6954	2	0.06	0.09
IUBC 6962	2	0.16	0.06
IUBC 6968	2	0.16	0.04

Table 5. Isotopic and elemental compositions as well and contextual details for samples included in this study. Samples with IUBC and TEAL prefixes were analyzed at TU and UBC, respectively. For data sourced from the literature see (Guiry, et al. 2016; Horii, et al. 2015; Kaeriyama, et al. 2004; Meeuwig and Peacock 2017; Molkentin, et al. 2015; Overman and Parrish 2001; Qin and Kaeriyama 2016; Satterfield and Finney 2002; Wang, et al. 2018; Welch and Parsons 1993). For sample donors: RBCM is the Royal British Columbia Museum (Victoria, BC, Canada), BBM is the Beaty Biodiversity Museum (Vancouver, BC Canada), ROM is the Royal Ontario Museum (Toronto, ON, Canada), FLNR is the Ministry of Forests, Lands and Natural Resource Operations (Penticton, BC, Canada), and XFN is the Xeni Gwet'in First Nation (curated at the Department of Anthropology University of British Columbia, Vancouver, BC Canada). Sample provided by coauthors are identified by their respective initials. Capture date is displayed as year-month-day (where information on date and month are unknown, a "00" is given). For collagen $\delta^{13}C$, asterisks mark samples for which collagen $\delta^{13}C$ values have been calculated based on previously published muscle data following Satterfield and Finney (2002). $\delta^{13}C_{cor}$ shows values corrected for formalin fixation, Suess effect, and muscle-collagen offset where applicable. For *a priori* ecotype: 1 is potamodromous, 2 is anadromous, 3 is archaeological unknowns, 4 is archival unknowns, and 5 is modern unknowns. For $\delta^{13}C$ ecotype ID; 1 is 1 is potamodromous and 2 is anadromous,

Source/Donor	Lab No.	Cat No	Site Name	Latitude	Longitude	Prov. State	Capture Date	Material	Col. Yld. %	Mus. $\delta^{13}C$	Mus. %C	Mus. %N	Mus. C:N	Mus. $\delta^{13}C$, lipid cor.	Col. $\delta^{13}C$	Form. cor.	Suess cor. (‰)	$\delta^{13}C_{cor}$	$\delta^{15}N$	Col. %C	Col. %N	Col. C:N	*A priori* ecotype	$\delta^{13}C$ ecotype ID	Notes
This study, FLNR	IUBC 5310	539	Alouette Reservoir	49.334	-122.418	BC	2013-09-25	Scale							-20.8	n	2.1	-18.7	6.0	41.8	15.2	3.21	1		
This study, FLNR	IUBC 5311	542	Alouette Reservoir	49.334	-122.418	BC	2013-09-25	Scale							-21.6	n	2.1	-19.5	8.1	42.1	15.6	3.14	1		
This study, FLNR	IUBC 5312	543	Alouette Reservoir	49.334	-122.418	BC	2013-09-25	Scale							-20.9	n	2.1	-18.8	6.3	42.8	15.4	3.24	1		
This study, FLNR	IUBC 5313	544	Alouette Reservoir	49.334	-122.418	BC	2013-09-25	Scale							-20.9	n	2.1	-18.8	6.2	43.0	15.2	3.31	1		
This study, FLNR	IUBC 5314	545	Alouette Reservoir	49.334	-122.418	BC	2013-09-25	Scale							-20.7	n	2.1	-18.7	6.0	42.8	15.4	3.24	1		
This study, FLNR	IUBC 5315	547	Alouette Reservoir	49.334	-122.418	BC	2013-09-25	Scale							-21.3	n	2.1	-19.2	6.2	42.7	15.1	3.29	1		
This study, FLNR	IUBC 5316	552	Alouette Reservoir	49.334	-122.418	BC	2013-09-25	Scale							-20.7	n	2.1	-18.6	6.0	42.8	15.4	3.25	1		
This study, FLNR	IUBC 5317	553	Alouette Reservoir	49.334	-122.418	BC	2013-09-25	Scale							-20.5	n	2.1	-18.4	6.3	42.7	15.4	3.24	1		
This study, FLNR	IUBC 5318	554	Alouette Reservoir	49.334	-122.418	BC	2013-09-25	Scale							-21.0	n	2.1	-19.0	6.6	42.6	15.4	3.24	1		
This study, FLNR	IUBC 5319	557	Alouette Reservoir	49.334	-122.418	BC	2013-09-25	Scale							-20.5	n	2.1	-18.4	6.9	42.5	15.3	3.25	1		
This study, FLNR	IUBC 5320	637	Alouette Reservoir	49.334	-122.418	BC	2013-09-26	Scale							-21.6	n	2.1	-19.5	6.3	42.8	15.1	3.29	1		
This study, FLNR	IUBC 5321	541	Alouette Reservoir	49.334	-122.418	BC	2013-09-26	Scale							-20.2	n	2.1	-18.1	6.4	41.8	15.0	3.25	1		
This study, FLNR	IUBC 5322	642	Alouette Reservoir	49.334	-122.418	BC	2013-09-26	Scale							-20.5	n	2.1	-18.5	6.3	43.3	15.0	3.36	1		
This study, FLNR	IUBC 5323	643	Alouette Reservoir	49.334	-122.418	BC	2013-09-26	Scale							-20.7	n	2.1	-18.6	6.3	47.8	16.8	3.32	1		
This study, FLNR	IUBC 5324	651	Alouette Reservoir	49.334	-122.418	BC	2013-09-26	Scale							-20.9	n	2.1	-18.8	6.1	47.7	17.3	3.22	1		
This study, FLNR	IUBC 5355	AR 2	Arrow Lake	50.442	-117.932	BC	2010-10-10	Scale							-23.0	n	1.9	-21.0	7.4	42.0	16.1	3.04	1		
This study, FLNR	IUBC 5356	AR5	Arrow Lake	50.442	-117.932	BC	2010-10-05	Scale							-22.1	n	1.9	-20.2	6.7	41.9	15.6	3.14	1		
This study, FLNR	IUBC 5357	AR10	Arrow Lake	50.442	-117.932	BC	2010-10-06	Scale							-22.2	n	1.9	-20.2	7.3	35.3	13.1	3.14	1		
This study, FLNR	IUBC 5358	AR16	Arrow Lake	50.442	-117.932	BC	2010-10-06	Scale							-22.9	n	1.9	-20.9	7.7	41.7	14.9	3.26	1		
This study, FLNR	IUBC 5359	AR17	Arrow Lake	50.442	-117.932	BC	2010-10-06	Scale							-21.8	n	1.9	-19.9	7.0	42.0	15.6	3.13	1		
This study, FLNR	IUBC 5360	AR18	Arrow Lake	50.442	-117.932	BC	2010-10-06	Scale							-22.2	n	1.9	-20.2	7.7	41.9	15.9	3.07	1		
This study, FLNR	IUBC 5361	AR20	Arrow Lake	50.442	-117.932	BC	2010-10-06	Scale							-22.4	n	1.9	-20.4	7.3	41.8	15.5	3.15	1		
This study, FLNR	IUBC 5362	AR21	Arrow Lake	50.442	-117.932	BC	2010-10-06	Scale							-21.9	n	1.9	-20.0	7.5	41.7	15.6	3.11	1		
This study, FLNR	IUBC 5363	AR25	Arrow Lake	50.442	-117.932	BC	2010-10-08	Scale							-24.3	n	1.9	-22.3	7.6	41.9	15.6	3.14	1		
This study, FLNR	IUBC 5364	AR32	Arrow Lake	50.442	-117.932	BC	2010-10-11	Scale							-24.4	n	1.9	-22.5	7.7	41.0	15.3	3.12	1		
This study, FLNR	IUBC 5365	AR50	Arrow Lake	50.442	-117.932	BC	2010-10-11	Scale							-23.7	n	1.9	-21.8	7.2	42.2	15.7	3.14	1		
This study, FLNR	IUBC 5366	AR51	Arrow Lake	50.442	-117.932	BC	2010-10-11	Scale							-22.4	n	1.9	-20.5	7.4	42.4	15.7	3.15	1		
This study, FLNR	IUBC 5367	AR52	Arrow Lake	50.442	-117.932	BC	2010-10-11	Scale							-23.8	n	1.9	-21.9	7.9	42.2	15.7	3.14	1		
This study, FLNR	IUBC 5368	AR53	Arrow Lake	50.442	-117.932	BC	2010-10-11	Scale							-23.2	n	1.9	-21.3	7.1	42.0	15.9	3.08	1		
This study, FLNR	IUBC 5369	AR54	Arrow Lake	50.442	-117.932	BC	2010-10-11	Scale							-22.1	n	1.9	-20.2	7.4	42.4	15.6	3.16	1		
This study, FLNR	IUBC 5370	AR56	Arrow Lake	50.442	-117.932	BC	2010-10-11	Scale							-22.7	n	1.9	-20.8	7.9	42.4	15.6	3.17	1		
This study, FLNR	IUBC 5305	CL-1	Christina Lake	49.139	-118.265	BC	2005-07-07	Scale							-27.2	n	1.7	-25.5	7.6	42.2	15.2	3.23	1		
This study, FLNR	IUBC 5307	CL-4	Christina Lake	49.139	-118.265	BC	2005-07-07	Scale							-27.7	n	1.7	-25.9	7.7	41.9	15.5	3.16	1		
This study, FLNR	IUBC 5308	CL-5	Christina Lake	49.139	-118.265	BC	2005-07-07	Scale							-28.6	n	1.7	-26.9	8.1	42.0	15.1	3.24	1		
This study, FLNR	IUBC 5401	1	Comox Lake	49.619	-125.166	BC	2009-07-31	Scale							-27.5	n	1.9	-25.6	6.5	46.1	16.9	3.18	1		
This study, FLNR	IUBC 5402	2	Comox Lake	49.619	-125.166	BC	2009-07-31	Scale							-28.2	n	1.9	-26.3	5.9	45.8	16.9	3.17	1		
This study, FLNR	IUBC 5403	3	Comox Lake	49.619	-125.166	BC	2009-07-31	Scale							-28.2	n	1.9	-26.3	5.9	32.3	12.0	3.13	1		
This study, FLNR	IUBC 5404	4	Comox Lake	49.619	-125.166	BC	2009-07-31	Scale							-28.2	n	1.9	-26.3	6.5	46.1	16.9	3.18	1		
This study, FLNR	IUBC 5405	5	Comox Lake	49.619	-125.166	BC	2009-07-31	Scale							-28.3	n	1.9	-26.4	5.9	45.6	17.0	3.12	1		
This study, FLNR	IUBC 5406	6	Comox Lake	49.619	-125.166	BC	2009-07-31	Scale							-28.4	n	1.9	-26.5	6.5	45.5	16.9	3.14	1		
This study, FLNR	IUBC 5407	7	Comox Lake	49.619	-125.166	BC	2009-07-31	Scale							-28.0	n	1.9	-26.1	6.2	41.7	15.0	3.24	1		
This study, FLNR	IUBC 5408	8	Comox Lake	49.619	-125.166	BC	2009-07-31	Scale							-28.1	n	1.9	-26.2	6.1	42.1	15.1	3.26	1		
This study, FLNR	IUBC 5409	9	Comox Lake	49.619	-125.166	BC	2009-07-31	Scale							-28.2	n	1.9	-26.3	5.8	40.7	14.7	3.23	1		
This study, FLNR	IUBC 5410	10	Comox Lake	49.619	-125.166	BC	2009-07-31	Scale							-27.2	n	1.9	-25.3	6.3	41.8	15.1	3.24	1		
This study, FLNR	IUBC 5411	11	Comox Lake	49.619	-125.166	BC	2009-07-31	Scale							-28.2	n	1.9	-26.3	6.5	42.0	15.1	3.24	1		
This study, FLNR	IUBC 5412	13	Comox Lake	49.619	-125.166	BC	2009-07-31	Scale							-28.6	n	1.9	-26.7	6.7	41.9	15.2	3.22	1		
This study, FLNR	IUBC 5413	15	Comox Lake	49.619	-125.166	BC	2009-07-31	Scale							-28.2	n	1.9	-26.3	5.8	42.2	15.1	3.25	1		
This study, FLNR	IUBC 5414	16	Comox Lake	49.619	-125.166	BC	2009-07-31	Scale							-28.0	n	1.9	-26.1	6.4	42.3	15.2	3.24	1		
This study, FLNR	IUBC 5415	17	Comox Lake	49.619	-125.166	BC	2009-07-31	Scale							-28.2	n	1.9	-26.3	6.5	42.3	15.2	3.25	1		
This study, FLNR	IUBC 5417	25	Comox Lake	49.619	-125.166	BC	2009-07-31	Scale							-28.2	n	1.9	-26.3	6.1	43.3	15.6	3.24	1		

Source/Donor	Lab No.	Cat No	Site Name	Latitude	Longitude	Prov. State	Capture Date	Material	Col. Yld. %	Mus. $\delta^{13}C$	Mus. %C	Mus. %N	Mus. C:N	Mus. $\delta^{13}C$, lipid cor.	Col. $\delta^{13}C$	Form. cor.	Suess cor. (‰)	$\delta^{13}C_{cor}$	$\delta^{15}N$	Col. %C	Col. %N	Col. C:N	*A priori* ecotype	$\delta^{13}C$ ecotype ID	Notes
This study, FLNR	IUBC 5418	26	Comox Lake	49.619	-125.166	BC	2009-07-31	Scale							-28.0	n	1.9	-26.1	6.1	42.9	15.6	3.21	1		
This study, FLNR	IUBC 5419	33	Comox Lake	49.619	-125.166	BC	2009-07-31	Scale							-28.2	n	1.9	-26.3	5.9	41.0	14.6	3.27	1		
This study, FLNR	IUBC 5420	36	Comox Lake	49.619	-125.166	BC	2009-07-31	Scale							-28.2	n	1.9	-26.3	6.2	42.2	14.8	3.32	1		
This study, FLNR	IUBC 5421	37	Comox Lake	49.619	-125.166	BC	2009-07-31	Scale							-28.3	n	1.9	-26.4	6.1	30.9	11.0	3.28	1		
This study, FLNR	IUBC 5343	56478	Cowichan Lake	48.883	-124.299	BC	2010-07-07	Scale							-27.1	n	1.9	-25.2	7.0	43.8	15.3	3.34	1		
This study, FLNR	IUBC 5345	56480	Cowichan Lake	48.883	-124.299	BC	2010-07-07	Scale							-27.7	n	1.9	-25.8	6.6	43.1	15.3	3.29	1		
This study, FLNR	IUBC 5346	56482	Cowichan Lake	48.883	-124.299	BC	2010-07-07	Scale							-27.7	n	1.9	-25.7	7.1	39.2	14.1	3.24	1		
This study, FLNR	IUBC 5347	56483	Cowichan Lake	48.883	-124.299	BC	2010-07-07	Scale							-27.4	n	1.9	-25.4	6.8	42.1	15.3	3.20	1		
This study, FLNR	IUBC 5349	56485	Cowichan Lake	48.883	-124.299	BC	2010-07-07	Scale							-27.6	n	1.9	-25.7	7.0	42.2	15.4	3.20	1		
This study, FLNR	IUBC 5350	56486	Cowichan Lake	48.883	-124.299	BC	2010-07-07	Scale							-27.4	n	1.9	-25.5	6.6	39.5	14.3	3.22	1		
This study, FLNR	IUBC 5352	56488	Cowichan Lake	48.883	-124.299	BC	2010-07-07	Scale							-26.5	n	1.9	-24.6	7.0	40.6	14.9	3.16	1		
This study, FLNR	IUBC 5353	56489	Cowichan Lake	48.883	-124.299	BC	2010-07-07	Scale							-24.9	n	1.9	-22.9	6.9	42.8	15.2	3.28	1		
This study, FLNR	IUBC 5354	56817	Cowichan Lake	48.883	-124.299	BC	2010-07-07	Scale							-27.7	n	1.9	-25.8	7.3	42.1	15.5	3.18	1		
This study, ROM	IUBC 4100	014205	Cultus Lake	49.053	-121.986	BC	1930-00-00	Scale							-30.0	y	0.2	-29.3	11.2	42.0	13.0	3.78	1		C:N unacceptable
This study, ROM	IUBC 4101	014205	Cultus Lake	49.053	-121.986	BC	1930-00-00	Scale							-31.4	y	0.2	-30.7	8.9	41.6	12.7	3.82	1		C:N unacceptable
This study, ROM	IUBC 4102	014205	Cultus Lake	49.053	-121.986	BC	1930-00-00	Scale							-31.5	y	0.2	-30.8	8.8	41.5	13.1	3.70	1		C:N unacceptable
This study, ROM	IUBC 4103	014205	Cultus Lake	49.053	-121.986	BC	1930-00-00	Scale							-31.5	y	0.2	-30.7	8.7	42.0	12.8	3.84	1		C:N unacceptable
This study, ROM	IUBC 4094	010665	Driftwood River	55.872	-126.485	BC	1938-08-00	Scale							-27.8	y	0.3	-27.0	7.9	42.2	14.2	3.48	1		
This study, ROM	IUBC 4095	010666	Driftwood River	55.872	-126.485	BC	1938-08-16	Scale							-28.0	y	0.3	-27.2	9.0	41.9	13.9	3.50	1		
This study, ROM	IUBC 4096	010667	Driftwood River	55.872	-126.485	BC	1938-08-16	Scale							-28.1	y	0.3	-27.3	8.6	41.5	14.1	3.44	1		
This study, BBM	IUBC 4379	11-0348	Easter Lake	Unknown	Unknown	NWT	1972-09-11	Scale							-25.5	y	0.8	-24.2	8.9	42.3	14.0	3.54	1		
This study, BBM	IUBC 4374	11-0142	Eutsuk Lake	53.245	-126.516	BC	1998-09-16	Scale							-26.2	y	1.5	-24.3	7.1	42.2	14.5	3.41	1		
This study, BBM	IUBC 4375	11-0142	Eutsuk Lake	53.245	-126.516	BC	1998-09-16	Scale							-25.8	y	1.5	-23.8	6.7	39.6	13.6	3.39	1		
This study, BBM	IUBC 4376	11-0142	Eutsuk Lake	53.245	-126.516	BC	1998-09-16	Scale							-26.0	y	1.5	-24.1	6.6	42.1	14.6	3.35	1		
This study, BBM	IUBC 4378	11-0142	Eutsuk Lake	53.245	-126.516	BC	1998-09-16	Scale							-26.5	y	1.5	-24.5	7.5	40.6	13.6	3.47	1		
This study, FLNR	IUBC 5275	K-1	Kalamalka Lake	50.174	-119.331	BC	2004-09-08	Scale							-25.1	n	1.7	-23.5	12.4	41.6	15.1	3.21	1		
This study, FLNR	IUBC 5279	K-5	Kalamalka Lake	50.174	-119.331	BC	2004-09-08	Scale							-25.0	n	1.7	-23.3	12.4	42.1	15.5	3.17	1		
This study, FLNR	IUBC 5280	K-6	Kalamalka Lake	50.174	-119.331	BC	2004-09-08	Scale							-25.2	n	1.7	-23.5	12.9	42.0	15.3	3.19	1		
This study, FLNR	IUBC 5281	K-7	Kalamalka Lake	50.174	-119.331	BC	2004-09-08	Scale							-25.4	n	1.7	-23.7	12.5	42.2	15.7	3.13	1		
This study, FLNR	IUBC 5282	K-8	Kalamalka Lake	50.174	-119.331	BC	2004-09-08	Scale							-25.3	n	1.7	-23.6	12.1	42.7	15.2	3.27	1		
This study, FLNR	IUBC 5283	K-9	Kalamalka Lake	50.174	-119.331	BC	2004-09-08	Scale							-25.2	n	1.7	-23.5	11.9	39.4	14.6	3.14	1		
This study, FLNR	IUBC 5371	10	Kinbasket Lake	52.156	-118.453	BC	2014-07-26	Scale							-26.0	n	2.1	-23.8	6.6	42.1	15.5	3.16	1		
This study, FLNR	IUBC 5372	11	Kinbasket Lake	52.156	-118.453	BC	2014-07-26	Scale							-25.5	n	2.1	-23.3	5.8	42.0	15.6	3.14	1		
This study, FLNR	IUBC 5373	12	Kinbasket Lake	52.156	-118.453	BC	2014-07-26	Scale							-26.3	n	2.1	-24.2	5.6	42.0	15.5	3.16	1		
This study, FLNR	IUBC 5374	14	Kinbasket Lake	52.156	-118.453	BC	2014-07-26	Scale							-25.9	n	2.1	-23.8	6.6	41.9	15.4	3.17	1		
This study, FLNR	IUBC 5375	26	Kinbasket Lake	52.156	-118.453	BC	2014-07-26	Scale							-25.6	n	2.1	-23.5	5.6	42.9	15.6	3.20	1		
This study, FLNR	IUBC 5376	40	Kinbasket Lake	52.156	-118.453	BC	2014-07-26	Scale							-26.0	n	2.1	-23.8	5.6	42.6	15.6	3.18	1		
This study, FLNR	IUBC 5377	42	Kinbasket Lake	52.156	-118.453	BC	2014-07-26	Scale							-26.4	n	2.1	-24.3	6.9	42.8	15.7	3.18	1		
This study, FLNR	IUBC 5378	59	Kinbasket Lake	52.156	-118.453	BC	2014-07-26	Scale							-26.1	n	2.1	-24.0	7.3	42.4	15.7	3.14	1		
This study, FLNR	IUBC 5379	60	Kinbasket Lake	52.156	-118.453	BC	2014-07-27	Scale							-26.1	n	2.1	-24.0	6.3	42.6	15.6	3.20	1		
This study, FLNR	IUBC 5380	62	Kinbasket Lake	52.156	-118.453	BC	2014-07-27	Scale							-26.5	n	2.1	-24.4	6.8	41.6	15.4	3.15	1		
This study, FLNR	IUBC 5381	63	Kinbasket Lake	52.156	-118.453	BC	2014-07-27	Scale							-26.5	n	2.1	-24.4	6.7	41.8	15.2	3.20	1		
This study, FLNR	IUBC 5382	64	Kinbasket Lake	52.156	-118.453	BC	2014-07-27	Scale							-26.5	n	2.1	-24.4	6.7	42.7	15.7	3.17	1		
This study, FLNR	IUBC 5383	65	Kinbasket Lake	52.156	-118.453	BC	2014-07-27	Scale							-26.1	n	2.1	-24.0	6.6	42.2	15.6	3.16	1		
This study, FLNR	IUBC 5384	78	Kinbasket Lake	52.156	-118.453	BC	2014-07-27	Scale							-26.2	n	2.1	-24.1	7.3	42.4	15.4	3.21	1		
This study, FLNR	IUBC 5385	83	Kinbasket Lake	52.156	-118.453	BC	2014-07-27	Scale							-26.4	n	2.1	-24.3	6.6	42.5	15.5	3.20	1		
This study, RBCM	IUBC 3893	942-017-001, 00284, 5	Lac la Hache	51.833	-121.517	BC	1942-10-04	Scale							-23.8	y	0.3	-22.9	12.4	41.1	14.5	3.32	1		
This study, RBCM	IUBC 3894	942-017-001, 00284, 5	Lac la Hache	51.833	-121.517	BC	1942-10-04	Scale							-23.8	y	0.3	-22.9	12.7	41.7	14.3	3.40	1		
This study, RBCM	IUBC 3895	942-017-001, 00284, 5	Lac la Hache	51.833	-121.517	BC	1942-10-04	Scale							-23.7	y	0.3	-22.9	12.1	41.5	14.7	3.29	1		
This study, RBCM	IUBC 3896	942-017-001, 00284, 5	Lac la Hache	51.833	-121.517	BC	1942-10-04	Scale							-23.9	y	0.3	-23.1	12.6	42.3	14.6	3.38	1		

Source/Donor	Lab No.	Cat No	Site Name	Latitude	Longitude	Prov. State	Capture Date	Material	Col. Yld. %	Mus. $\delta^{13}C$	Mus. %C	Mus. %N	Mus. C:N	Mus. $\delta^{13}C$, lipid cor.	Col. $\delta^{13}C$	Form. cor.	Suess cor. (‰)	$\delta^{13}C_{cor}$	$\delta^{15}N$	Col. %C	Col. %N	Col. C:N	*A priori* ecotype	$\delta^{13}C$ ecotype ID	Notes
This study, BBM	IUBC 4366	55-19	Lower Arrow Lake	49.780	-118.800	BC	1949-08-16	Scale							-26.3	y	0.4	-25.4	7.2	42.3	14.0	3.52	1		
This study, RBCM	IUBC 3891	988-00023-002, fish 1	Lucas Lake	53.583	-125.167	BC	1989-01-24	Scale							-26.2	y	1.2	-24.5	10.8	41.5	14.8	3.26	1		
This study, RBCM	IUBC 3892	988-00023-002, fish 2	Lucas Lake	53.583	-125.167	BC	1989-01-24	Scale							-26.7	y	1.2	-25.0	10.7	41.6	14.5	3.33	1		
This study, ROM	IUBC 4065	006135	McRae Creek	49.104	-118.234	BC	1928-06-26	Scale							-27.9	y	0.2	-27.1	7.7	44.2	14.3	3.61	1		
This study, FLNR	TEAL 1723	3	Middle Vernon Creek	50.048	-119.406	BC	2018-10-13	Bone, vertebra	20.2						-26.2	n	2.3	-23.9	14.5	45.9	17.2	3.11	1		
This study, FLNR	TEAL 1724	6	Middle Vernon Creek	50.048	-119.406	BC	2018-10-13	Bone, vertebra	22.0						-27.3	n	2.3	-25.0	14.4	47.2	17.3	3.19	1		
This study, FLNR	TEAL 1725	7	Middle Vernon Creek	50.048	-119.406	BC	2018-10-13	Bone, vertebra	20.6						-27.1	n	2.3	-24.8	14.5	46.7	17.2	3.16	1		
This study, FLNR	TEAL 1726	13	Middle Vernon Creek	50.048	-119.406	BC	2018-10-13	Bone, vertebra	23.3						-26.5	n	2.3	-24.2	14.6	46.9	17.0	3.22	1		
This study, FLNR	TEAL 1727	18	Middle Vernon Creek	50.048	-119.406	BC	2018-10-13	Bone, vertebra	23.4						-26.5	n	2.3	-24.2	14.5	46.8	17.1	3.19	1		
This study, FLNR	TEAL 1728	19	Middle Vernon Creek	50.048	-119.406	BC	2018-10-13	Bone, vertebra	20.4						-26.5	n	2.3	-24.2	14.3	46.4	17.0	3.19	1		
This study, FLNR	TEAL 1729	22	Middle Vernon Creek	50.048	-119.406	BC	2018-10-13	Bone, vertebra	21.6						-26.7	n	2.3	-24.4	14.4	47.2	17.6	3.13	1		
This study, FLNR	TEAL 1730	28	Middle Vernon Creek	50.048	-119.406	BC	2018-10-13	Bone, vertebra	22.2						-27.5	n	2.3	-25.2	15.3	47.5	17.3	3.21	1		
This study, FLNR	TEAL 1731	33	Middle Vernon Creek	50.048	-119.406	BC	2018-10-13	Bone, vertebra	22.9						-27.2	n	2.3	-24.9	14.6	47.7	17.4	3.21	1		
This study, FLNR	TEAL 1732	40	Middle Vernon Creek	50.048	-119.406	BC	2018-10-16	Bone, vertebra	22.9						-26.9	n	2.3	-24.6	14.3	47.7	17.5	3.18	1		
This study, FLNR	TEAL 1733	43	Middle Vernon Creek	50.048	-119.406	BC	2018-10-16	Bone, vertebra	21.6						-27.0	n	2.3	-24.6	14.6	47.1	17.4	3.16	1		
This study, FLNR	TEAL 1734	54	Middle Vernon Creek	50.048	-119.406	BC	2018-10-16	Bone, vertebra	20.5						-27.1	n	2.3	-24.8	14.2	46.6	17.6	3.09	1		
This study, FLNR	TEAL 1735	56	Middle Vernon Creek	50.048	-119.406	BC	2018-10-16	Bone, vertebra	21.3						-27.2	n	2.3	-24.9	14.8	46.8	17.4	3.13	1		
This study, FLNR	TEAL 1736	62	Middle Vernon Creek	50.048	-119.406	BC	2018-10-16	Bone, vertebra	22.1						-26.9	n	2.3	-24.5	14.6	47.3	17.2	3.21	1		
This study, FLNR	TEAL 1737	70	Middle Vernon Creek	50.048	-119.406	BC	2018-10-16	Bone, vertebra	21.2						-27.1	n	2.3	-24.8	14.2	47.3	17.3	3.18	1		
This study, FLNR	TEAL 1738	71	Middle Vernon Creek	50.048	-119.406	BC	2018-10-16	Bone, vertebra	23.5						-27.3	n	2.3	-25.0	15.2	47.7	17.3	3.21	1		
This study, ROM	IUBC 4069	006092	Nelson Warf	49.498	-117.300	BC	1928-06-19	Scale							-26.4	y	0.2	-25.7	9.1	42.8	13.9	3.60	1		
This study, ROM	IUBC 4070	006093	Nelson Warf	49.498	-117.300	BC	1928-06-19	Scale							-26.3	y	0.2	-25.6	9.6	42.3	14.2	3.48	1		
This study, ROM	IUBC 4071	006094	Nelson Warf	49.498	-117.300	BC	1928-06-19	Scale							-26.3	y	0.2	-25.6	9.0	42.9	14.2	3.53	1		
This study, ROM	IUBC 4072	006095	Nelson Warf	49.498	-117.300	BC	1928-06-19	Scale							-26.4	y	0.2	-25.6	8.7	42.3	14.3	3.46	1		
This study, ROM	IUBC 4073	006096	Nelson Warf	49.498	-117.300	BC	1928-06-19	Scale							-26.5	y	0.2	-25.8	9.0	43.0	13.8	3.62	1		
This study, ROM	IUBC 4074	006097	Nelson Warf	49.498	-117.300	BC	1928-06-19	Scale							-26.6	y	0.2	-25.9	9.0	42.9	14.3	3.49	1		
This study, ROM	IUBC 4075	006098	Nelson Warf	49.498	-117.300	BC	1928-06-19	Scale							-26.3	y	0.2	-25.5	9.1	42.9	14.5	3.44	1		
This study, ROM	IUBC 4076	006099	Nelson Warf	49.498	-117.300	BC	1928-06-19	Scale							-26.7	y	0.2	-25.9	9.5	43.5	14.2	3.56	1		
This study, FLNR	IUBC 5290	37871	Okanagan Lake	49.834	-119.524	BC	1998-09-15	Scale							-24.5	n	1.5	-23.0	11.2	42.4	15.4	3.20	1		
This study, FLNR	IUBC 5291	37878	Okanagan Lake	49.834	-119.524	BC	1998-00-00	Scale							-24.2	n	1.5	-22.7	11.6	42.2	15.6	3.16	1		
This study, FLNR	IUBC 5292	37894	Okanagan Lake	49.834	-119.524	BC	1998-00-00	Scale							-24.7	n	1.5	-23.3	11.2	42.8	15.4	3.25	1		
This study, FLNR	IUBC 5293	37912	Okanagan Lake	49.834	-119.524	BC	1998-00-00	Scale							-24.7	n	1.5	-23.3	10.9	42.3	15.0	3.28	1		
This study, FLNR	IUBC 5294	37913	Okanagan Lake	49.834	-119.524	BC	1998-00-00	Scale							-24.6	n	1.5	-23.1	12.1	42.3	15.6	3.16	1		
This study, FLNR	IUBC 5295	37925	Okanagan Lake	49.834	-119.524	BC	1998-00-00	Scale							-24.6	n	1.5	-23.2	11.7	42.0	15.6	3.14	1		
This study, FLNR	IUBC 5296	NA	Okanagan Lake	49.834	-119.524	BC	1998-00-00	Scale							-25.0	n	1.5	-23.5	11.7	41.9	15.5	3.16	1		
This study, FLNR	IUBC 5297	37927	Okanagan Lake	49.834	-119.524	BC	1998-00-00	Scale							-24.9	n	1.5	-23.5	11.7	42.2	15.3	3.22	1		
This study, FLNR	IUBC 5298	37928	Okanagan Lake	49.834	-119.524	BC	1998-00-00	Scale							-24.9	n	1.5	-23.4	11.9	42.2	15.6	3.16	1		
This study, FLNR	IUBC 5299	37949	Okanagan Lake	49.834	-119.524	BC	1998-00-00	Scale							-24.5	n	1.5	-23.0	10.7	42.3	15.6	3.17	1		
This study, FLNR	IUBC 5300	37950	Okanagan Lake	49.834	-119.524	BC	1998-00-00	Scale							-24.6	n	1.5	-23.2	11.5	41.8	15.5	3.15	1		
This study, FLNR	IUBC 5301	37951	Okanagan Lake	49.834	-119.524	BC	1998-00-00	Scale							-24.9	n	1.5	-23.5	12.2	42.3	15.5	3.18	1		
This study, FLNR	IUBC 5302	37973	Okanagan Lake	49.834	-119.524	BC	1998-00-00	Scale							-24.7	n	1.5	-23.2	11.3	41.8	15.5	3.15	1		
This study, FLNR	IUBC 5303	38060	Okanagan Lake	49.834	-119.524	BC	1998-00-00	Scale							-24.1	n	1.5	-22.7	10.9	42.1	15.5	3.17	1		
This study, FLNR	IUBC 5304	38076	Okanagan Lake	49.834	-119.524	BC	1998-00-00	Scale							-24.1	n	1.5	-22.7	11.4	42.0	15.5	3.16	1		
This study, FLNR	IUBC 5422	1	Okanagan Lake	49.834	-119.524	BC	2011-08-31	Scale							-24.7	n	2.0	-22.8	12.7	42.9	15.4	3.25	1		
This study, FLNR	IUBC 5423	2	Okanagan Lake	49.834	-119.524	BC	2011-08-31	Scale							-24.3	n	2.0	-22.3	11.7	43.2	15.4	3.27	1		
This study, FLNR	IUBC 5424	3	Okanagan Lake	49.834	-119.524	BC	2011-08-31	Scale							-25.4	n	2.0	-23.4	11.5	37.6	13.2	3.31	1		
This study, FLNR	IUBC 5425	4	Okanagan Lake	49.834	-119.524	BC	2011-08-31	Scale							-25.0	n	2.0	-23.0	11.7	45.5	16.6	3.19	1		
This study, FLNR	IUBC 5426	5	Okanagan Lake	49.834	-119.524	BC	2011-08-31	Scale							-25.3	n	2.0	-23.3	12.1	45.5	16.6	3.20	1		
This study, FLNR	IUBC 5427	6	Okanagan Lake	49.834	-119.524	BC	2011-08-31	Scale							-25.5	n	2.0	-23.5	11.6	45.4	16.2	3.26	1		
This study, FLNR	IUBC 5428	7	Okanagan Lake	49.834	-119.524	BC	2011-08-31	Scale							-25.5	n	2.0	-23.5	11.8	44.8	16.2	3.22	1		

Source/Donor	Lab No.	Cat No	Site Name	Latitude	Longitude	Prov. State	Capture Date	Material	Col. Yld. %	Mus. $\delta^{13}C$	Mus. %C	Mus. %N	Mus. C:N	Mus. $\delta^{13}C$, lipid cor.	Col. $\delta^{13}C$	Form. cor.	Suess cor. (‰)	$\delta^{13}C_{cor}$	$\delta^{15}N$	Col. %C	Col. %N	Col. C:N	*A priori* ecotype	$\delta^{13}C$ ecotype ID	Notes
This study, FLNR	IUBC 5429	8	Okanagan Lake	49.834	-119.524	BC	2011-08-31	Scale							-25.2	n	2.0	-23.2	11.8	45.0	16.4	3.19	1		
This study, FLNR	IUBC 5430	9	Okanagan Lake	49.834	-119.524	BC	2011-08-31	Scale							-25.5	n	2.0	-23.5	11.7	45.2	16.6	3.17	1		
This study, FLNR	IUBC 5431	10	Okanagan Lake	49.834	-119.524	BC	2011-08-31	Scale							-25.4	n	2.0	-23.5	11.8	44.9	16.7	3.13	1		
This study, BBM	IUBC 4363	55-0139	Pothole Lake	49.930	-120.280	BC	1955-07-07	Scale							-33.2	y	0.5	-32.2	8.7	42.9	13.4	3.74	1		C:N unacceptable
This study, BBM	IUBC 4364	55-0139	Pothole Lake	49.930	-120.280	BC	1955-07-07	Scale							-33.5	y	0.5	-32.5	8.2	42.6	13.5	3.67	1		C:N unacceptable
This study, BBM	IUBC 4365	55-0139	Pothole Lake	49.930	-120.280	BC	1955-07-07	Scale							-29.3	y	0.5	-28.3	9.4	42.7	14.1	3.53	1		
This study, FLNR	IUBC 5386	1	Revelstoke Lake	51.149	-118.202	BC	2014-07-28	Scale							-27.2	n	2.1	-25.1	6.3	42.4	15.5	3.20	1		
This study, FLNR	IUBC 5387	3	Revelstoke Lake	51.149	-118.202	BC	2014-07-28	Scale							-26.9	n	2.1	-24.8	6.2	42.3	15.6	3.16	1		
This study, FLNR	IUBC 5388	5	Revelstoke Lake	51.149	-118.202	BC	2014-07-28	Scale							-26.8	n	2.1	-24.7	5.7	42.2	15.6	3.15	1		
This study, FLNR	IUBC 5389	16	Revelstoke Lake	51.149	-118.202	BC	2014-07-28	Scale							-26.7	n	2.1	-24.6	6.0	42.1	15.5	3.16	1		
This study, FLNR	IUBC 5390	18	Revelstoke Lake	51.149	-118.202	BC	2014-07-28	Scale							-26.8	n	2.1	-24.7	6.2	42.1	15.5	3.17	1		
This study, FLNR	IUBC 5391	31	Revelstoke Lake	51.149	-118.202	BC	2014-07-28	Scale							-26.3	n	2.1	-24.2	5.8	41.9	15.6	3.12	1		
This study, FLNR	IUBC 5392	32	Revelstoke Lake	51.149	-118.202	BC	2014-07-28	Scale							-26.6	n	2.1	-24.5	6.2	45.4	16.9	3.13	1		
This study, FLNR	IUBC 5393	33	Revelstoke Lake	51.149	-118.202	BC	2014-07-28	Scale							-27.0	n	2.1	-24.9	6.5	44.0	16.3	3.15	1		
This study, FLNR	IUBC 5394	37	Revelstoke Lake	51.149	-118.202	BC	2014-07-28	Scale							-27.1	n	2.1	-24.9	6.6	45.9	16.9	3.16	1		
This study, FLNR	IUBC 5395	38	Revelstoke Lake	51.149	-118.202	BC	2014-07-28	Scale							-26.7	n	2.1	-24.6	6.4	45.7	17.1	3.11	1		
This study, FLNR	IUBC 5396	39	Revelstoke Lake	51.149	-118.202	BC	2014-07-28	Scale							-26.7	n	2.1	-24.6	6.4	45.7	17.0	3.13	1		
This study, FLNR	IUBC 5397	42	Revelstoke Lake	51.149	-118.202	BC	2014-07-28	Scale							-26.7	n	2.1	-24.6	6.2	45.8	17.1	3.12	1		
This study, FLNR	IUBC 5398	43	Revelstoke Lake	51.149	-118.202	BC	2014-07-28	Scale							-26.9	n	2.1	-24.8	6.3	45.7	17.1	3.12	1		
This study, FLNR	IUBC 5399	50	Revelstoke Lake	51.149	-118.202	BC	2014-07-30	Scale							-27.1	n	2.1	-25.0	6.4	45.9	16.8	3.18	1		
This study, FLNR	IUBC 5400	61	Revelstoke Lake	51.149	-118.202	BC	2014-07-30	Scale							-27.0	n	2.1	-24.9	5.9	46.0	16.7	3.21	1		
This study, BBM	IUBC 4367	56-620	Sand Lake	54.950	-128.970	BC	1956-08-18	Scale							-30.5	y	0.5	-29.5	10.1	43.1	13.9	3.61	1		
This study, BBM	IUBC 4368	56-620	Sand Lake	54.950	-128.970	BC	1956-08-18	Scale							-29.7	y	0.5	-28.7	9.8	42.3	14.2	3.47	1		
This study, BBM	IUBC 4369	56-620	Sand Lake	54.950	-128.970	BC	1956-08-18	Scale							-30.4	y	0.5	-29.4	9.5	42.9	13.8	3.61	1		
This study, BBM	IUBC 4370	56-620	Sand Lake	54.950	-128.970	BC	1956-08-18	Scale							-30.4	y	0.5	-29.4	9.3	42.7	13.9	3.57	1		
This study, BBM	IUBC 4371	56-620	Sand Lake	54.950	-128.970	BC	1956-08-18	Scale							-30.8	y	0.5	-29.8	9.6	43.0	13.5	3.70	1		C:N unacceptable
This study, BBM	IUBC 4372	56-620	Sand Lake	54.950	-128.970	BC	1956-08-18	Scale							-30.8	y	0.5	-29.8	9.3	42.8	13.6	3.66	1		C:N unacceptable
This study, BBM	IUBC 4393	60-23	Shuswap Lake	50.820	-119.000	BC	1960-03-00	Scale							-27.0	y	0.6	-25.9	7.5	45.6	14.5	3.66	1		C:N unacceptable
This study, BBM	IUBC 4400	56-607	Spectacle Lake	53.120	-121.230	BC	1956-08-06	Scale							-29.1	y	0.5	-28.1	9.1	42.7	13.9	3.58	1		
This study, BBM	IUBC 4401	56-607	Spectacle Lake	53.120	-121.230	BC	1956-08-06	Scale							-28.9	y	0.5	-27.9	9.3	42.6	14.1	3.51	1		
This study, BBM	IUBC 4402	56-607	Spectacle Lake	53.120	-121.230	BC	1956-08-06	Scale							-28.0	y	0.5	-27.0	9.8	42.3	14.4	3.42	1		
This study, RBCM	IUBC 3897	975-311-008, fish 1	Tezzeron Lake	54.700	-124.467	BC	1978-06-25	Scale							-29.5	y	0.8	-28.2	9.0	42.5	14.6	3.39	1		
This study, RBCM	IUBC 3898	975-311-008, fish 2	Tezzeron Lake	54.700	-124.467	BC	1975-06-28	Scale							-29.7	y	0.8	-28.3	9.1	41.0	14.3	3.35	1		
This study, RBCM	IUBC 3899	975-311-008, fish 3	Tezzeron Lake	54.700	-124.467	BC	1975-06-28	Scale							-29.9	y	0.8	-28.6	8.9	44.3	15.1	3.42	1		
This study, RBCM	IUBC 3900	975-311-008, fish 4	Tezzeron Lake	54.700	-124.467	BC	1975-06-28	Scale							-30.1	y	0.8	-28.8	9.2	44.2	15.3	3.38	1		
This study, RBCM	IUBC 3901	975-311-008, fish 5	Tezzeron Lake	54.700	-124.467	BC	1975-06-28	Scale							-30.5	y	0.8	-29.1	9.1	44.3	15.2	3.39	1		
This study, RBCM	IUBC 3902	975-311-008, fish 6	Tezzeron Lake	54.700	-124.467	BC	1975-06-28	Scale							-30.3	y	0.8	-29.0	9.1	39.1	13.3	3.43	1		
This study, RBCM	IUBC 3903	975-311-008, fish 7	Tezzeron Lake	54.700	-124.467	BC	1975-06-28	Scale							-30.3	y	0.8	-29.0	9.3	44.3	15.2	3.41	1		
This study, RBCM	IUBC 3904	975-311-008, fish 8	Tezzeron Lake	54.700	-124.467	BC	1975-06-28	Scale							-30.1	y	0.8	-28.7	9.4	44.1	15.4	3.34	1		
This study, RBCM	IUBC 3905	975-311-008, fish 9	Tezzeron Lake	54.700	-124.467	BC	1975-06-28	Scale							-30.8	y	0.8	-29.5	9.3	42.5	14.2	3.50	1		
This study, RBCM	IUBC 3906	975-311-008, fish 10	Tezzeron Lake	54.700	-124.467	BC	1975-06-28	Scale							-30.2	y	0.8	-28.9	9.2	44.4	15.4	3.37	1		
This study, RBCM	IUBC 3907	975-311-008, fish 11	Tezzeron Lake	54.700	-124.467	BC	1975-06-28	Scale							-30.1	y	0.8	-28.8	9.2	44.3	15.4	3.34	1		
This study, RBCM	IUBC 3908	975-311-008, fish 12	Tezzeron Lake	54.700	-124.467	BC	1975-06-28	Scale							-31.0	y	0.8	-29.7	9.4	45.2	14.9	3.54	1		
This study, RBCM	IUBC 3909	975-311-008, fish 13	Tezzeron Lake	54.700	-124.467	BC	1975-06-28	Scale							-30.1	y	0.8	-28.8	9.0	44.0	15.4	3.35	1		
This study, RBCM	IUBC 3910	975-311-008, fish 14	Tezzeron Lake	54.700	-124.467	BC	1975-06-28	Scale							-30.3	y	0.8	-29.0	9.0	45.2	15.5	3.41	1		
This study, RBCM	IUBC 3911	975-311-008, fish 15	Tezzeron Lake	54.700	-124.467	BC	1975-06-28	Scale							-31.0	y	0.8	-29.7	9.7	44.3	14.7	3.51	1		
This study, RBCM	IUBC 3912	975-311-008, fish 16	Tezzeron Lake	54.700	-124.467	BC	1975-06-28	Scale							-29.8	y	0.8	-28.5	9.1	43.3	15.1	3.35	1		
This study, RBCM	IUBC 3913	975-311-008, fish 17	Tezzeron Lake	54.700	-124.467	BC	1975-06-28	Scale							-31.2	y	0.8	-29.9	9.7	44.1	14.7	3.50	1		
This study, RBCM	IUBC 3914	975-311-008, fish 18	Tezzeron Lake	54.700	-124.467	BC	1975-06-28	Scale							-31.0	y	0.8	-29.7	9.4	45.1	14.6	3.60	1		
This study, RBCM	IUBC 3915	975-311-008, fish 19	Tezzeron Lake	54.700	-124.467	BC	1975-06-28	Scale							-31.3	y	0.8	-29.9	9.2	44.6	14.3	3.63	1		

Source/Donor	Lab No.	Cat No	Site Name	Latitude	Longitude	Prov. State	Capture Date	Material	Col. Yld. %	Mus. $\delta^{13}C$	Mus. %C	Mus. %N	Mus. C:N	Mus. $\delta^{13}C$, lipid cor.	Col. $\delta^{13}C$	Form. cor.	Suess cor. (‰)	$\delta^{13}C_{cor}$	$\delta^{15}N$	Col. %C	Col. %N	Col. C:N	*A priori* ecotype	$\delta^{13}C$ ecotype ID	Notes
This study, RBCM	IUBC 3916	975-311-008, fish 20	Tezzeron Lake	54.700	-124.467	BC	1975-06-28	Scale							-30.0	y	0.8	-28.6	9.4	44.4	15.2	3.40	1		
This study, RBCM	IUBC 3917	975-311-008, fish 21	Tezzeron Lake	54.700	-124.467	BC	1975-06-28	Scale							-29.7	y	0.8	-28.4	9.0	44.1	15.2	3.39	1		
This study, BBM	IUBC 4394	06-0105	Thutade Lake	56.780	-124.280	BC	1998-06-16	Scale							-28.6	y	1.5	-26.6	9.1	45.6	15.3	3.48	1		
This study, BBM	IUBC 4395	06-0105	Thutade Lake	56.780	-124.280	BC	1998-06-16	Scale							-28.9	y	1.5	-26.9	8.6	42.3	13.9	3.54	1		
This study, BBM	IUBC 4396	06-0105	Thutade Lake	56.780	-124.280	BC	1998-06-16	Scale							-28.3	y	1.5	-26.3	8.7	42.1	14.4	3.41	1		
This study, BBM	IUBC 4397	06-0105	Thutade Lake	56.780	-124.280	BC	1998-06-16	Scale							-28.9	y	1.5	-26.9	9.4	42.4	14.4	3.44	1		
This study, BBM	IUBC 4398	06-0105	Thutade Lake	56.780	-124.280	BC	1998-06-16	Scale							-28.3	y	1.5	-26.3	8.8	42.0	14.5	3.38	1		
This study, FLNR	IUBC 5325	5	Wahleach Lake	49.233	-121.612	BC	2013-08-10	Scale							-24.2	n	2.1	-22.1	6.3	47.3	17.7	3.11	1		
This study, FLNR	IUBC 5326	9	Wahleach Lake	49.233	-121.612	BC	2013-08-10	Scale							-24.0	n	2.1	-21.9	5.8	47.7	18.3	3.04	1		
This study, FLNR	IUBC 5327	10	Wahleach Lake	49.233	-121.612	BC	2013-08-10	Scale							-24.4	n	2.1	-22.3	6.3	43.8	16.6	3.09	1		
This study, FLNR	IUBC 5328	12	Wahleach Lake	49.233	-121.612	BC	2013-08-10	Scale							-24.2	n	2.1	-22.1	6.2	47.0	18.0	3.04	1		
This study, FLNR	IUBC 5329	16	Wahleach Lake	49.233	-121.612	BC	2013-08-10	Scale							-23.6	n	2.1	-21.5	6.2	47.1	17.7	3.09	1		
This study, FLNR	IUBC 5330	20	Wahleach Lake	49.233	-121.612	BC	2013-08-10	Scale							-24.2	n	2.1	-22.1	6.0	47.1	17.8	3.09	1		
This study, FLNR	IUBC 5331	23	Wahleach Lake	49.233	-121.612	BC	2013-08-10	Scale							-23.5	n	2.1	-21.4	6.1	47.0	17.5	3.13	1		
This study, FLNR	IUBC 5332	32	Wahleach Lake	49.233	-121.612	BC	2013-08-10	Scale							-23.8	n	2.1	-21.8	6.1	47.1	17.9	3.07	1		
This study, FLNR	IUBC 5333	40	Wahleach Lake	49.233	-121.612	BC	2013-08-10	Scale							-24.6	n	2.1	-22.6	6.1	47.2	17.7	3.11	1		
This study, FLNR	IUBC 5334	42	Wahleach Lake	49.233	-121.612	BC	2013-08-10	Scale							-23.8	n	2.1	-21.7	5.9	47.1	17.7	3.11	1		
This study, FLNR	IUBC 5335	43	Wahleach Lake	49.233	-121.612	BC	2013-08-10	Scale							-24.1	n	2.1	-22.1	6.0	47.5	18.0	3.08	1		
This study, FLNR	IUBC 5336	120	Wahleach Lake	49.233	-121.612	BC	2013-08-10	Scale							-25.2	n	2.1	-23.1	5.7	46.9	17.8	3.08	1		
This study, FLNR	IUBC 5337	121	Wahleach Lake	49.233	-121.612	BC	2013-08-10	Scale							-24.9	n	2.1	-22.9	6.3	47.4	17.9	3.09	1		
This study, FLNR	IUBC 5338	124	Wahleach Lake	49.233	-121.612	BC	2013-08-10	Scale							-24.0	n	2.1	-22.0	6.0	44.6	16.3	3.19	1		
This study, FLNR	IUBC 5339	126	Wahleach Lake	49.233	-121.612	BC	2013-08-10	Scale							-24.3	n	2.1	-22.2	5.2	41.6	15.6	3.11	1		
This study, BBM	IUBC 4373	55-272	Wilson Lake	50.230	-117.630	BC	1955-08-02	Scale							-27.5	y	0.5	-26.6	9.0	42.7	13.8	3.61	1		
This study, ROM	IUBC 4097	026551	Babine River	55.603	-127.160	BC	1969-02-00	Scale							-18.2	y	0.3	-17.4	10.8	42.1	14.5	3.39	2		
This study, ROM	IUBC 4098	026551	Babine River	55.603	-127.160	BC	1969-02-00	Scale							-18.6	y	0.3	-17.8	10.9	42.1	14.1	3.48	2		
This study, ROM	IUBC 4099	026551	Babine River	55.603	-127.160	BC	1969-02-00	Scale							-31.4	y	0.7	-30.2	8.5	43.0	12.6	3.98	2		C:N unacceptable
This study, ROM	IUBC 4212	026551	Babine River	55.603	-127.160	BC	1969-02-00	Scale							-18.1	y	0.3	-17.2	10.7	41.1	13.7	3.49	2		
This study, TCAR	IUBC 6965	1	Barkley Sound	48.899	-125.273	BC	2016-00-00	Scale							-17.2	n	1.2	-15.9	11.2	40.4	15.0	3.15	2		
This study, TCAR	IUBC 6966	2	Barkley Sound	48.899	-125.273	BC	2016-00-00	Scale							-17.3	n	1.2	-16.1	11.0	40.4	15.1	3.12	2		
This study, TCAR	IUBC 6967	3	Barkley Sound	48.899	-125.273	BC	2016-00-00	Scale							-18.3	n	1.2	-17.1	10.2	40.4	15.0	3.14	2		
This study, TCAR	IUBC 6968	4	Barkley Sound	48.899	-125.273	BC	2016-00-00	Scale							-18.0	n	1.2	-16.7	9.9	40.2	14.8	3.16	2		
This study, BBM	IUBC 4382	63-1045	Lake Sarannoe	55.303	166.076	RUS	1961-07-13	Scale							-17.9	y	0.3	-17.1	11.1	41.7	14.4	3.37	2		
This study, BBM	IUBC 4383	63-1046	Lake Sarannoe	55.303	166.076	RUS	1961-07-14	Scale							-16.8	y	0.3	-16.0	11.6	44.6	15.5	3.35	2		
This study, MHHP	TEAL 3912	6	Moricetown Camp Side	55.015	-127.330	BC	2009-00-00	Scale							-18.1	n	1.0	-17.0	11.7	42.4	15.2	3.26	2		
This study, MHHP	TEAL 3913	7	Moricetown Camp Side	55.015	-127.330	BC	2009-00-00	Scale							-18.3	n	1.0	-17.2	10.4	42.4	15.1	3.27	2		
This study, MHHP	TEAL 3914	8	Moricetown Camp Side	55.015	-127.330	BC	2009-00-00	Scale							-18.4	n	1.0	-17.3	11.0	41.6	14.8	3.28	2		
This study, MHHP	TEAL 3915	9	Moricetown Camp Side	55.015	-127.330	BC	2009-00-00	Scale							-18.2	n	1.0	-17.2	10.5	42.0	15.2	3.22	2		
This study, MHHP	TEAL 3916	10	Moricetown Camp Side	55.015	-127.330	BC	2009-00-00	Scale							-18.3	n	1.0	-17.3	11.6	42.5	15.3	3.25	2		
This study, MHHP	TEAL 3922	16	Moricetown Camp Side	55.015	-127.330	BC	2009-00-00	Scale							-18.6	n	1.0	-17.6	9.9	37.3	13.9	3.13	2		
This study, MHHP	TEAL 3923	17	Moricetown Camp Side	55.015	-127.330	BC	2009-00-00	Scale							-17.8	n	1.0	-16.8	11.5	42.6	15.0	3.31	2		
This study, MHHP	TEAL 3924	18	Moricetown Camp Side	55.015	-127.330	BC	2009-00-00	Scale							-17.8	n	1.0	-16.8	11.5	42.0	15.2	3.21	2		
This study, MHHP	TEAL 3925	19	Moricetown Camp Side	55.015	-127.330	BC	2009-00-00	Scale							-18.1	n	1.0	-17.1	11.2	42.0	15.1	3.24	2		
This study, MHHP	TEAL 3926	20	Moricetown Camp Side	55.015	-127.330	BC	2009-00-00	Scale							-17.9	n	1.0	-16.9	10.7	42.5	15.0	3.31	2		
This study, ROM	IUBC 4087	003188	Owikeno Lake	51.675	-126.899	BC	1926-07-10	Scale							-16.6	y	0.1	-16.0	14.0	42.0	13.7	3.57	2		
This study, BH	IUBC 6949	S15046	Rivers Inlet	51.674	-127.267	BC	2015-07-27	Scale							-18.0	n	1.2	-16.8	10.0	38.8	14.7	3.07	2		
This study, BH	IUBC 6950	S15054	Rivers Inlet	51.674	-127.267	BC	2015-08-11	Scale							-17.7	n	1.2	-16.5	10.5	39.3	15.0	3.05	2		
This study, BH	IUBC 6951	S15058	Rivers Inlet	51.674	-127.267	BC	2015-08-04	Scale							-18.5	n	1.2	-17.3	10.6	39.4	14.5	3.17	2		
This study, BH	IUBC 6952	S15062	Rivers Inlet	51.674	-127.267	BC	2015-08-11	Scale							-17.8	n	1.2	-16.6	10.5	39.4	15.0	3.06	2		
This study, BH	IUBC 6953	S15064	Rivers Inlet	51.674	-127.267	BC	2015-08-11	Scale							-17.9	n	1.2	-16.7	10.9	39.4	15.0	3.06	2		
This study, BH	IUBC 6954	S15066	Rivers Inlet	51.674	-127.267	BC	2015-08-04	Scale							-17.2	n	1.2	-16.0	10.6	17.4	12.8	1.53	2		C:N unacceptable

Source/Donor	Lab No.	Cat No	Site Name	Latitude	Longitude	Prov. State	Capture Date	Material	Col. Yld. %	Mus. $\delta^{13}C$	Mus. %C	Mus. %N	Mus. C:N	Mus. $\delta^{13}C$, lipid cor.	Col. $\delta^{13}C$	Form. cor.	Suess cor. (‰)	$\delta^{13}C_{cor}$	$\delta^{15}N$	Col. %C	Col. %N	Col. C:N	*A priori* ecotype	$\delta^{13}C$ ecotype ID	Notes
This study, BH	IUBC 6955	S15067	Rivers Inlet	51.674	-127.267	BC	2015-08-11	Scale							-18.7	n	1.2	-17.5	10.4	42.3	15.2	3.24	2		
This study, BH	IUBC 6956	S15080	Rivers Inlet	51.674	-127.267	BC	2015-08-04	Scale							-17.5	n	1.2	-16.3	10.5	41.6	15.8	3.07	2		
This study, BH	IUBC 6957	S15084	Rivers Inlet	51.674	-127.267	BC	2015-07-27	Scale							-17.8	n	1.2	-16.6	10.3	38.8	14.4	3.15	2		
This study, BH	IUBC 6958	S15087	Rivers Inlet	51.674	-127.267	BC	2015-07-30	Scale							-17.8	n	1.2	-16.6	11.4	35.8	13.0	3.21	2		
This study, BH	IUBC 6959	S15094	Rivers Inlet	51.674	-127.267	BC	2015-08-04	Scale							-17.6	n	1.2	-16.4	10.9	46.1	16.9	3.18	2		
This study, BH	IUBC 6960	S15095	Rivers Inlet	51.674	-127.267	BC	2015-08-04	Scale							-17.6	n	1.2	-16.4	10.5	41.7	15.5	3.14	2		
This study, BH	IUBC 6961	S15098	Rivers Inlet	51.674	-127.267	BC	2015-07-30	Scale							-18.3	n	1.2	-17.1	9.9	35.2	13.2	3.11	2		
This study, BH	IUBC 6962	S15099	Rivers Inlet	51.674	-127.267	BC	2015-07-27	Scale							-18.1	n	1.2	-16.9	10.7	39.1	14.6	3.13	2		
This study, BH	IUBC 6963	S15104	Rivers Inlet	51.674	-127.267	BC	2015-07-30	Scale							-19.2	n	1.2	-18.0	10.7	38.0	13.6	3.26	2		
This study, ROM	IUBC 4086	003250	Stamp Falls	49.334	-124.921	BC	1926-08-04	Scale							-16.4	y	0.1	-15.8	13.5	42.1	14.2	3.46	2		
This study, MHHP	TEAL 3907	1	Sustut Lake	56.573	-126.453	BC	2006-00-00	Scale							-18.2	n	1.3	-17.0	9.9	42.0	15.7	3.12	2		
This study, MHHP	TEAL 3908	2	Sustut Lake	56.573	-126.453	BC	2006-00-00	Scale							-18.2	n	1.3	-16.9	10.4	42.5	15.6	3.17	2		
This study, MHHP	TEAL 3909	3	Sustut Lake	56.573	-126.453	BC	2006-00-00	Scale							-17.8	n	1.3	-16.5	9.9	45.9	17.0	3.15	2		
This study, MHHP	TEAL 3910	4	Sustut Lake	56.573	-126.453	BC	2006-00-00	Scale							-17.3	n	1.3	-16.1	11.3	42.3	15.7	3.15	2		
This study, MHHP	TEAL 3911	5	Sustut Lake	56.573	-126.453	BC	2006-00-00	Scale							-17.8	n	1.3	-16.6	11.0	42.3	15.6	3.17	2		
This study, MHHP	TEAL 3917	11	Sustut Lake	56.573	-126.453	BC	2006-00-00	Scale							-16.9	n	1.3	-15.6	11.8	42.3	15.7	3.15	2		
This study, MHHP	TEAL 3918	12	Sustut Lake	56.573	-126.453	BC	2006-00-00	Scale							-17.8	n	1.3	-16.5	10.9	42.0	15.7	3.13	2		
This study, MHHP	TEAL 3919	13	Sustut Lake	56.573	-126.453	BC	2006-00-00	Scale							-18.1	n	1.3	-16.8	10.9	42.0	15.5	3.17	2		
This study, MHHP	TEAL 3921	15	Sustut Lake	56.573	-126.453	BC	2006-00-00	Scale							-18.4	n	1.3	-17.1	10.8	43.6	15.9	3.20	2		
This study, XFN	IUBC 5210	NA	Bear Lake (EkSa-36)	51.510	-124.236	BC	0000-00-00	Bone, vertebra	0.1							NA							3		Insufficient col. yld.
This study, XFN	IUBC 5213	NA	Bear Lake (EkSa-36)	51.510	-124.236	BC	0000-00-00	Bone, vertebra	0.1							NA							3		Insufficient col. yld.
This study, FLNR	TEAL 1710	1	Okanagan River	49.494	-119.617	BC	2018-10-09	Bone, vertebra	23.7						-23.8	n	2.3	-21.7	13.8	45.0	16.5	3.17	3	1	
This study, FLNR	TEAL 1711	2	Okanagan River	49.494	-119.617	BC	2018-10-09	Bone, vertebra	21.8						-23.0	n	2.3	-20.9	14.1	47.1	17.3	3.17	3	1	
This study, FLNR	TEAL 1712	6	Okanagan River	49.494	-119.617	BC	2018-10-09	Bone, vertebra	20.9						-22.8	n	2.3	-20.7	13.8	46.7	17.2	3.16	3	1	
This study, FLNR	TEAL 1713	11	Okanagan River	49.494	-119.617	BC	2018-10-09	Bone, vertebra	18.6						-23.8	n	2.3	-21.7	14.1	43.4	15.6	3.24	3	1	
This study, FLNR	TEAL 1714	15	Okanagan River	49.494	-119.617	BC	2018-10-09	Bone, vertebra	23.5						-17.0	n	1.3	-15.7	12.0	40.3	15.2	3.10	3	2	
This study, FLNR	TEAL 1715	19	Okanagan River	49.494	-119.617	BC	2018-10-09	Bone, vertebra	20.8						-23.2	n	2.3	-21.1	14.0	46.4	16.8	3.22	3	1	
This study, FLNR	TEAL 1716	21	Okanagan River	49.494	-119.617	BC	2018-10-09	Bone, vertebra	22.3						-18.4	n	1.3	-17.1	9.8	44.5	16.7	3.11	3	2	
This study, FLNR	TEAL 1717	28	Okanagan River	49.494	-119.617	BC	2018-10-19	Bone, vertebra	22.5						-18.8	n	1.3	-17.5	10.2	42.3	15.4	3.20	3	2	
This study, FLNR	TEAL 1718	50	Okanagan River	49.494	-119.617	BC	2018-10-19	Bone, vertebra	22.3						-17.8	n	1.3	-16.5	10.8	47.1	17.4	3.15	3	2	
This study, FLNR	TEAL 1719	54	Okanagan River	49.494	-119.617	BC	2018-10-19	Bone, vertebra	22.3						-17.9	n	1.3	-16.6	11.0	44.7	16.5	3.16	3	2	
This study, FLNR	TEAL 1720	62	Okanagan River	49.494	-119.617	BC	2018-10-19	Bone, vertebra	15.5						-17.6	n	1.3	-16.3	10.7	46.9	17.5	3.13	3	2	
This study, FLNR	TEAL 1721	76	Okanagan River	49.494	-119.617	BC	2018-10-19	Bone, vertebra	21.1						-18.2	n	1.3	-16.9	10.2	46.6	17.4	3.13	3	2	
This study, FLNR	TEAL 1722	89	Okanagan River	49.494	-119.617	BC	2018-10-19	Bone, vertebra	20.5						-18.0	n	1.3	-16.7	10.9	44.8	16.8	3.10	3	2	
This study, XFN	IUBC 5165	NA	Shields (EkSA-13)	51.510	-124.236	BC	0000-00-00	Bone, vertebra	0.4							NA							3		Insufficient col. yld.
This study, XFN	IUBC 5168	NA	Shields (EkSA-13)	51.510	-124.236	BC	0000-00-00	Bone, vertebra	0.3							NA							3		Insufficient col. yld.
This study, XFN	IUBC 5189	NA	Shields (EkSA-13)	51.510	-124.236	BC	0000-00-00	Bone, vertebra	0.1							NA							3		Insufficient col. yld.
This study, XFN	IUBC 5190	NA	Shields (EkSA-13)	51.510	-124.236	BC	0000-00-00	Bone, vertebra	0.2							NA							3		Insufficient col. yld.
This study, XFN	IUBC 5204	NA	Shields (EkSA-13)	51.510	-124.236	BC	0000-00-00	Bone, vertebra	0.2							NA							3		Insufficient col. yld.
This study, XFN	IUBC 5205	NA	Shields (EkSA-13)	51.510	-124.236	BC	0000-00-00	Bone, vertebra	0.2							NA							3		Insufficient col. yld.
This study, XFN	IUBC 5209	NA	Shields (EkSA-13)	51.510	-124.236	BC	0000-00-00	Bone, vertebra	0.2							NA							3		Insufficient col. yld.
This study, BBM	IUBC 4403	56-0455	Babine Lake	54.800	-126.130	BC	1956-07-17	Scale							-27.9	y	0.5	-26.9	7.8	42.4	14.0	3.52	4	1	
This study, BBM	IUBC 4404	56-0455	Babine Lake	54.800	-126.130	BC	1956-07-17	Scale							-27.7	y	0.5	-26.7	8.2	41.9	14.1	3.46	4	1	
This study, BBM	IUBC 4405	56-0455	Babine Lake	54.800	-126.130	BC	1956-07-17	Scale							-27.5	y	0.5	-26.5	7.9	34.8	11.8	3.44	4	1	
This study, ROM	IUBC 4089	004069	Columbia River	49.094	-117.698	BC	1926-10-09	Scale							-17.6	y	0.1	-17.0	12.4	41.6	13.9	3.48	4	2	
This study, ROM	IUBC 4088	004028	Eagle Lake	50.985	-118.630	BC	1926-09-07	Scale							-25.8	y	0.2	-25.1	7.7	41.6	14.1	3.45	4	1	
This study, ROM	IUBC 4067	006802	Harrison Lake	49.562	-121.862		1925-10-00	Scale							-25.5	y	0.2	-24.8	8.7	42.8	14.2	3.51	4	1	
This study, ROM	IUBC 4066	003327	Okanagan Lake	49.834	-119.524	BC	1926-07-10	Scale							-24.1	y	0.2	-23.3	6.7	43.3	14.3	3.54	4	1	
This study, BBM	IUBC 4380	56-538	Osoyoos Lake	49.022	-119.454	BC	1956-08-23	Scale							-18.0	y	0.2	-17.2	12.0	41.3	14.3	3.36	4	2	
This study, BBM	IUBC 4381	56-538	Osoyoos Lake	49.022	-119.454	BC	1956-08-23	Scale							-18.4	y	0.2	-17.6	10.7	41.3	14.5	3.31	4	2	

Source/Donor	Lab No.	Cat No	Site Name	Latitude	Longitude	Prov. State	Capture Date	Material	Col. Yld. %	Mus. $\delta^{13}C$	Mus. %C	Mus. %N	Mus. C:N	Mus. $\delta^{13}C$, lipid cor.	Col. $\delta^{13}C$	Form. cor.	Suess cor. (‰)	$\delta^{13}C_{cor}$	$\delta^{15}N$	Col. %C	Col. %N	Col. C:N	*A priori* ecotype	$\delta^{13}C$ ecotype ID	Notes
This study, BBM	IUBC 4399	57-196	Saomenos Creek	48.770	-123.670	BC	1957-00-00	Scale							-26.6	y	0.5	-25.6	7.1	41.6	14.9	3.25	4	1	
This study, ROM	IUBC 4068	007009	Shuswap Lake	50.820	-119.000	BC	1928-06-31	Scale							-26.0	y	0.2	-25.2	7.8	43.2	14.2	3.55	4	1	
This study, BBM	IUBC 4384	60-23	Shuswap Lake	50.820	-119.000	BC	1960-03-00	Scale							-27.3	y	0.2	-26.6	8.3	45.3	14.8	3.56	4	1	
This study, BBM	IUBC 4386	60-23	Shuswap Lake	50.820	-119.000	BC	1960-03-00	Scale							-27.1	y	0.2	-26.4	8.0	45.2	15.2	3.46	4	1	
This study, BBM	IUBC 4387	60-23	Shuswap Lake	50.820	-119.000	BC	1960-03-00	Scale							-26.9	y	0.2	-26.2	8.5	45.2	15.1	3.49	4	1	
This study, BBM	IUBC 4388	60-23	Shuswap Lake	50.820	-119.000	BC	1960-03-00	Scale							-26.9	y	0.2	-26.1	7.5	45.4	15.3	3.47	4	1	
This study, BBM	IUBC 4389	60-23	Shuswap Lake	50.820	-119.000	BC	1960-03-00	Scale							-26.9	y	0.2	-26.2	8.2	45.1	14.9	3.54	4	1	
This study, BBM	IUBC 4390	60-23	Shuswap Lake	50.820	-119.000	BC	1960-03-00	Scale							-26.9	y	0.2	-26.2	7.5	45.5	15.0	3.54	4	1	
This study, BBM	IUBC 4391	60-23	Shuswap Lake	50.820	-119.000	BC	1960-03-00	Scale							-27.0	y	0.2	-26.3	8.1	45.6	14.7	3.62	4	1	
This study, BBM	IUBC 4392	60-23	Shuswap Lake	50.820	-119.000	BC	1960-03-00	Scale							-26.8	y	0.2	-26.1	8.4	45.4	15.0	3.54	4	1	
This study, ROM	IUBC 4077	006609	Tum Tum Creek	51.881	-119.116	BC	1929-09-18	Scale							-27.0	y	0.2	-26.3	8.9	42.7	14.2	3.50	4	1	
This study, XFN	IUBC 5211	NA	Bear Lake (EkSa-36)	51.510	-124.236	BC	0000-00-00	Bone, vertebra	9.0						-16.1	n	0.0	-16.1	9.8	39.1	14.1	3.23	5	2	
This study, XFN	IUBC 5214	NA	Bear Lake (EkSa-36)	51.510	-124.236	BC	0000-00-00	Bone, vertebra	11.7						-15.6	n	0.0	-15.6	10.3	40.3	14.5	3.24	5	2	
This study, XFN	IUBC 5215	NA	Bear Lake (EkSa-36)	51.510	-124.236	BC	0000-00-00	Bone, vertebra	7.1						-16.0	n	0.0	-16.0	9.7	38.7	14.0	3.22	5	2	
This study, XFN	IUBC 5216	NA	Bear Lake (EkSa-36)	51.510	-124.236	BC	0000-00-00	Bone, vertebra	9.5						-16.3	n	0.0	-16.3	11.3	38.6	13.9	3.24	5	2	
This study, XFN	IUBC 5217	NA	Bear Lake (EkSa-36)	51.510	-124.236	BC	0000-00-00	Bone, vertebra	3.1						-16.2	n	0.0	-16.2	9.5	39.2	14.1	3.25	5	2	
This study, XFN	IUBC 5218	NA	Bear Lake (EkSa-36)	51.510	-124.236	BC	0000-00-00	Bone, vertebra	11.7						-16.1	n	0.0	-16.0	11.5	39.6	14.5	3.17	5	2	
This study, XFN	IUBC 5219	NA	Bear Lake (EkSa-36)	51.510	-124.236	BC	0000-00-00	Bone, vertebra	5.6						-16.2	n	0.0	-16.2	11.1	39.5	14.3	3.23	5	2	
This study, XFN	IUBC 5220	NA	Bear Lake (EkSa-36)	51.510	-124.236	BC	0000-00-00	Bone, vertebra	10.6						-15.7	n	0.0	-15.7	10.2	39.8	14.7	3.17	5	2	
This study, XFN	IUBC 5221	NA	Bear Lake (EkSa-36)	51.510	-124.236	BC	0000-00-00	Bone, vertebra	9.4						-15.9	n	0.0	-15.9	10.5	39.1	14.2	3.21	5	2	
This study, XFN	IUBC 5222	NA	Bear Lake (EkSa-36)	51.510	-124.236	BC	0000-00-00	Bone, vertebra	13.5						-15.8	n	0.0	-15.8	9.4	40.9	15.0	3.17	5	2	
This study, XFN	IUBC 5223	NA	Bear Lake (EkSa-36)	51.510	-124.236	BC	0000-00-00	Bone, vertebra	9.3						-16.0	n	0.0	-16.0	10.4	39.6	14.2	3.25	5	2	
This study, XFN	IUBC 5224	NA	Bear Lake (EkSa-36)	51.510	-124.236	BC	0000-00-00	Bone, vertebra	9.7						-16.3	n	0.0	-16.3	10.3	40.3	14.7	3.21	5	2	
This study, XFN	IUBC 5225	NA	Bear Lake (EkSa-36)	51.510	-124.236	BC	0000-00-00	Bone, vertebra	9.3						-15.6	n	0.0	-15.6	11.7	40.2	14.8	3.18	5	2	
This study, XFN	IUBC 5226	NA	Bear Lake (EkSa-36)	51.510	-124.236	BC	0000-00-00	Bone, vertebra	7.4						-16.1	n	0.0	-16.1	10.8	40.1	14.4	3.25	5	2	
This study, XFN	IUBC 5227	NA	Bear Lake (EkSa-36)	51.510	-124.236	BC	0000-00-00	Bone, vertebra	11.2						-16.1	n	0.0	-16.1	10.8	39.9	14.2	3.28	5	2	
This study, XFN	IUBC 5228	NA	Bear Lake (EkSa-36)	51.510	-124.236	BC	0000-00-00	Bone, vertebra	5.6						-16.9	n	0.0	-16.8	10.6	37.0	12.8	3.37	5	2	
This study, XFN	IUBC 5229	NA	Bear Lake (EkSa-36)	51.510	-124.236	BC	0000-00-00	Bone, vertebra	6.3						-17.0	n	0.0	-17.0	9.3	39.5	14.0	3.28	5	2	
This study, XFN	IUBC 5161	NA	Shields (EkSA-13)	51.510	-124.236	BC	0000-00-00	Bone, vertebra	4.8						-15.2	n	0.0	-15.2	12.4	37.8	13.7	3.23	5	2	
This study, XFN	IUBC 5166	NA	Shields (EkSA-13)	51.510	-124.236	BC	0000-00-00	Bone, vertebra	15.0						-16.2	n	0.0	-16.2	9.0	41.4	15.1	3.20	5	2	
This study, XFN	IUBC 5167	NA	Shields (EkSA-13)	51.510	-124.236	BC	0000-00-00	Bone, vertebra	2.6						-15.9	n	0.0	-15.9	10.5	40.5	14.6	3.24	5	2	
This study, XFN	IUBC 5182	NA	Shields (EkSA-13)	51.510	-124.236	BC	0000-00-00	Bone, vertebra	7.0						-15.8	n	0.0	-15.8	10.1	41.4	15.1	3.19	5	2	
This study, XFN	IUBC 5183	NA	Shields (EkSA-13)	51.510	-124.236	BC	0000-00-00	Bone, vertebra	4.9						-16.5	n	0.0	-16.5	9.3	40.6	14.5	3.26	5	2	
This study, XFN	IUBC 5206	NA	Shields (EkSA-13)	51.510	-124.236	BC	0000-00-00	Bone, vertebra	4.7						-16.3	n	0.0	-16.3	9.6	37.7	13.5	3.27	5	2	
This study, XFN	IUBC 5207	NA	Shields (EkSA-13)	51.510	-124.236	BC	0000-00-00	Bone, vertebra	9.0						-13.6	n	0.0	-16.2	14.8	41.2	14.9	3.20	5	NA	O. kistuch
This study, XFN	IUBC 5208	NA	Shields (EkSA-13)	51.510	-124.236	BC	0000-00-00	Bone, vertebra	2.4						-16.2	n	0.0	-16.2	9.8	40.1	14.0	3.35	5	2	
Satterfield and Finney 2002		EGEGIK 13	Beacharof Lake	57.500	-156.070	AK	1997-00-00	Scale							-21.3	n	1.4	-19.9	8.1	42.0	16.0	3.07	1		Smolt
Satterfield and Finney 2002		EGEGIK 7	Beacharof Lake	57.500	-156.070	AK	1997-00-00	Scale							-21.6	n	1.4	-20.2	7.5	41.1	15.0	3.20	1		Smolt
Satterfield and Finney 2002		EGEGIK 4	Beacharof Lake	57.500	-156.070	AK	1997-00-00	Scale							-21.7	n	1.4	-20.2	7.5	41.3	15.0	3.21	1		Smolt
Satterfield and Finney 2002		EGEGIK 9	Beacharof Lake	57.500	-156.070	AK	1997-00-00	Scale							-22.4	n	1.4	-20.9	7.8	26.9	9.5	3.31	1		Smolt
Satterfield and Finney 2002		EGEGIK 15	Beacharof Lake	57.500	-156.070	AK	1997-00-00	Scale							-21.7	n	1.4	-20.3	8.1	36.4	12.6	3.38	1		Smolt
Satterfield and Finney 2002		CHIGNIK 12	Chignik Lake	56.140	-158.490	AK	1997-00-00	Scale							-24.1	n	1.4	-22.6	8.9	43.8	16.4	3.12	1		Smolt
Satterfield and Finney 2002		CHIGNIK 17	Chignik Lake	56.140	-158.490	AK	1997-00-00	Scale							-24.1	n	1.4	-22.7	8.7	36.4	13.4	3.17	1		Smolt
Satterfield and Finney 2002		CHIGNIK 7	Chignik Lake	56.140	-158.490	AK	1997-00-00	Scale							-24.9	n	1.4	-23.5	8.8	48.7	17.8	3.20	1		Smolt
Satterfield and Finney 2002		CHIGNIK 15	Chignik Lake	56.140	-158.490	AK	1997-00-00	Scale							-24.1	n	1.4	-22.7	8.9	34.9	12.4	3.30	1		Smolt
Satterfield and Finney 2002		CHIGNIK 20	Chignik Lake	56.140	-158.490	AK	1997-00-00	Scale							-23.9	n	1.4	-22.5	9.1	41.0	13.7	3.49	1		Smolt
Satterfield and Finney 2002		COGHILL-SC-97-4	Coghill Lake	61.040	-147.290	AK	1997-00-00	Scale							-31.6	n	1.4	-30.2	7.3	33.0	13.7	2.82	1		C:N unacceptable, Smolt
Satterfield and Finney 2002		COGHILL-SC-97-2	Coghill Lake	61.040	-147.290	AK	1997-00-00	Scale							-30.2	n	1.4	-28.8	8.3	32.7	12.9	2.96	1		Smolt
Satterfield and Finney 2002		COGHILL-SC-97-3	Coghill Lake	61.040	-147.290	AK	1997-00-00	Scale							-31.3	n	1.4	-29.9	7.7	33.3	12.2	3.20	1		Smolt
Satterfield and Finney 2002		COGHILL-SC-97-5	Coghill Lake	61.040	-147.290	AK	1997-00-00	Scale							-29.2	n	1.4	-27.8	6.9				1		Smolt

Source/Donor	Lab No.	Cat No	Site Name	Latitude	Longitude	Prov. State	Capture Date	Material	Col. Yld. %	Mus. $\delta^{13}C$	Mus. %C	Mus. %N	Mus. C:N	Mus. $\delta^{13}C$, lipid cor.	Col. $\delta^{13}C$	Form. cor.	Suess cor. (‰)	$\delta^{13}C_{cor}$	$\delta^{15}N$	Col. %C	Col. %N	Col. C:N	*A priori* ecotype	$\delta^{13}C$ ecotype ID	Notes
Meeuwig and Peacock 2017		NA	Fallen Leaf Lake	38.903	-120.062	CA	2009-00-00	Muscle						-26.3	*-22.6	n	1.9	-20.7	4.8				1		
Meeuwig and Peacock 2017		NA	Fallen Leaf Lake	38.903	-120.062	CA	2009-00-00	Muscle						-26.3	*-22.6	n	1.9	-20.7	5.4				1		
Meeuwig and Peacock 2017		NA	Fallen Leaf Lake	38.903	-120.062	CA	2009-00-00	Muscle						-26.2	*-22.5	n	1.9	-20.6	4.8				1		
Meeuwig and Peacock 2017		NA	Fallen Leaf Lake	38.903	-120.062	CA	2009-00-00	Muscle						-26.2	*-22.5	n	1.9	-20.6	4.7				1		
Meeuwig and Peacock 2017		NA	Fallen Leaf Lake	38.903	-120.062	CA	2009-00-00	Muscle						-26.2	*-22.5	n	1.9	-20.6	5.1				1		
Meeuwig and Peacock 2017		NA	Fallen Leaf Lake	38.903	-120.062	CA	2009-00-00	Muscle						-26.1	*-22.4	n	1.9	-20.5	5.0				1		
Meeuwig and Peacock 2017		NA	Fallen Leaf Lake	38.903	-120.062	CA	2009-00-00	Muscle						-26.1	*-22.4	n	1.9	-20.5	4.9				1		
Meeuwig and Peacock 2017		NA	Fallen Leaf Lake	38.903	-120.062	CA	2009-00-00	Muscle						-25.9	*-22.2	n	1.9	-20.3	4.1				1		
Satterfield and Finney 2002		HIDDEN 5	Hidden Lake	58.230	-152.420	AK	1997-00-00	Scale							-27.1	n	1.4	-25.7	6.3	45.6	18.4	2.90	1		C:N unacceptable, Smolt
Satterfield and Finney 2002		HIDDEN 1	Hidden Lake	58.230	-152.420	AK	1997-00-00	Scale							-27.2	n	1.4	-25.7	5.2	29.0	11.6	2.92	1		Smolt
Satterfield and Finney 2002		HIDDEN 2	Hidden Lake	58.230	-152.420	AK	1997-00-00	Scale							-26.8	n	1.4	-25.4	6.3	39.9	15.9	2.92	1		Smolt
Satterfield and Finney 2002		HIDDEN 4	Hidden Lake	58.230	-152.420	AK	1997-00-00	Scale							-27.3	n	1.4	-25.8	5.2	35.4	14.1	2.93	1		Smolt
Satterfield and Finney 2002		KARLUK-SC-22-S.S	Karluk Lake	57.250	-154.050	AK	1997-00-00	Scale							-21.2	n	1.4	-19.8	16.2	26.6	9.2	3.37	1		Smolt
Overman et al. 2009		NA	Lake Sammamish	47.609	-122.089	WA	2003-01-01	Muscle						-33.8	*-30.1	n	1.9	-28.2	15.1				1		
Overman et al. 2009		NA	Lake Sammamish	47.609	-122.089	WA	2003-01-01	Muscle						-33.3	*-29.6	n	1.9	-27.7	15.2				1		
Overman et al. 2009		NA	Lake Sammamish	47.609	-122.089	WA	2003-01-01	Muscle						-33.2	*-29.6	n	1.9	-27.7	15.1				1		
Overman et al. 2009		NA	Lake Sammamish	47.609	-122.089	WA	2003-01-01	Muscle						-32.9	*-29.2	n	1.9	-27.3	15.1				1		
Overman et al. 2009		NA	Lake Sammamish	47.609	-122.089	WA	2003-01-01	Muscle						-31.9	*-28.2	n	1.9	-26.3	15.1				1		
Overman et al. 2009		NA	Lake Sammamish	47.609	-122.089	WA	2003-01-01	Muscle						-31.2	*-27.5	n	1.9	-25.6	15.3				1		
Overman et al. 2009		NA	Lake Sammamish	47.609	-122.089	WA	2003-01-01	Muscle						-30.1	*-26.4	n	1.9	-24.5	15.1				1		
Overman et al. 2009		NA	Lake Sammamish	47.609	-122.089	WA	2003-01-01	Muscle						-29.8	*-26.1	n	1.9	-24.2	16.0				1		
Overman et al. 2009		NA	Lake Sammamish	47.609	-122.089	WA	2003-01-01	Muscle						-29.5	*-25.8	n	1.9	-23.9	15.0				1		
Overman et al. 2009		NA	Lake Sammamish	47.609	-122.089	WA	2003-01-01	Muscle						-29.5	*-25.8	n	1.9	-23.9	16.2				1		
Overman et al. 2009		NA	Lake Sammamish	47.609	-122.089	WA	2003-01-01	Muscle						-29.5	*-25.8	n	1.9	-23.9	15.0				1		
Overman et al. 2009		NA	Lake Sammamish	47.609	-122.089	WA	2003-01-01	Muscle						-29.3	*-25.6	n	1.9	-23.7	16.1				1		
Overman et al. 2009		NA	Lake Sammamish	47.609	-122.089	WA	2003-01-01	Muscle						-29.1	*-25.5	n	1.9	-23.6	14.1				1		
Overman et al. 2009		NA	Lake Sammamish	47.609	-122.089	WA	2003-01-01	Muscle						-28.0	*-24.3	n	1.9	-22.4	15.3				1		
Overman et al. 2009		NA	Lake Sammamish	47.609	-122.089	WA	2003-01-01	Muscle						-28.0	*-24.3	n	1.9	-22.4	14.7				1		
Overman et al. 2009		NA	Lake Sammamish	47.609	-122.089	WA	2003-01-01	Muscle						-27.7	*-24.0	n	1.9	-22.1	13.4				1		
Overman et al. 2009		NA	Lake Sammamish	47.609	-122.089	WA	2003-01-01	Muscle						-27.5	*-23.9	n	1.9	-22.0	13.9				1		
Satterfield and Finney 2002		LAURA-SC-5-S.S	Laura Lake	58.380	-152.300	AK	1997-00-00	Scale							-15.4	n	1.4	-14.0	15.0	24.3	8.6	3.29	1		Smolt
Satterfield and Finney 2002		LAURA-SC-10-S.S	Laura Lake	58.380	-152.300	AK	1997-00-00	Scale							-24.8	n	1.4	-23.3	10.9	26.3	9.2	3.32	1		Smolt
Satterfield and Finney 2002		SPIR-SC-12-S.S	Spiridon Lake	57.410	-153.290	AK	1997-00-00	Scale							-25.5	n	1.4	-24.0	10.5	29.2	10.4	3.26	1		Smolt
Satterfield and Finney 2002		SPIR-SC-1-S.S	Spiridon Lake	57.410	-153.290	AK	1997-00-00	Scale							-24.8	n	1.4	-23.4	10.0	28.3	10.1	3.27	1		Smolt
B. Finney Unpublished		WEN-SC-8	Wenatchee Lake	47.826	-120.776	WA	1997-00-00	Scale							-26.5	n	1.4	-25.1	7.6	25.7	10.2	2.93	1		Smolt
B. Finney Unpublished		WEN-SC-4	Wenatchee Lake	47.826	-120.776	WA	1997-00-00	Scale							-27.4	n	1.4	-26.0	6.5	25.8	10.3	2.94	1		Smolt
B. Finney Unpublished		WEN-SC-10	Wenatchee Lake	47.826	-120.776	WA	1997-00-00	Scale							-25.6	n	1.4	-24.2	7.0	24.3	9.3	3.03	1		Smolt
B. Finney Unpublished		WEN-SC-11	Wenatchee Lake	47.826	-120.776	WA	1997-00-00	Scale							-26.2	n	1.4	-24.7	7.6	25.1	9.5	3.10	1		Smolt
B. Finney Unpublished		WEN-SC-14	Wenatchee Lake	47.826	-120.776	WA	1997-00-00	Scale							-26.4	n	1.4	-24.9	7.3	23.2	8.6	3.13	1		Smolt
Welch and Parsons 1993		NA	Adams Lake	51.180	-119.574	BC	1991-00-00	Muscle						-20.9	*-17.2	n	0.6	-16.6	11.6				2		
Welch and Parsons 1993		NA	Adams Lake	51.180	-119.574	BC	1991-00-00	Muscle						-20.6	*-16.9	n	0.6	-16.3	11.1				2		
Welch and Parsons 1993		NA	Adams Lake	51.180	-119.574	BC	1991-00-00	Muscle						-20.5	*-16.8	n	0.6	-16.2	11.5				2		
Welch and Parsons 1993		NA	Adams Lake	51.180	-119.574	BC	1991-00-00	Muscle						-20.5	*-16.8	n	0.6	-16.2	11.9				2		
Welch and Parsons 1993		NA	Adams Lake	51.180	-119.574	BC	1991-00-00	Muscle						-20.0	*-16.3	n	0.6	-15.7	11.5				2		
Welch and Parsons 1993		NA	Adams Lake	51.180	-119.574	BC	1991-00-00	Muscle						-20.0	*-16.3	n	0.6	-15.7	11.9				2		
Welch and Parsons 1993		NA	Adams Lake	51.180	-119.574	BC	1991-00-00	Muscle						-20.0	*-16.3	n	0.6	-15.7	11.1				2		
Welch and Parsons 1993		NA	Adams Lake	51.180	-119.574	BC	1991-00-00	Muscle						-19.9	*-16.2	n	0.6	-15.6	12.4				2		
Welch and Parsons 1993		NA	Adams Lake	51.180	-119.574	BC	1991-00-00	Muscle						-19.8	*-16.1	n	0.6	-15.5	12.3				2		
Welch and Parsons 1993		NA	Adams Lake	51.180	-119.574	BC	1991-00-00	Muscle						-19.8	*-16.1	n	0.6	-15.5	11.9				2		
Welch and Parsons 1993		NA	Adams Lake	51.180	-119.574	BC	1991-00-00	Muscle						-19.6	*-15.9	n	0.6	-15.3	11.8				2		

Source/Donor	Lab No.	Cat No	Site Name	Latitude	Longitude	Prov. State	Capture Date	Material	Col. Yld. %	Mus. $\delta^{13}C$	Mus. %C	Mus. %N	Mus. C:N	Mus. $\delta^{13}C$, lipid cor.	Col. $\delta^{13}C$	Form. cor.	Suess cor. (‰)	$\delta^{13}C_{cor}$	$\delta^{15}N$	Col. %C	Col. %N	Col. C:N	*A priori* ecotype	$\delta^{13}C$ ecotype ID	Notes
Welch and Parsons 1993		NA	Adams Lake	51.180	-119.574	BC	1991-00-00	Muscle						-19.3	*-15.6	n	0.6	-15.0	12.2				2		
Molkentin et al. 2015		58/11	Alaska Pacific Region	Unknown	Unknown		2011-00-00	Muscle						-21.1	*-17.4	n	1.1	-16.3	10.5				2		
Molkentin et al. 2015		39/10	Alaska Pacific Region	Unknown	Unknown		2011-00-00	Muscle						-21.0	*-17.3	n	1.1	-16.2	10.8				2		
Molkentin et al. 2015		40/10	Alaska Pacific Region	Unknown	Unknown		2011-00-00	Muscle						-20.9	*-17.2	n	1.1	-16.1	11.0				2		
Molkentin et al. 2015		57/11	Alaska Pacific Region	Unknown	Unknown		2011-00-00	Muscle						-20.8	*-17.1	n	1.1	-16.0	10.9				2		
Molkentin et al. 2015		38/10	Alaska Pacific Region	Unknown	Unknown		2011-00-00	Muscle						-20.6	*-17.0	n	1.1	-15.9	11.0				2		
Molkentin et al. 2015		02/12	Alaska Pacific Region	Unknown	Unknown		2011-00-00	Muscle						-20.6	*-16.9	n	1.1	-15.8	11.1				2		
Molkentin et al. 2015		115/11	Alaska Pacific Region	Unknown	Unknown		2011-00-00	Muscle						-20.6	*-16.9	n	1.1	-15.8	10.7				2		
Molkentin et al. 2015		206/11	Alaska Pacific Region	Unknown	Unknown		2011-00-00	Muscle						-20.6	*-16.9	n	1.1	-15.8	11.0				2		
Molkentin et al. 2015		02/11	Alaska Pacific Region	Unknown	Unknown		2011-00-00	Muscle						-20.5	*-16.8	n	1.1	-15.7	10.8				2		
Molkentin et al. 2015		71/11	Alaska Pacific Region	Unknown	Unknown		2011-00-00	Muscle						-20.4	*-16.8	n	1.1	-15.7	11.2				2		
Molkentin et al. 2015		69/11	Alaska Pacific Region	Unknown	Unknown		2011-00-00	Muscle						-20.4	*-16.7	n	1.1	-15.7	10.6				2		
Molkentin et al. 2015		60/11	Alaska Pacific Region	Unknown	Unknown		2011-00-00	Muscle						-20.4	*-16.7	n	1.1	-15.6	11.0				2		
Molkentin et al. 2015		153/11	Alaska Pacific Region	Unknown	Unknown		2011-00-00	Muscle						-20.2	*-16.5	n	1.1	-15.4	10.4				2		
Molkentin et al. 2015		164/11	Alaska Pacific Region	Unknown	Unknown		2011-00-00	Muscle						-20.1	*-16.4	n	1.1	-15.3	11.2				2		
Molkentin et al. 2015		36/10	Alaska Pacific Region	Unknown	Unknown		2011-00-00	Muscle						-20.1	*-16.4	n	1.1	-15.3	11.9				2		
Molkentin et al. 2015		56/11	Alaska Pacific Region	Unknown	Unknown		2011-00-00	Muscle						-20.1	*-16.4	n	1.1	-15.3	11.1				2		
Molkentin et al. 2015		205/11	Alaska Pacific Region	Unknown	Unknown		2011-00-00	Muscle						-20.0	*-16.3	n	1.1	-15.2	10.8				2		
Molkentin et al. 2015		59/11	Alaska Pacific Region	Unknown	Unknown		2011-00-00	Muscle						-20.0	*-16.3	n	1.1	-15.2	11.4				2		
Molkentin et al. 2015		03/12	Alaska Pacific Region	Unknown	Unknown		2011-00-00	Muscle						-19.8	*-16.1	n	1.1	-15.1	11.4				2		
Molkentin et al. 2015		01/11	Alaska Pacific Region	Unknown	Unknown		2011-00-00	Muscle						-19.8	*-16.1	n	1.1	-15.1	10.7				2		
Qin and Kaeriyama 2016		26	Aleutian Islands Waters	47.000	174.010		2009-00-00	Muscle			46.7	14.9	3.6	-20.7	*-17.0	n	1.0	-16.0	11.4				2		
Qin and Kaeriyama 2016		30	Aleutian Islands Waters	47.000	174.010		2009-00-00	Muscle			45.8	14.6	3.7	-20.7	*-17.0	n	1.0	-16.0	10.9				2		
Qin and Kaeriyama 2016		28	Aleutian Islands Waters	47.000	174.010		2009-00-00	Muscle			48.2	14.9	3.8	-20.6	*-16.9	n	1.0	-15.9	11.9				2		
Qin and Kaeriyama 2016		31	Aleutian Islands Waters	47.000	174.010		2009-00-00	Muscle			46.0	15.0	3.6	-20.1	*-16.4	n	1.0	-15.3	12.7				2		
Qin and Kaeriyama 2016		29	Aleutian Islands Waters	47.000	174.010		2009-00-00	Muscle			43.4	14.7	3.5	-19.9	*-16.3	n	1.0	-15.2	11.2				2		
Qin and Kaeriyama 2016		33	Aleutian Islands Waters	47.000	174.010		2009-00-00	Muscle			42.2	13.6	3.6	-19.7	*-16.0	n	1.0	-15.0	11.6				2		
Qin and Kaeriyama 2016		27	Aleutian Islands Waters	47.000	174.010		2009-00-00	Muscle			46.8	15.6	3.5	-19.5	*-15.8	n	1.0	-14.8	11.9				2		
Qin and Kaeriyama 2016		32	Aleutian Islands Waters	47.000	174.010		2009-00-00	Muscle			45.5	14.6	3.6	-19.0	*-15.3	n	1.0	-14.3	12.3				2		
Qin and Kaeriyama 2016		146	Aleutian Islands Waters	50.500	-179.440		2010-00-00	Muscle			48.4	15.7	3.6	-22.1	*-18.4	n	1.1	-17.4	10.7				2		
Qin and Kaeriyama 2016		174	Aleutian Islands Waters	51.410	-176.420		2010-00-00	Muscle			51.1	16.2	3.7	-21.6	*-17.9	n	1.1	-16.9	10.8				2		
Qin and Kaeriyama 2016		172	Aleutian Islands Waters	51.410	-176.420		2010-00-00	Muscle			48.9	15.8	3.6	-21.4	*-17.7	n	1.1	-16.6	10.6				2		
Qin and Kaeriyama 2016		142	Aleutian Islands Waters	50.500	-179.440		2010-00-00	Muscle			47.1	14.6	3.8	-21.1	*-17.4	n	1.1	-16.4	10.7				2		
Qin and Kaeriyama 2016		119	Aleutian Islands Waters	49.040	-173.550		2010-00-00	Muscle			48.0	15.9	3.5	-21.1	*-17.4	n	1.1	-16.4	10.4				2		
Qin and Kaeriyama 2016		143	Aleutian Islands Waters	50.500	-179.440		2010-00-00	Muscle			29.2	9.2	3.7	-21.1	*-17.4	n	1.1	-16.3	10.3				2		
Qin and Kaeriyama 2016		122	Aleutian Islands Waters	50.020	-165.000		2010-00-00	Muscle			49.3	15.7	3.7	-21.0	*-17.3	n	1.1	-16.2	10.8				2		
Qin and Kaeriyama 2016		144	Aleutian Islands Waters	50.500	-179.440		2010-00-00	Muscle			42.6	14.0	3.5	-20.9	*-17.2	n	1.1	-16.2	11.4				2		
Qin and Kaeriyama 2016		173	Aleutian Islands Waters	51.410	-176.420		2010-00-00	Muscle			45.5	14.6	3.6	-20.6	*-16.9	n	1.1	-15.8	11.0				2		
Qin and Kaeriyama 2016		120	Aleutian Islands Waters	50.020	-165.000		2010-00-00	Muscle			49.4	15.4	3.7	-20.6	*-16.9	n	1.1	-15.8	11.0				2		
Qin and Kaeriyama 2016		175	Aleutian Islands Waters	51.410	-176.420		2010-00-00	Muscle			46.0	15.5	3.5	-20.4	*-16.7	n	1.1	-15.7	11.4				2		
Qin and Kaeriyama 2016		165	Aleutian Islands Waters	51.090	-179.430		2010-00-00	Muscle			48.8	15.8	3.6	-20.3	*-16.6	n	1.1	-15.5	11.8				2		
Qin and Kaeriyama 2016		145	Aleutian Islands Waters	50.500	-179.440		2010-00-00	Muscle			50.1	15.7	3.7	-20.3	*-16.6	n	1.1	-15.5	11.4				2		
Qin and Kaeriyama 2016		176	Aleutian Islands Waters	51.410	-176.420		2010-00-00	Muscle			52.6	16.3	3.8	-19.8	*-16.1	n	1.1	-15.1	11.8				2		
Satterfield and Finney 2002		AUKE-RS12	Auke Lake	58.380	-134.390	AK	1997-00-00	Muscle		-20.6	48.5	15.0	3.2	-21.7	*-18.0	n	0.7	-17.2	10.5				2		
Satterfield and Finney 2002		AUKE-RS18	Auke Lake	58.380	-134.390	AK	1997-00-00	Muscle		-21.2	48.9	15.0	3.3	-22.2	*-18.5	n	0.7	-17.7	11.0				2		
Satterfield and Finney 2002		AUKE-RS5	Auke Lake	58.380	-134.390	AK	1997-00-00	Muscle		-21.1	48.4	14.7	3.3	-22.1	*-18.4	n	0.7	-17.7	10.6				2		
Satterfield and Finney 2002		BECH-10-97	Beacharof Lake	57.500	-156.070	AK	1997-00-00	Muscle		-20.4	52.5	15.8	3.3	-21.3	*-17.7	n	0.7	-16.9	10.4				2		
Satterfield and Finney 2002		BECH-5-97	Beacharof Lake	57.500	-156.070	AK	1997-00-00	Muscle		-20.4	54.1	16.3	3.3	-21.3	*-17.6	n	0.7	-16.9	11.3				2		
Satterfield and Finney 2002		BECH-8-97	Beacharof Lake	57.500	-156.070	AK	1997-00-00	Muscle		-20.4	59.4	17.8	3.3	-21.3	*-17.6	n	0.7	-16.9	11.0				2		
Satterfield and Finney 2002		BECH-4-97	Beacharof Lake	57.500	-156.070	AK	1997-00-00	Muscle		-20.1	52.1	15.3	3.4	-20.9	*-17.2	n	0.7	-16.5	11.0				2		

Source/Donor	Lab No.	Cat No	Site Name	Latitude	Longitude	Prov. State	Capture Date	Material	Col. Yld. %	Mus. $\delta^{13}C$	Mus. %C	Mus. %N	Mus. C:N	Mus. $\delta^{13}C$, lipid cor.	Col. $\delta^{13}C$	Form. cor.	Suess cor. (‰)	$\delta^{13}C_{cor}$	$\delta^{15}N$	Col. %C	Col. %N	Col. C:N	*A priori* ecotype	$\delta^{13}C$ ecotype ID	Notes
Satterfield and Finney 2002		BECH-9-97	Beacharof Lake	57.500	-156.070	AK	1997-00-00	Muscle		-20.2	52.4	15.4	3.4	-21.0	*-17.3	n	0.7	-16.6	10.9				2		
Satterfield and Finney 2002		BECH-603-98	Beacharof Lake	57.500	-156.070	AK	1998-00-00	Muscle		-20.2	48.1	14.1	3.4	-20.9	*-17.3	n	0.8	-16.5	11.3				2		
Satterfield and Finney 2002		BECH-609-98	Beacharof Lake	57.500	-156.070	AK	1998-00-00	Muscle		-20.4	48.7	14.2	3.4	-21.2	*-17.5	n	0.8	-16.8	11.4				2		
Satterfield and Finney 2002		BECH-695-98	Beacharof Lake	57.500	-156.070	AK	1998-00-00	Muscle		-20.5	49.4	14.4	3.4	-21.3	*-17.6	n	0.8	-16.8	11.2				2		
Satterfield and Finney 2002		BECH-680-98	Beacharof Lake	57.500	-156.070	AK	1998-00-00	Muscle		-21.0	49.4	14.4	3.4	-21.8	*-18.1	n	0.8	-17.3	11.3				2		
Satterfield and Finney 2002		BECH-611-98	Beacharof Lake	57.500	-156.070	AK	1998-00-00	Muscle		-20.4	48.7	14.1	3.4	-21.2	*-17.5	n	0.8	-16.7	11.3				2		
Satterfield and Finney 2002		BECH-675-98	Beacharof Lake	57.500	-156.070	AK	1998-00-00	Muscle		-20.8	48.5	14.0	3.5	-21.5	*-17.9	n	0.8	-17.1	11.3				2		
Satterfield and Finney 2002		BECH-681-98	Beacharof Lake	57.500	-156.070	AK	1998-00-00	Muscle		-21.2	48.6	14.0	3.5	-21.9	*-18.2	n	0.8	-17.4	11.4				2		
Satterfield and Finney 2002		BECH-6-97	Beacharof Lake	57.500	-156.070	AK	1997-00-00	Muscle		-20.5	56.2	16.2	3.5	-21.2	*-17.5	n	0.7	-16.8	11.6				2		
Satterfield and Finney 2002		BECH-735-98	Beacharof Lake	57.500	-156.070	AK	1998-00-00	Muscle		-21.0	49.3	14.2	3.5	-21.6	*-17.9	n	0.8	-17.2	11.4				2		
Satterfield and Finney 2002		BECH-620-98	Beacharof Lake	57.500	-156.070	AK	1998-00-00	Muscle		-20.6	49.2	14.1	3.5	-21.3	*-17.6	n	0.8	-16.8	10.7				2		
Satterfield and Finney 2002		BECH-605-98	Beacharof Lake	57.500	-156.070	AK	1998-00-00	Muscle		-20.7	49.1	14.1	3.5	-21.3	*-17.6	n	0.8	-16.9	11.6				2		
Satterfield and Finney 2002		BECH-2-97	Beacharof Lake	57.500	-156.070	AK	1997-00-00	Muscle		-20.5	62.2	17.8	3.5	-21.2	*-17.5	n	0.7	-16.8	11.2				2		
Satterfield and Finney 2002		BECH-645-98	Beacharof Lake	57.500	-156.070	AK	1998-00-00	Muscle		-20.4	49.0	14.0	3.5	-21.1	*-17.4	n	0.8	-16.7	10.6				2		
Satterfield and Finney 2002		BECH-610-98	Beacharof Lake	57.500	-156.070	AK	1998-00-00	Muscle		-21.2	48.9	14.0	3.5	-21.9	*-18.2	n	0.8	-17.4	11.3				2		
Satterfield and Finney 2002		BECH-608-98	Beacharof Lake	57.500	-156.070	AK	1998-00-00	Muscle		-21.1	49.7	14.2	3.5	-21.7	*-18.0	n	0.8	-17.3	11.1				2		
Satterfield and Finney 2002		BECH-7-97	Beacharof Lake	57.500	-156.070	AK	1997-00-00	Muscle		-20.2	57.7	16.4	3.5	-20.8	*-17.2	n	0.7	-16.4	11.3				2		
Satterfield and Finney 2002		BECH-601-98	Beacharof Lake	57.500	-156.070	AK	1998-00-00	Muscle		-20.7	48.8	13.9	3.5	-21.3	*-17.6	n	0.8	-16.8	10.8				2		
Satterfield and Finney 2002		BECH-1-97	Beacharof Lake	57.500	-156.070	AK	1997-00-00	Muscle		-20.9	52.1	14.8	3.5	-21.5	*-17.8	n	0.7	-17.1	11.1				2		
Satterfield and Finney 2002		BECH-607-98	Beacharof Lake	57.500	-156.070	AK	1998-00-00	Muscle		-20.9	48.7	13.8	3.5	-21.5	*-17.8	n	0.8	-17.1	11.5				2		
Satterfield and Finney 2002		BECH-600-98	Beacharof Lake	57.500	-156.070	AK	1998-00-00	Muscle		-21.5	50.1	14.2	3.5	-22.1	*-18.4	n	0.8	-17.7	10.6				2		
Satterfield and Finney 2002		BECH-3-97	Beacharof Lake	57.500	-156.070	AK	1997-00-00	Muscle		-21.7	51.5	14.5	3.6	-22.3	*-18.6	n	0.7	-17.8	10.0				2		
Satterfield and Finney 2002		BECH-606-98	Beacharof Lake	57.500	-156.070	AK	1998-00-00	Muscle		-20.4	49.6	13.8	3.6	-20.9	*-17.3	n	0.8	-16.5	11.5				2		
Satterfield and Finney 2002		BECH-604-98	Beacharof Lake	57.500	-156.070	AK	1998-00-00	Muscle		-20.4	49.7	13.9	3.6	-21.0	*-17.3	n	0.8	-16.5	10.5				2		
Satterfield and Finney 2002		BECH-625-98	Beacharof Lake	57.500	-156.070	AK	1998-00-00	Muscle		-20.4	49.9	13.7	3.6	-20.9	*-17.2	n	0.8	-16.4	11.7				2		
Satterfield and Finney 2002		BECH-602-98	Beacharof Lake	57.500	-156.070	AK	1998-00-00	Muscle		-20.2	50.4	13.8	3.6	-20.6	*-16.9	n	0.8	-16.2	12.6				2		
Satterfield and Finney 2002		BEN-1	Benzemen Lake	56.470	-134.550	AK	1996-00-00	Muscle		-20.9	50.6	14.3	3.6	-21.5	*-17.8	n	0.7	-17.1	11.4				2		
Satterfield and Finney 2002		KAT-RS-6	Chilkat Lake	59.150	-125.530	AK	1997-00-00	Muscle		-20.8	46.4	14.3	3.3	-21.9	*-18.2	n	0.7	-17.4	10.8				2		
Satterfield and Finney 2002		KAT-RS-10	Chilkat Lake	59.150	-125.530	AK	1997-00-00	Muscle		-21.0	48.2	14.7	3.3	-22.0	*-18.3	n	0.7	-17.5	10.1				2		
Satterfield and Finney 2002		KAT-RS-2	Chilkat Lake	59.150	-125.530	AK	1997-00-00	Muscle		-21.0	47.9	14.2	3.4	-21.8	*-18.1	n	0.7	-17.4	10.5				2		
Satterfield and Finney 2002		KAT-17	Chilkat Lake	59.150	-125.530	AK	1990-00-00	Muscle		-20.6	53.2	11.7	4.5	-20.0	*-16.3	n	0.6	-15.7	12.4				2		
Satterfield and Finney 2002		KAT-11	Chilkat Lake	59.150	-125.530	AK	1990-00-00	Muscle		-23.1	54.6	10.6	5.2	-22.1	*-18.4	n	0.6	-17.8	10.9				2		
Satterfield and Finney 2002		KAT-24	Chilkat Lake	59.150	-125.530	AK	1990-00-00	Muscle		-25.3	58.9	7.1	8.4	-23.0	*-19.3	n	0.6	-18.7	11.4				2		C:N unacceptable
Welch and Parsons 1993		NA	Chilko Lake	51.287	-124.065	BC	1991-00-00	Muscle						-21.6	*-17.9	n	0.6	-17.3	9.4				2		
Welch and Parsons 1993		NA	Chilko Lake	51.287	-124.065	BC	1991-00-00	Muscle						-21.1	*-17.4	n	0.6	-16.8	9.5				2		
Welch and Parsons 1993		NA	Chilko Lake	51.287	-124.065	BC	1991-00-00	Muscle						-21.1	*-17.4	n	0.6	-16.7	9.5				2		
Welch and Parsons 1993		NA	Chilko Lake	51.287	-124.065	BC	1991-00-00	Muscle						-20.8	*-17.1	n	0.6	-16.5	10.1				2		
Welch and Parsons 1993		NA	Chilko Lake	51.287	-124.065	BC	1991-00-00	Muscle						-20.7	*-17.0	n	0.6	-16.4	10.0				2		
Welch and Parsons 1993		NA	Chilko Lake	51.287	-124.065	BC	1991-00-00	Muscle						-20.7	*-17.0	n	0.6	-16.4	10.5				2		
Welch and Parsons 1993		NA	Chilko Lake	51.287	-124.065	BC	1991-00-00	Muscle						-20.1	*-16.4	n	0.6	-15.8	10.7				2		
Welch and Parsons 1993		NA	Chilko Lake	51.287	-124.065	BC	1991-00-00	Muscle						-20.1	*-16.4	n	0.6	-15.7	11.1				2		
Welch and Parsons 1993		NA	Chilko Lake	51.287	-124.065	BC	1991-00-00	Muscle						-20.0	*-16.3	n	0.6	-15.7	10.8				2		
Welch and Parsons 1993		NA	Chilko Lake	51.287	-124.065	BC	1991-00-00	Muscle						-19.9	*-16.2	n	0.6	-15.6	11.1				2		
Welch and Parsons 1993		NA	Chilko Lake	51.287	-124.065	BC	1991-00-00	Muscle						-19.7	*-16.0	n	0.6	-15.4	11.4				2		
Welch and Parsons 1993		NA	Chilko Lake	51.287	-124.065	BC	1991-00-00	Muscle						-19.5	*-15.8	n	0.6	-15.2	11.3				2		
Satterfield and Finney 2002		KOOT-RS6	Chilkoot Lake	59.210	-135.500	AK	1997-00-00	Muscle		-19.9	47.5	14.8	3.2	-21.1	*-17.4	n	0.7	-16.6	10.9				2		
Satterfield and Finney 2002		KOOT-RS2	Chilkoot Lake	59.210	-135.500	AK	1997-00-00	Muscle		-20.4	48.4	14.9	3.3	-21.5	*-17.8	n	0.7	-17.1	10.5				2		
Satterfield and Finney 2002		KOOT-RS9	Chilkoot Lake	59.210	-135.500	AK	1997-00-00	Muscle		-20.2	49.8	14.8	3.4	-21.1	*-17.4	n	0.7	-16.6	11.6				2		
Satterfield and Finney 2002		KOOT-16	Chilkoot Lake	59.210	-135.500	AK	1990-00-00	Muscle		-20.4	49.7	14.1	3.5	-21.0	*-17.4	n	0.6	-16.7	11.1				2		
Wang et al. 2018		SP06	Copper River	61.501	-144.419	AK	2016-00-00	Muscle						-22.2	*-18.5	n	1.2	-17.3	11.4				2		

Source/Donor	Lab No.	Cat No	Site Name	Latitude	Longitude	Prov. State	Capture Date	Material	Col. Yld. %	Mus. $\delta^{13}C$	Mus. %C	Mus. %N	Mus. C:N	Mus. $\delta^{13}C$, lipid cor.	Col. $\delta^{13}C$	Form. cor.	Suess cor. (‰)	$\delta^{13}C_{cor}$	$\delta^{15}N$	Col. %C	Col. %N	Col. C:N	*A priori* ecotype	$\delta^{13}C$ ecotype ID	Notes
Wang et al. 2018		SP02	Copper River	61.501	-144.419	AK	2016-00-00	Muscle						-22.0	*-18.3	n	1.2	-17.1	10.7				2		
Wang et al. 2018		SP03	Copper River	61.501	-144.419	AK	2016-00-00	Muscle						-21.9	*-18.2	n	1.2	-17.0	10.6				2		
Wang et al. 2018		SP07	Copper River	61.501	-144.419	AK	2016-00-00	Muscle						-21.9	*-18.2	n	1.2	-17.0	11.4				2		
Wang et al. 2018		SP01	Copper River	61.501	-144.419	AK	2016-00-00	Muscle						-21.6	*-17.9	n	1.2	-16.7	11.0				2		
Satterfield and Finney 2002		CRES-52	Cresent Lake	60.220	-152.560	AK	1991-00-00	Muscle		-21.0	47.6	14.3	3.3	-21.9	*-18.2	n	0.6	-17.5	10.2				2		
Satterfield and Finney 2002		CRES-27	Cresent Lake	60.220	-152.560	AK	1991-00-00	Muscle		-19.9	47.8	14.3	3.4	-20.8	*-17.1	n	0.6	-16.5	10.0				2		
Satterfield and Finney 2002		CRES-63	Cresent Lake	60.220	-152.560	AK	1991-00-00	Muscle		-20.3	49.2	13.5	3.7	-20.8	*-17.1	n	0.6	-16.4	10.9				2		
Welch and Parsons 1993		NA	Early Stewart	54.537	-124.526	BC	1991-00-00	Muscle						-20.9	*-17.2	n	0.6	-16.6	11.9				2		
Welch and Parsons 1993		NA	Early Stewart	54.537	-124.526	BC	1991-00-00	Muscle						-20.7	*-17.0	n	0.6	-16.4	12.6				2		
Welch and Parsons 1993		NA	Early Stewart	54.537	-124.526	BC	1991-00-00	Muscle						-20.6	*-16.9	n	0.6	-16.3	12.0				2		
Welch and Parsons 1993		NA	Early Stewart	54.537	-124.526	BC	1991-00-00	Muscle						-20.5	*-16.9	n	0.6	-16.2	12.5				2		
Welch and Parsons 1993		NA	Early Stewart	54.537	-124.526	BC	1991-00-00	Muscle						-20.5	*-16.8	n	0.6	-16.2	12.4				2		
Welch and Parsons 1993		NA	Early Stewart	54.537	-124.526	BC	1991-00-00	Muscle						-20.5	*-16.8	n	0.6	-16.2	12.6				2		
Welch and Parsons 1993		NA	Early Stewart	54.537	-124.526	BC	1991-00-00	Muscle						-20.5	*-16.8	n	0.6	-16.2	11.9				2		
Welch and Parsons 1993		NA	Early Stewart	54.537	-124.526	BC	1991-00-00	Muscle						-20.5	*-16.8	n	0.6	-16.2	12.4				2		
Welch and Parsons 1993		NA	Early Stewart	54.537	-124.526	BC	1991-00-00	Muscle						-20.5	*-16.8	n	0.6	-16.1	12.1				2		
Welch and Parsons 1993		NA	Early Stewart	54.537	-124.526	BC	1991-00-00	Muscle						-20.3	*-16.6	n	0.6	-15.9	12.6				2		
Welch and Parsons 1993		NA	Early Stewart	54.537	-124.526	BC	1991-00-00	Muscle						-20.2	*-16.5	n	0.6	-15.9	12.4				2		
Welch and Parsons 1993		NA	Early Stewart	54.537	-124.526	BC	1991-00-00	Muscle						-20.1	*-16.4	n	0.6	-15.7	11.4				2		
Satterfield and Finney 2002		EVA-46	Eva Lake	57.400	-135.700	AK	1995-00-00	Muscle		-20.6	47.6	15.0	3.2	-21.7	*-18.1	n	0.7	-17.4	11.1				2		
Satterfield and Finney 2002		EVA-10	Eva Lake	57.400	-135.700	AK	1995-00-00	Muscle		-21.2	48.6	14.3	3.4	-22.0	*-18.3	n	0.7	-17.6	11.0				2		
Satterfield and Finney 2002		EVA-44	Eva Lake	57.400	-135.700	AK	1995-00-00	Muscle		-20.8	48.5	13.8	3.5	-21.4	*-17.7	n	0.7	-17.0	12.8				2		
Kaeriyama et al. 2004		NA	Gulf of Alaska	50.560	-145.000		1999-00-00	Muscle						-21.6	*-17.9	n	0.8	-17.1	11.7				2		
Kaeriyama et al. 2004		NA	Gulf of Alaska	50.560	-145.000		1999-00-00	Muscle						-21.5	*-17.8	n	0.8	-17.0	11.5				2		
Kaeriyama et al. 2004		NA	Gulf of Alaska	50.560	-145.000		1999-00-00	Muscle						-21.3	*-17.6	n	0.8	-16.8	10.7				2		
Kaeriyama et al. 2004		NA	Gulf of Alaska	50.560	-145.000		1999-00-00	Muscle						-21.2	*-17.5	n	0.8	-16.7	11.5				2		
Kaeriyama et al. 2004		NA	Gulf of Alaska	50.560	-145.000		1999-00-00	Muscle						-21.2	*-17.5	n	0.8	-16.7	12.7				2		
Kaeriyama et al. 2004		NA	Gulf of Alaska	50.560	-145.000		1999-00-00	Muscle						-21.1	*-17.4	n	0.8	-16.6	12.5				2		
Kaeriyama et al. 2004		NA	Gulf of Alaska	50.560	-145.000		1999-00-00	Muscle						-20.9	*-17.2	n	0.8	-16.4	11.3				2		
Kaeriyama et al. 2004		NA	Gulf of Alaska	50.560	-145.000		1999-00-00	Muscle						-20.8	*-17.1	n	0.8	-16.3	12.6				2		
Kaeriyama et al. 2004		NA	Gulf of Alaska	50.560	-145.000		1999-00-00	Muscle						-20.7	*-17.0	n	0.8	-16.3	11.9				2		
Kaeriyama et al. 2004		NA	Gulf of Alaska	50.560	-145.000		1999-00-00	Muscle						-20.7	*-17.0	n	0.8	-16.2	11.5				2		
Kaeriyama et al. 2004		NA	Gulf of Alaska	50.560	-145.000		1999-00-00	Muscle						-20.7	*-17.0	n	0.8	-16.2	11.7				2		
Kaeriyama et al. 2004		NA	Gulf of Alaska	50.560	-145.000		1999-00-00	Muscle						-20.6	*-17.0	n	0.8	-16.2	11.4				2		
Kaeriyama et al. 2004		NA	Gulf of Alaska	50.560	-145.000		1999-00-00	Muscle						-20.5	*-16.8	n	0.8	-16.0	11.3				2		
Kaeriyama et al. 2004		NA	Gulf of Alaska	50.560	-145.000		1999-00-00	Muscle						-20.2	*-16.5	n	0.8	-15.7	12.0				2		
Kaeriyama et al. 2004		NA	Gulf of Alaska	50.560	-145.000		1999-00-00	Muscle						-20.0	*-16.3	n	0.8	-15.5	11.3				2		
Kaeriyama et al. 2004		NA	Gulf of Alaska	50.560	-145.000		1999-00-00	Muscle						-19.8	*-16.1	n	0.8	-15.3	10.6				2		
Kaeriyama et al. 2004		NA	Gulf of Alaska	50.560	-145.000		1999-00-00	Muscle						-19.6	*-15.9	n	0.8	-15.1	11.2				2		
Kaeriyama et al. 2004		NA	Gulf of Alaska	50.560	-145.000		1999-00-00	Muscle						-19.5	*-15.8	n	0.8	-15.0	10.5				2		
Kaeriyama et al. 2004		NA	Gulf of Alaska	50.560	-145.000		2000-00-00	Muscle						-20.7	*-17.0	n	0.8	-16.2	10.4				2		
Kaeriyama et al. 2004		NA	Gulf of Alaska	50.560	-145.000		2000-00-00	Muscle						-20.6	*-16.9	n	0.8	-16.1	9.8				2		
Kaeriyama et al. 2004		NA	Gulf of Alaska	50.560	-145.000		2000-00-00	Muscle						-19.7	*-16.0	n	0.8	-15.2	11.0				2		
Kaeriyama et al. 2004		NA	Gulf of Alaska	50.560	-145.000		2000-00-00	Muscle						-19.5	*-15.8	n	0.8	-15.0	12.6				2		
Kaeriyama et al. 2004		NA	Gulf of Alaska	50.560	-145.000		2000-00-00	Muscle						-19.5	*-15.8	n	0.8	-15.0	10.1				2		
Kaeriyama et al. 2004		NA	Gulf of Alaska	50.560	-145.000		2000-00-00	Muscle						-19.5	*-15.8	n	0.8	-15.0	11.3				2		
Kaeriyama et al. 2004		NA	Gulf of Alaska	50.560	-145.000		2000-00-00	Muscle						-19.4	*-15.8	n	0.8	-14.9	12.2				2		
Kaeriyama et al. 2004		NA	Gulf of Alaska	50.560	-145.000		2000-00-00	Muscle						-19.4	*-15.7	n	0.8	-14.9	11.9				2		
Kaeriyama et al. 2004		NA	Gulf of Alaska	50.560	-145.000		2000-00-00	Muscle						-19.4	*-15.7	n	0.8	-14.9	11.3				2		
Kaeriyama et al. 2004		NA	Gulf of Alaska	50.560	-145.000		2000-00-00	Muscle						-19.4	*-15.7	n	0.8	-14.9	10.9				2		

Source/Donor	Lab No.	Cat No	Site Name	Latitude	Longitude	Prov. State	Capture Date	Material	Col. Yld. %	Mus. $\delta^{13}C$	Mus. %C	Mus. %N	Mus. C:N	Mus. $\delta^{13}C$, lipid cor.	Col. $\delta^{13}C$	Form. cor.	Suess cor. (‰)	$\delta^{13}C_{cor}$	$\delta^{15}N$	Col. %C	Col. %N	Col. C:N	*A priori* ecotype	$\delta^{13}C$ ecotype ID	Notes
Kaeriyama et al. 2004		NA	Gulf of Alaska	50.560	-145.000		2000-00-00	Muscle						-19.3	*-15.6	n	0.8	-14.8	11.4				2		
Kaeriyama et al. 2004		NA	Gulf of Alaska	50.560	-145.000		2000-00-00	Muscle						-19.3	*-15.6	n	0.8	-14.8	11.9				2		
Kaeriyama et al. 2004		NA	Gulf of Alaska	50.560	-145.000		2000-00-00	Muscle						-19.3	*-15.6	n	0.8	-14.8	10.3				2		
Kaeriyama et al. 2004		NA	Gulf of Alaska	50.560	-145.000		2000-00-00	Muscle						-19.3	*-15.6	n	0.8	-14.8	11.2				2		
Kaeriyama et al. 2004		NA	Gulf of Alaska	50.560	-145.000		2000-00-00	Muscle						-19.2	*-15.5	n	0.8	-14.7	11.0				2		
Kaeriyama et al. 2004		NA	Gulf of Alaska	50.560	-145.000		2000-00-00	Muscle						-19.2	*-15.5	n	0.8	-14.7	11.8				2		
Kaeriyama et al. 2004		NA	Gulf of Alaska	50.560	-145.000		2000-00-00	Muscle						-18.8	*-15.1	n	0.8	-14.3	10.7				2		
Kaeriyama et al. 2004		NA	Gulf of Alaska	50.560	-145.000		2000-00-00	Muscle						-18.8	*-15.1	n	0.8	-14.3	10.7				2		
Kaeriyama et al. 2004		NA	Gulf of Alaska	50.560	-145.000		2000-00-00	Muscle						-18.7	*-15.0	n	0.8	-14.2	12.4				2		
Kaeriyama et al. 2004		NA	Gulf of Alaska	50.560	-145.000		2000-00-00	Muscle						-18.7	*-15.0	n	0.8	-14.2	10.7				2		
Kaeriyama et al. 2004		NA	Gulf of Alaska	50.560	-145.000		2000-00-00	Muscle						-18.5	*-14.8	n	0.8	-14.0	11.8				2		
Kaeriyama et al. 2004		NA	Gulf of Alaska	50.560	-145.000		2000-00-00	Muscle						-18.1	*-14.4	n	0.8	-13.6	11.9				2		
Qin and Kaeriyama 2016		122	Gulf of Alaska	49.430	-180.000		2006-00-00	Muscle			45.6	14.1	3.8	-20.7	*-17.0	n	0.9	-16.1	11.4				2		
Qin and Kaeriyama 2016		120	Gulf of Alaska	49.040	-173.550		2006-00-00	Muscle			46.0	14.5	3.7	-19.9	*-16.2	n	0.9	-15.3	11.3				2		
Welch and Parsons 1993		NA	High Seas	47.407	177.749		1991-00-00	Muscle						-20.0	*-16.3	n	0.6	-15.7	10.8				2		
Welch and Parsons 1993		NA	High Seas	47.407	177.749		1991-00-00	Muscle						-19.7	*-16.0	n	0.6	-15.4	10.9				2		
Welch and Parsons 1993		NA	High Seas	47.407	177.749		1991-00-00	Muscle						-19.5	*-15.8	n	0.6	-15.2	11.1				2		
Welch and Parsons 1993		NA	High Seas	47.407	177.749		1991-00-00	Muscle						-19.5	*-15.8	n	0.6	-15.1	10.8				2		
Welch and Parsons 1993		NA	High Seas	47.407	177.749		1991-00-00	Muscle						-19.2	*-15.5	n	0.6	-14.9	12.1				2		
Satterfield and Finney 2002		HUGH-SM-31	Huge Smith Lake	55.060	-130.420	AK	1992-00-00	Muscle		-21.4	49.1	12.2	4.0	-21.4	*-17.7	n	0.6	-17.1	11.6				2		
Satterfield and Finney 2002		HUGH-SM-30	Huge Smith Lake	55.060	-130.420	AK	1992-00-00	Muscle		-21.4	50.0	11.8	4.2	-21.2	*-17.5	n	0.6	-16.8	11.1				2		
Satterfield and Finney 2002		HUGH-SM-47	Huge Smith Lake	55.060	-130.420	AK	1996-00-00	Muscle		-22.8	51.3	8.6	6.0	-21.3	*-17.6	n	0.7	-16.9	11.9				2		
Satterfield and Finney 2002		ILIAM-97-6	Iliamna Lake	59.537	-155.167	AK	1997-00-00	Muscle		-19.8	37.4	11.8	3.2	-21.0	*-17.3	n	0.7	-16.6	11.0				2		
Satterfield and Finney 2002		ILIAM-97-2	Iliamna Lake	59.537	-155.167	AK	1997-00-00	Muscle		-19.8	56.8	17.0	3.3	-20.8	*-17.1	n	0.7	-16.3	11.9				2		
Satterfield and Finney 2002		ILIAM-97-7	Iliamna Lake	59.537	-155.167	AK	1997-00-00	Muscle		-19.8	54.9	16.3	3.4	-20.6	*-16.9	n	0.7	-16.2	11.3				2		
Satterfield and Finney 2002		ILIAM-97-4	Iliamna Lake	59.537	-155.167	AK	1997-00-00	Muscle		-21.7	59.4	16.2	3.7	-22.2	*-18.5	n	0.7	-17.7	10.2				2		
Satterfield and Finney 2002		ILIAM-97-5	Iliamna Lake	59.537	-155.167	AK	1997-00-00	Muscle		-20.1	59.0	15.8	3.7	-20.5	*-16.8	n	0.7	-16.0	10.7				2		
Satterfield and Finney 2002		ILIAM-97-9	Iliamna Lake	59.537	-155.167	AK	1997-00-00	Muscle		-20.1	56.7	14.0	4.0	-20.0	*-16.3	n	0.7	-15.6	11.4				2		
Satterfield and Finney 2002		ILIAM-97-1	Iliamna Lake	59.537	-155.167	AK	1997-00-00	Muscle		-20.9	60.7	14.7	4.1	-20.7	*-17.1	n	0.7	-16.3	11.5				2		
Satterfield and Finney 2002		ILIAM-97-8	Iliamna Lake	59.537	-155.167	AK	1997-00-00	Muscle		-21.1	58.2	13.2	4.4	-20.6	*-16.9	n	0.7	-16.2	11.4				2		
Satterfield and Finney 2002		ILIAM-97-10	Iliamna Lake	59.537	-155.167	AK	1997-00-00	Muscle		-21.0	58.3	12.0	4.8	-20.2	*-16.5	n	0.7	-15.7	12.7				2		
Satterfield and Finney 2002		ILIAM-97-3	Iliamna Lake	59.537	-155.167	AK	1997-00-00	Muscle		-21.1	66.3	13.6	4.9	-20.3	*-16.6	n	0.7	-15.9	11.7				2		
Welch and Parsons 1993		NA	Iliamna Lake	59.537	-155.167	AK	1991-00-00	Muscle						-20.7	*-17.0	n	0.6	-16.4	12.3				2		
Welch and Parsons 1993		NA	Iliamna Lake	59.537	-155.167	AK	1991-00-00	Muscle						-20.6	*-16.9	n	0.6	-16.3	11.8				2		
Welch and Parsons 1993		NA	Iliamna Lake	59.537	-155.167	AK	1991-00-00	Muscle						-20.5	*-16.8	n	0.6	-16.2	12.1				2		
Welch and Parsons 1993		NA	Iliamna Lake	59.537	-155.167	AK	1991-00-00	Muscle						-20.5	*-16.8	n	0.6	-16.2	11.9				2		
Welch and Parsons 1993		NA	Iliamna Lake	59.537	-155.167	AK	1991-00-00	Muscle						-20.5	*-16.8	n	0.6	-16.2	12.5				2		
Welch and Parsons 1993		NA	Iliamna Lake	59.537	-155.167	AK	1991-00-00	Muscle						-20.5	*-16.8	n	0.6	-16.2	11.4				2		
Welch and Parsons 1993		NA	Iliamna Lake	59.537	-155.167	AK	1991-00-00	Muscle						-20.3	*-16.7	n	0.6	-16.0	12.7				2		
Welch and Parsons 1993		NA	Iliamna Lake	59.537	-155.167	AK	1991-00-00	Muscle						-20.3	*-16.6	n	0.6	-16.0	11.5				2		
Welch and Parsons 1993		NA	Iliamna Lake	59.537	-155.167	AK	1991-00-00	Muscle						-20.2	*-16.5	n	0.6	-15.9	12.1				2		
Welch and Parsons 1993		NA	Iliamna Lake	59.537	-155.167	AK	1991-00-00	Muscle						-20.0	*-16.3	n	0.6	-15.7	12.1				2		
Welch and Parsons 1993		NA	Iliamna Lake	59.537	-155.167	AK	1991-00-00	Muscle						-19.9	*-16.2	n	0.6	-15.5	11.8				2		
Welch and Parsons 1993		NA	Iliamna Lake	59.537	-155.167	AK	1991-00-00	Muscle						-19.6	*-15.9	n	0.6	-15.3	13.6				2		
Satterfield and Finney 2002		KAR-98-4	Karluk Lake	57.250	-154.050	AK	1998-00-00	Muscle		-20.1	49.4	14.8	3.3	-21.0	*-17.3	n	0.8	-16.5	11.0				2		
Satterfield and Finney 2002		KAR-98-1	Karluk Lake	57.250	-154.050	AK	1998-00-00	Muscle		-20.5	49.2	14.6	3.4	-21.3	*-17.7	n	0.8	-16.9	10.8				2		
Satterfield and Finney 2002		KAR-98-3	Karluk Lake	57.250	-154.050	AK	1998-00-00	Muscle		-20.8	49.5	14.6	3.4	-21.6	*-17.9	n	0.8	-17.2	10.3				2		
Satterfield and Finney 2002		KAR-98-5	Karluk Lake	57.250	-154.050	AK	1998-00-00	Muscle		-20.0	50.0	14.7	3.4	-20.8	*-17.1	n	0.8	-16.3	11.4				2		
Satterfield and Finney 2002		KAR-98-2	Karluk Lake	57.250	-154.050	AK	1998-00-00	Muscle		-20.8	50.6	14.3	3.5	-21.3	*-17.7	n	0.8	-16.9	11.1				2		
Guiry et al. 2016		SUBC 9321	Pacific Ocean	49.123	-123.184	BC	2015-00-00	Bone , vertebra	18.9						-18.4	n	1.2	-17.2	10.7	39.4	14.2	3.23	2		

Source/Donor	Lab No.	Cat No	Site Name	Latitude	Longitude	Prov. State	Capture Date	Material	Col. Yld. %	Mus. $\delta^{13}C$	Mus. %C	Mus. %N	Mus. C:N	Mus. $\delta^{13}C$, lipid cor.	Col. $\delta^{13}C$	Form. cor.	Suess cor. (‰)	$\delta^{13}C_{cor}$	$\delta^{15}N$	Col. %C	Col. %N	Col. C:N	*A priori* ecotype	$\delta^{13}C$ ecotype ID	Notes
Horii et al. 2015		ON	Pacific Ocean	54.594	170.000		2014-00-00	Muscle						-20.4	*-16.7	n	1.1	-15.6	12.5				2		
Guiry et al. 2016		SUBC 10161	Prince Rupert Harbour	54.320	-130.321	BC	2015-06-00	Bone , vertebra	16.1						-17.5	n	1.2	-16.3	10.5	40.4	15.1	3.13	2		
Guiry et al. 2016		SUBC 10153	Prince Rupert Harbour	54.320	-130.321	BC	2015-06-00	Bone , vertebra	16.4						-17.7	n	1.2	-16.5	10.7	40.1	14.8	3.16	2		
Satterfield and Finney 2002		R-662	Red Lake	57.150	-154.200	AK	1966-00-00	Scale							-17.9	n	0.3	-17.6	9.1	27.7	11.3	2.86	2		C:N unacceptable
Satterfield and Finney 2002		R-902	Red Lake	57.150	-154.200	AK	1990-00-00	Scale							-17.0	n	0.6	-16.4	11.7	23.8	9.7	2.86	2		C:N unacceptable
Satterfield and Finney 2002		R-951	Red Lake	57.150	-154.200	AK	1995-00-00	Scale							-17.0	n	0.7	-16.3	11.5	24.6	10.0	2.87	2		C:N unacceptable
Satterfield and Finney 2002		R-904	Red Lake	57.150	-154.200	AK	1990-00-00	Scale							-17.4	n	0.6	-16.8	12.4	26.2	10.6	2.88	2		C:N unacceptable
Satterfield and Finney 2002		R-825	Red Lake	57.150	-154.200	AK	1982-00-00	Scale							-17.8	n	0.5	-17.3	8.6	23.6	9.5	2.90	2		C:N unacceptable
Satterfield and Finney 2002		R-861	Red Lake	57.150	-154.200	AK	1986-00-00	Scale							-16.7	n	0.6	-16.1	8.4	24.9	12.8	2.27	2		C:N unacceptable
Satterfield and Finney 2002		R-831	Red Lake	57.150	-154.200	AK	1983-00-00	Scale							-16.5	n	0.5	-16.0	9.7	24.3	11.3	2.51	2		C:N unacceptable
Satterfield and Finney 2002		R-842	Red Lake	57.150	-154.200	AK	1984-00-00	Scale							-17.4	n	0.5	-16.8	7.2	25.9	11.4	2.65	2		C:N unacceptable
Satterfield and Finney 2002		R-762	Red Lake	57.150	-154.200	AK	1976-00-00	Scale							-17.4	n	0.4	-17.0	8.3	26.6	11.6	2.67	2		C:N unacceptable
Satterfield and Finney 2002		R-705	Red Lake	57.150	-154.200	AK	1970-00-00	Scale							-18.0	n	0.4	-17.7	11.0	25.6	11.1	2.69	2		C:N unacceptable
Satterfield and Finney 2002		R-792	Red Lake	57.150	-154.200	AK	1979-00-00	Scale							-17.4	n	0.5	-16.9	9.6	24.7	10.7	2.69	2		C:N unacceptable
Satterfield and Finney 2002		R-823	Red Lake	57.150	-154.200	AK	1982-00-00	Scale							-16.5	n	0.5	-16.0	8.5	26.1	11.3	2.69	2		C:N unacceptable
Satterfield and Finney 2002		R-704	Red Lake	57.150	-154.200	AK	1970-00-00	Scale							-18.6	n	0.4	-18.2	8.8	25.9	11.1	2.72	2		C:N unacceptable
Satterfield and Finney 2002		R-981	Red Lake	57.150	-154.200	AK	1998-00-00	Scale							-16.9	n	0.8	-16.1	9.6	24.9	10.6	2.74	2		C:N unacceptable
Satterfield and Finney 2002		R-852	Red Lake	57.150	-154.200	AK	1985-00-00	Scale							-16.8	n	0.5	-16.3	9.4	22.2	9.4	2.75	2		C:N unacceptable
Satterfield and Finney 2002		R-864	Red Lake	57.150	-154.200	AK	1986-00-00	Scale							-17.1	n	0.6	-16.5	12.5	25.1	10.6	2.76	2		C:N unacceptable
Satterfield and Finney 2002		R-941	Red Lake	57.150	-154.200	AK	1994-00-00	Scale							-18.8	n	0.7	-18.1	9.5	24.2	10.2	2.77	2		C:N unacceptable
Satterfield and Finney 2002		R-802	Red Lake	57.150	-154.200	AK	1980-00-00	Scale							-16.1	n	0.5	-15.7	10.2	25.0	10.5	2.78	2		C:N unacceptable
Satterfield and Finney 2002		R-971	Red Lake	57.150	-154.200	AK	1997-00-00	Scale							-17.0	n	0.7	-16.2	10.3	29.4	12.3	2.79	2		C:N unacceptable
Satterfield and Finney 2002		R-692	Red Lake	57.150	-154.200	AK	1969-00-00	Scale							-17.4	n	0.3	-17.1	10.4	25.6	10.7	2.79	2		C:N unacceptable
Satterfield and Finney 2002		R-812	Red Lake	57.150	-154.200	AK	1981-00-00	Scale							-16.1	n	0.5	-15.6	8.2	25.0	10.4	2.80	2		C:N unacceptable
Satterfield and Finney 2002		R-821	Red Lake	57.150	-154.200	AK	1982-00-00	Scale							-17.4	n	0.5	-16.9	7.5	25.4	10.5	2.82	2		C:N unacceptable
Satterfield and Finney 2002		R-862	Red Lake	57.150	-154.200	AK	1986-00-00	Scale							-17.5	n	0.6	-16.9	10.2	25.4	10.5	2.82	2		C:N unacceptable
Satterfield and Finney 2002		R-865	Red Lake	57.150	-154.200	AK	1986-00-00	Scale							-16.7	n	0.6	-16.1	10.5	25.7	10.6	2.83	2		C:N unacceptable
Satterfield and Finney 2002		R-824	Red Lake	57.150	-154.200	AK	1982-00-00	Scale							-17.8	n	0.5	-17.3	8.5	25.4	10.2	2.90	2		
Satterfield and Finney 2002		R-901	Red Lake	57.150	-154.200	AK	1990-00-00	Scale							-17.2	n	0.6	-16.6	11.6	23.7	9.5	2.91	2		
Satterfield and Finney 2002		R-982	Red Lake	57.150	-154.200	AK	1998-00-00	Scale							-17.7	n	0.8	-17.0	10.6	25.2	10.1	2.91	2		
Satterfield and Finney 2002		R-661	Red Lake	57.150	-154.200	AK	1966-00-00	Scale							-18.1	n	0.3	-17.8	9.1	28.0	11.2	2.92	2		
Satterfield and Finney 2002		R-945	Red Lake	57.150	-154.200	AK	1994-00-00	Scale							-16.7	n	0.7	-16.0	11.3	25.5	10.2	2.92	2		
Satterfield and Finney 2002		R-712	Red Lake	57.150	-154.200	AK	1971-00-00	Scale							-18.3	n	0.4	-17.9	10.2	23.1	9.2	2.93	2		
Satterfield and Finney 2002		R-972	Red Lake	57.150	-154.200	AK	1997-00-00	Scale							-17.4	n	0.7	-16.6	9.6	25.9	10.3	2.93	2		
Satterfield and Finney 2002		R-782	Red Lake	57.150	-154.200	AK	1978-00-00	Scale							-17.5	n	0.4	-17.0	9.2	21.9	8.7	2.94	2		
Satterfield and Finney 2002		R-663	Red Lake	57.150	-154.200	AK	1966-00-00	Scale							-17.5	n	0.3	-17.2	8.2	27.7	11.0	2.94	2		
Satterfield and Finney 2002		R-703	Red Lake	57.150	-154.200	AK	1970-00-00	Scale							-18.1	n	0.4	-17.7	8.7	27.0	10.7	2.94	2		
Satterfield and Finney 2002		R-872	Red Lake	57.150	-154.200	AK	1987-00-00	Scale							-18.2	n	0.6	-17.6	9.1	23.6	9.3	2.96	2		
Satterfield and Finney 2002		R-743	Red Lake	57.150	-154.200	AK	1974-00-00	Scale							-17.2	n	0.4	-16.8	9.2	25.4	10.0	2.96	2		
Satterfield and Finney 2002		R-781	Red Lake	57.150	-154.200	AK	1978-00-00	Scale							-17.1	n	0.4	-16.6	10.2	30.8	12.1	2.97	2		
Satterfield and Finney 2002		R-851	Red Lake	57.150	-154.200	AK	1985-00-00	Scale							-17.2	n	0.5	-16.6	9.6	24.0	9.4	2.98	2		
Satterfield and Finney 2002		R-732	Red Lake	57.150	-154.200	AK	1973-00-00	Scale							-17.1	n	0.4	-16.7	10.1	24.8	9.7	2.98	2		
Satterfield and Finney 2002		R-672	Red Lake	57.150	-154.200	AK	1967-00-00	Scale							-17.5	n	0.3	-17.1	10.5	25.6	10.0	2.99	2		
Satterfield and Finney 2002		R-721	Red Lake	57.150	-154.200	AK	1972-00-00	Scale							-17.5	n	0.4	-17.1	11.0	23.5	9.1	3.01	2		
Satterfield and Finney 2002		R-931	Red Lake	57.150	-154.200	AK	1993-00-00	Scale							-18.2	n	0.7	-17.5	10.4	23.5	9.1	3.01	2		
Satterfield and Finney 2002		R-691	Red Lake	57.150	-154.200	AK	1969-00-00	Scale							-17.4	n	0.3	-17.0	11.4	23.6	9.1	3.02	2		
Satterfield and Finney 2002		R-912	Red Lake	57.150	-154.200	AK	1991-00-00	Scale							-17.3	n	0.6	-16.6	11.4	23.1	8.9	3.03	2		
Satterfield and Finney 2002		R-722	Red Lake	57.150	-154.200	AK	1972-00-00	Scale							-18.2	n	0.4	-17.8	10.4	23.9	9.2	3.03	2		
Satterfield and Finney 2002		R-841	Red Lake	57.150	-154.200	AK	1984-00-00	Scale							-17.0	n	0.5	-16.4	10.5	25.0	9.6	3.04	2		
Satterfield and Finney 2002		R-822	Red Lake	57.150	-154.200	AK	1982-00-00	Scale							-18.9	n	0.5	-18.4	8.4	27.1	10.4	3.04	2		

Source/Donor	Lab No.	Cat No	Site Name	Latitude	Longitude	Prov. State	Capture Date	Material	Col. Yld. %	Mus. $\delta^{13}C$	Mus. %C	Mus. %N	Mus. C:N	Mus. $\delta^{13}C$, lipid cor.	Col. $\delta^{13}C$	Form. cor.	Suess cor. (‰)	$\delta^{13}C_{cor}$	$\delta^{15}N$	Col. %C	Col. %N	Col. C:N	*A priori* ecotype	$\delta^{13}C$ ecotype ID	Notes
Satterfield and Finney 2002		R-921	Red Lake	57.150	-154.200	AK	1992-00-00	Scale							-17.1	n	0.6	-16.4	11.8	24.5	9.4	3.04	2		
Satterfield and Finney 2002		R-801	Red Lake	57.150	-154.200	AK	1980-00-00	Scale							-17.7	n	0.5	-17.2	8.9	24.0	9.2	3.04	2		
Satterfield and Finney 2002		R-671	Red Lake	57.150	-154.200	AK	1967-00-00	Scale							-17.5	n	0.3	-17.1	10.9	22.7	8.7	3.04	2		
Satterfield and Finney 2002		R-733	Red Lake	57.150	-154.200	AK	1973-00-00	Scale							-17.1	n	0.4	-16.7	10.1	26.4	10.1	3.05	2		
Satterfield and Finney 2002		R-745	Red Lake	57.150	-154.200	AK	1974-00-00	Scale							-17.4	n	0.4	-17.0	9.9	25.9	9.9	3.05	2		
Satterfield and Finney 2002		R-752	Red Lake	57.150	-154.200	AK	1975-00-00	Scale							-17.2	n	0.4	-16.7	10.3	25.4	9.7	3.05	2		
Satterfield and Finney 2002		R-944	Red Lake	57.150	-154.200	AK	1994-00-00	Scale							-18.6	n	0.7	-17.9	9.8	24.4	9.3	3.06	2		
Satterfield and Finney 2002		R-932	Red Lake	57.150	-154.200	AK	1993-00-00	Scale							-18.7	n	0.7	-18.1	9.5	25.2	9.6	3.06	2		
Satterfield and Finney 2002		R-761	Red Lake	57.150	-154.200	AK	1976-00-00	Scale							-17.3	n	0.4	-16.9	11.4	24.2	9.2	3.07	2		
Satterfield and Finney 2002		R-811	Red Lake	57.150	-154.200	AK	1981-00-00	Scale							-16.9	n	0.5	-16.4	10.5	23.7	9.0	3.07	2		
Satterfield and Finney 2002		R-903	Red Lake	57.150	-154.200	AK	1990-00-00	Scale							-18.0	n	0.6	-17.4	8.9	26.4	10.0	3.08	2		
Satterfield and Finney 2002		R-871	Red Lake	57.150	-154.200	AK	1987-00-00	Scale							-18.2	n	0.6	-17.7	9.3	24.3	9.2	3.08	2		
Satterfield and Finney 2002		R-804	Red Lake	57.150	-154.200	AK	1980-00-00	Scale							-17.5	n	0.5	-17.0	9.7	25.9	9.8	3.08	2		
Satterfield and Finney 2002		R-731	Red Lake	57.150	-154.200	AK	1973-00-00	Scale							-18.0	n	0.4	-17.6	10.1	26.7	10.1	3.08	2		
Satterfield and Finney 2002		R-791	Red Lake	57.150	-154.200	AK	1979-00-00	Scale							-17.6	n	0.5	-17.1	10.1	22.3	8.4	3.10	2		
Satterfield and Finney 2002		R-702	Red Lake	57.150	-154.200	AK	1970-00-00	Scale							-17.8	n	0.4	-17.4	10.5	23.9	9.0	3.10	2		
Satterfield and Finney 2002		R-954	Red Lake	57.150	-154.200	AK	1995-00-00	Scale							-18.4	n	0.7	-17.7	9.9	23.9	9.0	3.10	2		
Satterfield and Finney 2002		R-863	Red Lake	57.150	-154.200	AK	1986-00-00	Scale							-18.3	n	0.6	-17.7	10.1	24.5	9.2	3.11	2		
Satterfield and Finney 2002		R-674	Red Lake	57.150	-154.200	AK	1967-00-00	Scale							-17.2	n	0.3	-16.8	10.8	29.3	11.0	3.11	2		
Satterfield and Finney 2002		R-734	Red Lake	57.150	-154.200	AK	1973-00-00	Scale							-17.5	n	0.4	-17.1	11.0	26.4	9.9	3.11	2		
Satterfield and Finney 2002		R-905	Red Lake	57.150	-154.200	AK	1990-00-00	Scale							-19.0	n	0.6	-18.4	9.3	28.0	10.5	3.11	2		
Satterfield and Finney 2002		R-833	Red Lake	57.150	-154.200	AK	1983-00-00	Scale							-17.9	n	0.5	-17.3	9.6	25.1	9.4	3.11	2		
Satterfield and Finney 2002		R-805	Red Lake	57.150	-154.200	AK	1980-00-00	Scale							-17.2	n	0.5	-16.7	9.5	24.6	9.2	3.12	2		
Satterfield and Finney 2002		R-673	Red Lake	57.150	-154.200	AK	1967-00-00	Scale							-17.5	n	0.3	-17.2	9.5	29.0	10.8	3.13	2		
Satterfield and Finney 2002		R-911	Red Lake	57.150	-154.200	AK	1991-00-00	Scale							-17.0	n	0.6	-16.4	10.5	22.6	8.4	3.14	2		
Satterfield and Finney 2002		R-955	Red Lake	57.150	-154.200	AK	1995-00-00	Scale							-18.6	n	0.7	-17.9	10.1	26.4	9.8	3.14	2		
Satterfield and Finney 2002		R-952	Red Lake	57.150	-154.200	AK	1995-00-00	Scale							-18.4	n	0.7	-17.7	10.0	24.3	9.0	3.15	2		
Satterfield and Finney 2002		R-953	Red Lake	57.150	-154.200	AK	1995-00-00	Scale							-18.0	n	0.7	-17.3	11.0	28.1	10.4	3.15	2		
Satterfield and Finney 2002		R-922	Red Lake	57.150	-154.200	AK	1992-00-00	Scale							-17.1	n	0.6	-16.4	11.5	23.8	8.8	3.15	2		
Satterfield and Finney 2002		R-751	Red Lake	57.150	-154.200	AK	1975-00-00	Scale							-17.3	n	0.4	-16.9	8.4	24.1	8.9	3.16	2		
Satterfield and Finney 2002		R-803	Red Lake	57.150	-154.200	AK	1980-00-00	Scale							-16.5	n	0.5	-16.0	10.0	26.3	9.7	3.16	2		
Satterfield and Finney 2002		R-943	Red Lake	57.150	-154.200	AK	1994-00-00	Scale							-18.8	n	0.7	-18.1	10.3	23.6	8.7	3.16	2		
Satterfield and Finney 2002		R-741	Red Lake	57.150	-154.200	AK	1974-00-00	Scale							-17.4	n	0.4	-17.0	11.0	26.6	9.8	3.17	2		
Satterfield and Finney 2002		R-882	Red Lake	57.150	-154.200	AK	1988-00-00	Scale							-17.6	n	0.6	-17.0	10.6	24.7	9.1	3.17	2		
Satterfield and Finney 2002		R-942	Red Lake	57.150	-154.200	AK	1994-00-00	Scale							-16.7	n	0.7	-16.0	11.1	23.1	8.5	3.17	2		
Satterfield and Finney 2002		R-965	Red Lake	57.150	-154.200	AK	1996-00-00	Scale							-17.4	n	0.7	-16.7	10.5	24.2	8.9	3.17	2		
Satterfield and Finney 2002		R-784	Red Lake	57.150	-154.200	AK	1978-00-00	Scale							-18.1	n	0.4	-17.7	7.1	24.8	9.1	3.18	2		
Satterfield and Finney 2002		R-961	Red Lake	57.150	-154.200	AK	1996-00-00	Scale							-17.5	n	0.7	-16.8	10.3	24.8	9.1	3.18	2		
Satterfield and Finney 2002		R-832	Red Lake	57.150	-154.200	AK	1983-00-00	Scale							-17.3	n	0.5	-16.8	11.2	27.3	10.0	3.18	2		
Satterfield and Finney 2002		R-783	Red Lake	57.150	-154.200	AK	1978-00-00	Scale							-18.6	n	0.4	-18.2	7.6	23.5	8.6	3.19	2		
Satterfield and Finney 2002		R-962	Red Lake	57.150	-154.200	AK	1996-00-00	Scale							-16.1	n	0.7	-15.3	11.0	23.5	8.6	3.19	2		
Satterfield and Finney 2002		R-963	Red Lake	57.150	-154.200	AK	1996-00-00	Scale							-17.5	n	0.7	-16.7	10.8	28.7	10.5	3.19	2		
Satterfield and Finney 2002		R-834	Red Lake	57.150	-154.200	AK	1983-00-00	Scale							-17.2	n	0.5	-16.6	11.1	26.8	9.8	3.19	2		
Satterfield and Finney 2002		R-665	Red Lake	57.150	-154.200	AK	1966-00-00	Scale							-17.2	n	0.3	-16.8	9.8	27.9	10.2	3.19	2		
Satterfield and Finney 2002		R-881	Red Lake	57.150	-154.200	AK	1988-00-00	Scale							-17.8	n	0.6	-17.2	10.5	23.8	8.7	3.19	2		
Satterfield and Finney 2002		R-742	Red Lake	57.150	-154.200	AK	1974-00-00	Scale							-16.5	n	0.4	-16.1	11.2	25.2	9.2	3.19	2		
Satterfield and Finney 2002		R-772	Red Lake	57.150	-154.200	AK	1977-00-00	Scale							-17.7	n	0.4	-17.2	9.2	25.3	9.2	3.21	2		
Satterfield and Finney 2002		R-664	Red Lake	57.150	-154.200	AK	1966-00-00	Scale							-18.2	n	0.3	-17.9	10.3	24.0	8.6	3.25	2		
Satterfield and Finney 2002		R-992	Red Lake	57.150	-154.200	AK	1999-00-00	Scale							-17.3	n	0.8	-16.5	10.6	26.8	9.6	3.26	2		
Satterfield and Finney 2002		R-993	Red Lake	57.150	-154.200	AK	1999-00-00	Scale							-17.6	n	0.8	-16.9	10.0	28.2	10.1	3.26	2		

Source/Donor	Lab No.	Cat No	Site Name	Latitude	Longitude	Prov. State	Capture Date	Material	Col. Yld. %	Mus. $\delta^{13}C$	Mus. %C	Mus. %N	Mus. C:N	Mus. $\delta^{13}C$, lipid cor.	Col. $\delta^{13}C$	Form. cor.	Suess cor. (‰)	$\delta^{13}C_{cor}$	$\delta^{15}N$	Col. %C	Col. %N	Col. C:N	*A priori* ecotype	$\delta^{13}C$ ecotype ID	Notes
Satterfield and Finney 2002		R-891	Red Lake	57.150	-154.200	AK	1989-00-00	Scale							-18.2	n	0.6	-17.6	10.3	25.3	9.0	3.28	2		
Satterfield and Finney 2002		R-675	Red Lake	57.150	-154.200	AK	1967-00-00	Scale							-17.6	n	0.3	-17.2	10.0	49.2	17.5	3.28	2		
Satterfield and Finney 2002		R-771	Red Lake	57.150	-154.200	AK	1977-00-00	Scale							-18.4	n	0.4	-18.0	8.8	26.0	9.2	3.30	2		
Satterfield and Finney 2002		R-701	Red Lake	57.150	-154.200	AK	1970-00-00	Scale							-18.3	n	0.4	-18.0	9.4	22.9	8.1	3.30	2		
Satterfield and Finney 2002		R-711	Red Lake	57.150	-154.200	AK	1971-00-00	Scale							-18.0	n	0.4	-17.6	9.3	25.2	8.9	3.30	2		
Satterfield and Finney 2002		R-964	Red Lake	57.150	-154.200	AK	1996-00-00	Scale							-17.3	n	0.7	-16.6	10.0	26.7	9.4	3.31	2		
Satterfield and Finney 2002		R-785	Red Lake	57.150	-154.200	AK	1978-00-00	Scale							-18.3	n	0.4	-17.8	8.7	24.2	8.5	3.32	2		
Satterfield and Finney 2002		R-892	Red Lake	57.150	-154.200	AK	1989-00-00	Scale							-18.4	n	0.6	-17.8	10.3	23.3	8.1	3.35	2		
Satterfield and Finney 2002		RED-1	Redoubt Lake	56.530	-135.150	AK	1996-00-00	Muscle		-20.4	50.3	15.2	3.3	-21.3	*-17.6	n	0.7	-16.9	11.5				2		
Qin and Kaeriyama 2016		112	South-eastern Bering Sea	56.000	-166.000		2007-00-00	Muscle			45.5	14.6	3.6	-20.1	*-16.4	n	1.0	-15.4	11.0				2		
Qin and Kaeriyama 2016		110	South-eastern Bering Sea	56.000	-166.000		2007-00-00	Muscle			44.5	13.1	4.0	-20.0	*-16.3	n	1.0	-15.3	11.0				2		
Qin and Kaeriyama 2016		111	South-eastern Bering Sea	56.000	-166.000		2007-00-00	Muscle			53.8	15.4	4.1	-19.8	*-16.1	n	1.0	-15.1	11.9				2		
Satterfield and Finney 2002		SPECL-3	Speel Lake	58.120	-133.340	AK	1992-00-00	Muscle		-20.7	48.8	13.6	3.6	-21.3	*-17.6	n	0.6	-16.9	10.9				2		
Satterfield and Finney 2002		SPECL-5	Speel Lake	58.120	-133.340	AK	1992-00-00	Muscle		-21.5	48.8	13.3	3.7	-21.9	*-18.2	n	0.6	-17.6	11.2				2		
Satterfield and Finney 2002		SPECL-6	Speel Lake	58.120	-133.340	AK	1992-00-00	Muscle		-22.4	53.5	10.3	5.2	-21.3	*-17.6	n	0.6	-17.0	12.0				2		
Satterfield and Finney 2002		SWEETHEART-3a,b	Sweetheart Lake	57.580	-133.350	AK	1996-00-00	Muscle		-19.8	47.0	13.8	3.4	-20.6	*-16.9	n	0.7	-16.2	10.9				2		
Satterfield and Finney 2002		SWEETHEART-2	Sweetheart Lake	57.580	-133.350	AK	1996-00-00	Muscle		-21.1	49.4	13.3	3.7	-21.5	*-17.8	n	0.7	-17.1	11.6				2		
Satterfield and Finney 2002		SWEETHEART-4a,b	Sweetheart Lake	57.580	-133.350	AK	1996-00-00	Muscle		-21.0	49.3	12.8	3.8	-21.2	*-17.5	n	0.7	-16.8	10.9				2		
Satterfield and Finney 2002		SWEETHEART-1	Sweetheart Lake	57.580	-133.350	AK	1996-00-00	Muscle		-21.2	51.1	12.8	4.0	-21.2	*-17.5	n	0.7	-16.8	11.9				2		
Welch and Parsons 1993		NA	Takla Lake	55.397	-125.855	BC	1991-00-00	Muscle						-20.8	*-17.1	n	0.6	-16.5	10.6				2		
Welch and Parsons 1993		NA	Takla Lake	55.397	-125.855	BC	1991-00-00	Muscle						-20.7	*-17.0	n	0.6	-16.3	12.4				2		
Welch and Parsons 1993		NA	Takla Lake	55.397	-125.855	BC	1991-00-00	Muscle						-20.7	*-17.0	n	0.6	-16.3	12.1				2		
Welch and Parsons 1993		NA	Takla Lake	55.397	-125.855	BC	1991-00-00	Muscle						-20.7	*-17.0	n	0.6	-16.3	11.6				2		
Welch and Parsons 1993		NA	Takla Lake	55.397	-125.855	BC	1991-00-00	Muscle						-20.6	*-16.9	n	0.6	-16.3	11.2				2		
Welch and Parsons 1993		NA	Takla Lake	55.397	-125.855	BC	1991-00-00	Muscle						-20.6	*-16.9	n	0.6	-16.3	12.1				2		
Welch and Parsons 1993		NA	Takla Lake	55.397	-125.855	BC	1991-00-00	Muscle						-20.5	*-16.8	n	0.6	-16.2	10.7				2		
Welch and Parsons 1993		NA	Takla Lake	55.397	-125.855	BC	1991-00-00	Muscle						-20.4	*-16.7	n	0.6	-16.1	11.6				2		
Welch and Parsons 1993		NA	Takla Lake	55.397	-125.855	BC	1991-00-00	Muscle						-20.3	*-16.6	n	0.6	-16.0	11.4				2		
Welch and Parsons 1993		NA	Takla Lake	55.397	-125.855	BC	1991-00-00	Muscle						-20.3	*-16.6	n	0.6	-16.0	11.7				2		
Welch and Parsons 1993		NA	Takla Lake	55.397	-125.855	BC	1991-00-00	Muscle						-20.2	*-16.6	n	0.6	-15.9	11.7				2		
Welch and Parsons 1993		NA	Takla Lake	55.397	-125.855	BC	1991-00-00	Muscle						-20.1	*-16.4	n	0.6	-15.8	12.3				2		
Qin and Kaeriyama 2016		49	Western Bering Sea	56.590	177.590		2009-00-00	Muscle			30.8	9.8	3.7	-21.1	*-17.4	n	1.0	-16.4	11.1				2		
Qin and Kaeriyama 2016		46	Western Bering Sea	56.590	177.590		2009-00-00	Muscle			46.5	14.9	3.6	-20.9	*-17.2	n	1.0	-16.2	12.0				2		
Qin and Kaeriyama 2016		54	Western Bering Sea	55.410	178.000		2009-00-00	Muscle			47.5	13.6	4.1	-20.9	*-17.2	n	1.0	-16.2	10.9				2		
Qin and Kaeriyama 2016		37	Western Bering Sea	56.010	179.580		2009-00-00	Muscle			48.0	14.7	3.8	-20.8	*-17.1	n	1.0	-16.1	11.4				2		
Qin and Kaeriyama 2016		58	Western Bering Sea	55.410	178.000		2009-00-00	Muscle			47.7	14.5	3.8	-20.7	*-17.0	n	1.0	-16.0	10.8				2		
Qin and Kaeriyama 2016		44	Western Bering Sea	56.590	177.590		2009-00-00	Muscle			46.4	15.3	3.5	-20.6	*-16.9	n	1.0	-15.9	11.3				2		
Qin and Kaeriyama 2016		47	Western Bering Sea	56.590	177.590		2009-00-00	Muscle			44.7	14.5	3.6	-20.6	*-16.9	n	1.0	-15.9	11.9				2		
Qin and Kaeriyama 2016		38	Western Bering Sea	56.010	179.580		2009-00-00	Muscle			46.9	14.1	3.9	-20.6	*-16.9	n	1.0	-15.9	11.8				2		
Qin and Kaeriyama 2016		55	Western Bering Sea	55.410	178.000		2009-00-00	Muscle			45.2	15.2	3.5	-20.6	*-16.9	n	1.0	-15.9	11.9				2		
Qin and Kaeriyama 2016		50	Western Bering Sea	56.590	177.590		2009-00-00	Muscle			46.0	14.8	3.6	-20.5	*-16.8	n	1.0	-15.8	11.7				2		
Qin and Kaeriyama 2016		34	Western Bering Sea	56.010	179.580		2009-00-00	Muscle			48.3	15.3	3.7	-20.4	*-16.7	n	1.0	-15.7	11.2				2		
Qin and Kaeriyama 2016		52	Western Bering Sea	56.590	177.590		2009-00-00	Muscle			42.8	13.7	3.6	-20.4	*-16.7	n	1.0	-15.6	11.9				2		
Qin and Kaeriyama 2016		62	Western Bering Sea	55.410	178.000		2009-00-00	Muscle			43.0	14.1	3.6	-20.4	*-16.7	n	1.0	-15.6	10.3				2		
Qin and Kaeriyama 2016		60	Western Bering Sea	55.410	178.000		2009-00-00	Muscle			44.4	14.9	3.5	-20.3	*-16.6	n	1.0	-15.6	11.1				2		
Qin and Kaeriyama 2016		56	Western Bering Sea	55.410	178.000		2009-00-00	Muscle			48.1	14.8	3.8	-20.3	*-16.6	n	1.0	-15.6	11.5				2		
Qin and Kaeriyama 2016		61	Western Bering Sea	55.410	178.000		2009-00-00	Muscle			45.3	15.1	3.5	-20.1	*-16.4	n	1.0	-15.4	12.0				2		
Qin and Kaeriyama 2016		40	Western Bering Sea	56.010	179.580		2009-00-00	Muscle			31.2	9.8	3.7	-20.1	*-16.4	n	1.0	-15.3	11.9				2		
Qin and Kaeriyama 2016		59	Western Bering Sea	55.410	178.000		2009-00-00	Muscle			46.8	13.3	4.1	-19.9	*-16.2	n	1.0	-15.2	11.9				2		
Qin and Kaeriyama 2016		35	Western Bering Sea	56.010	179.580		2009-00-00	Muscle			49.0	15.2	3.8	-19.9	*-16.2	n	1.0	-15.2	11.6				2		

Source/Donor	Lab No.	Cat No	Site Name	Latitude	Longitude	Prov. State	Capture Date	Material	Col. Yld. %	Mus. $\delta^{13}C$	Mus. %C	Mus. %N	Mus. C:N	Mus. $\delta^{13}C$, lipid cor.	Col. $\delta^{13}C$	Form. cor.	Suess cor. (‰)	$\delta^{13}C_{cor}$	$\delta^{15}N$	Col. %C	Col. %N	Col. C:N	*A priori* ecotype	$\delta^{13}C$ ecotype ID	Notes
Qin and Kaeriyama 2016		36	Western Bering Sea	56.010	179.580		2009-00-00	Muscle			44.2	13.8	3.7	-19.9	*-16.2	n	1.0	-15.2	11.5				2		
Qin and Kaeriyama 2016		51	Western Bering Sea	56.590	177.590		2009-00-00	Muscle			45.9	15.2	3.5	-19.8	*-16.1	n	1.0	-15.1	11.8				2		
Qin and Kaeriyama 2016		57	Western Bering Sea	55.410	178.000		2009-00-00	Muscle			48.6	14.1	4.0	-19.8	*-16.1	n	1.0	-15.1	11.6				2		
Qin and Kaeriyama 2016		53	Western Bering Sea	55.410	178.000		2009-00-00	Muscle			46.0	15.1	3.5	-19.8	*-16.1	n	1.0	-15.1	12.0				2		
Qin and Kaeriyama 2016		45	Western Bering Sea	56.590	177.590		2009-00-00	Muscle			44.3	14.8	3.5	-19.7	*-16.0	n	1.0	-15.0	12.3				2		
Qin and Kaeriyama 2016		42	Western Bering Sea	56.010	179.580		2009-00-00	Muscle			50.6	15.3	3.9	-19.5	*-15.9	n	1.0	-14.8	12.6				2		
Qin and Kaeriyama 2016		43	Western Bering Sea	56.010	179.580		2009-00-00	Muscle			46.6	14.8	3.7	-19.5	*-15.8	n	1.0	-14.7	12.2				2		
Qin and Kaeriyama 2016		48	Western Bering Sea	56.590	177.590		2009-00-00	Muscle			44.4	14.4	3.6	-19.3	*-15.6	n	1.0	-14.6	12.9				2		
Qin and Kaeriyama 2016		39	Western Bering Sea	56.010	179.580		2009-00-00	Muscle			43.4	13.5	3.7	-19.1	*-15.4	n	1.0	-14.3	11.9				2		
Qin and Kaeriyama 2016		41	Western Bering Sea	56.010	179.580		2009-00-00	Muscle			43.6	13.8	3.7	-19.0	*-15.3	n	1.0	-14.3	11.8				2		
Qin and Kaeriyama 2016		90	Western Subarctic Gyre	48.590	165.230		2006-00-00	Muscle			46.0	13.1	4.1	-21.5	*-17.8	n	0.9	-16.8	11.8				2		
Qin and Kaeriyama 2016		88	Western Subarctic Gyre	48.590	165.230		2006-00-00	Muscle			50.0	14.3	4.1	-21.2	*-17.5	n	0.9	-16.6	11.2				2		
Qin and Kaeriyama 2016		89	Western Subarctic Gyre	48.590	165.230		2006-00-00	Muscle			48.1	14.2	4.0	-20.9	*-17.2	n	0.9	-16.3	11.8				2		
Qin and Kaeriyama 2016		72	Western Subarctic Gyre	42.300	155.000		2007-00-00	Muscle						-19.9	*-16.3	n	1.0	-15.3	10.5				2		
Qin and Kaeriyama 2016		71	Western Subarctic Gyre	42.300	155.000		2007-00-00	Muscle						-19.9	*-16.2	n	1.0	-15.2	10.6				2		
Qin and Kaeriyama 2016		13	Western Subarctic Gyre	47.010	160.010		2009-00-00	Muscle			41.9	13.1	3.7	-20.8	*-17.1	n	1.0	-16.1	10.8				2		
Qin and Kaeriyama 2016		11	Western Subarctic Gyre	47.010	160.010		2009-00-00	Muscle			47.2	15.0	3.7	-20.4	*-16.7	n	1.0	-15.7	11.4				2		
Qin and Kaeriyama 2016		20	Western Subarctic Gyre	47.000	167.000		2009-00-00	Muscle			44.5	15.2	3.4	-20.4	*-16.7	n	1.0	-15.7	11.1				2		
Qin and Kaeriyama 2016		15	Western Subarctic Gyre	47.010	160.010		2009-00-00	Muscle			46.3	14.9	3.6	-20.3	*-16.6	n	1.0	-15.6	11.3				2		
Qin and Kaeriyama 2016		14	Western Subarctic Gyre	47.010	160.010		2009-00-00	Muscle			45.8	15.0	3.5	-20.2	*-16.5	n	1.0	-15.5	11.3				2		
Qin and Kaeriyama 2016		12	Western Subarctic Gyre	47.010	160.010		2009-00-00	Muscle			46.9	14.9	3.7	-20.0	*-16.3	n	1.0	-15.3	11.4				2		
Qin and Kaeriyama 2016		17	Western Subarctic Gyre	47.000	167.000		2009-00-00	Muscle			44.9	14.6	3.6	-19.8	*-16.1	n	1.0	-15.1	11.5				2		
Qin and Kaeriyama 2016		21	Western Subarctic Gyre	47.000	167.000		2009-00-00	Muscle			46.2	14.5	3.7	-19.8	*-16.1	n	1.0	-15.1	11.3				2		
Qin and Kaeriyama 2016		16	Western Subarctic Gyre	47.000	167.000		2009-00-00	Muscle			46.1	14.5	3.7	-19.7	*-16.1	n	1.0	-15.0	11.9				2		
Qin and Kaeriyama 2016		19	Western Subarctic Gyre	47.000	167.000		2009-00-00	Muscle			45.6	13.9	3.8	-19.6	*-15.9	n	1.0	-14.9	12.5				2		
Qin and Kaeriyama 2016		24	Western Subarctic Gyre	47.000	167.000		2009-00-00	Muscle			45.8	14.5	3.7	-19.6	*-15.9	n	1.0	-14.9	11.5				2		
Qin and Kaeriyama 2016		25	Western Subarctic Gyre	47.000	167.000		2009-00-00	Muscle						-19.4	*-15.7	n	1.0	-14.7	11.9				2		
Qin and Kaeriyama 2016		18	Western Subarctic Gyre	47.000	167.000		2009-00-00	Muscle			43.4	14.4	3.5	-19.2	*-15.5	n	1.0	-14.5	12.0				2		
Qin and Kaeriyama 2016		23	Western Subarctic Gyre	47.000	167.000		2009-00-00	Muscle			44.7	14.3	3.7	-19.2	*-15.5	n	1.0	-14.5	11.9				2		
Qin and Kaeriyama 2016		22	Western Subarctic Gyre	47.000	167.000		2009-00-00	Muscle			40.8	13.6	3.5	-19.0	*-15.3	n	1.0	-14.3	11.5				2		

Figure 1. Stable carbon and nitrogen isotope compositions of *O. nerka* and *O. kisutch*.

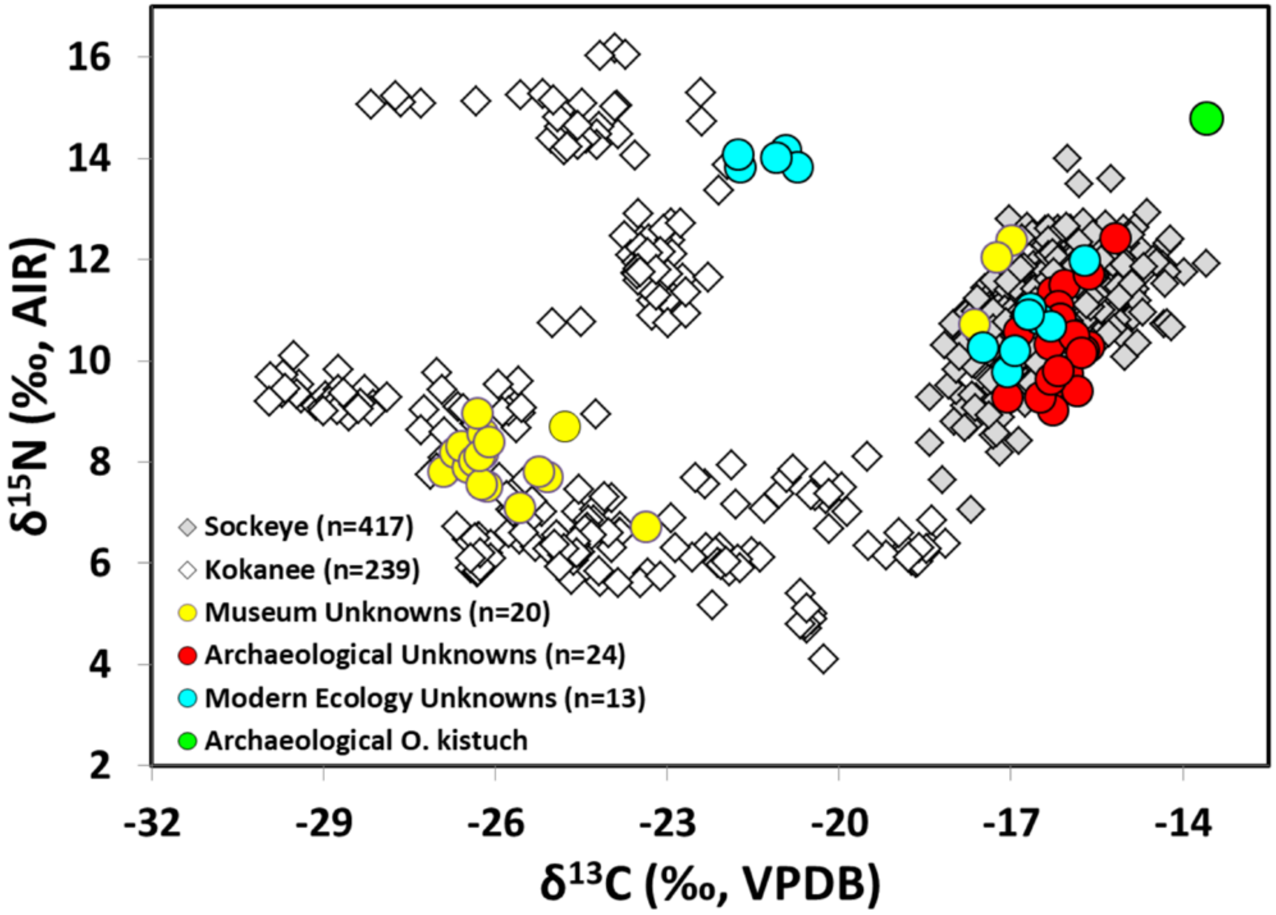

References

Guiry EJ, Szpak P, Richards MP. 2016. Effects of lipid extraction and ultrafiltration on stable carbon and nitrogen isotopic compositions of fish bone collagen. Rapid Communications in Mass Spectrometry 30(13):1591-1600.

Horii S, Takahashi K, Furuya K. 2015. Effects of ethanol-preservation on stable carbon and nitrogen isotopic signatures in marine predators. Plankton and Benthos Research 10(2):91-97.

Kaeriyama M, Nakamura M, Edpalina R, Bower J, Yamaguchi H, Walker R, Myers KW. 2004. Change in feeding ecology and trophic dynamics of Pacific salmon (Oncorhynchus spp.) in the central Gulf of Alaska in relation to climate events. Fisheries Oceanography 13(3):197-207.

Meeuwig MH, Peacock MM. 2017. Food Web Interactions Associated With a Lahontan Cutthroat Trout Reintroduction Effort in an Alpine Lake. Journal of Fish and Wildlife Management 8(2):449-464.

Molkentin J, Lehmann I, Ostermeyer U, Rehbein H. 2015. Traceability of organic fish–Authenticating the production origin of salmonids by chemical and isotopic analyses. Food Control 53:55-66.

Overman NC, Parrish DL. 2001. Stable isotope composition of walleye: 15N accumulation with age and area-specific differences in δ13C. Canadian Journal of Fisheries and Aquatic Sciences 58(6):1253-1260.

Qi H, Coplen TB, Geilmann H, Brand WA, Böhlke JK. 2003. Two new organic reference materials for $\delta^{13}C$ and $\delta^{15}N$ measurements and a new value for the $\delta^{13}C$ of NBS 22 oil. Rapid Communications in Mass Spectrometry 17(22):2483-2487.

Qi H, Coplen TB, Mroczkowski SJ, Brand WA, Brandes L, Geilmann H, Schimmelmann A. 2016. A new organic reference material, l-glutamic acid, USGS41a, for $\delta^{13}C$ and $\delta^{15}N$ measurements − a replacement for USGS41. Rapid Communications in Mass Spectrometry 30(7):859-866.

Qin Y, Kaeriyama M. 2016. Feeding habits and trophic levels of Pacific salmon (Oncorhynchus spp.) in the North Pacific Ocean. North Pacific Anadromous Fish Commission Bulletin 6:469-481.

Satterfield FR, Finney BP. 2002. Stable isotope analysis of Pacific salmon: insight into trophic status and oceanographic conditions over the last 30 years. Progress in oceanography 53(2):231-246.

Szpak P, Metcalfe JZ, Macdonald RA. 2017. Best practices for calibrating and reporting stable isotope measurements in archaeology. Journal of Archaeological Science: Reports 13:609-616.

Wang YV, Wan AH, Lock E-J, Andersen N, Winter-Schuh C, Larsen T. 2018. Know your fish: A novel compound-specific isotope approach for tracing wild and farmed salmon. Food chemistry 256:380-389.

Welch DW, Parsons TR. 1993. δ 13C - δ 15N values as indicators of trophic position and competitive overlap for Pacific salmon (Oncorhynchus spp.). Fisheries Oceanography 2(1):11-23.
