# Supplementary material for: Differentiating salmonid migratory ecotypes through stable isotope analysis of collagen: Archaeological and ecological applications
Source: PLoS One. 2020 Apr 28;15(4):e0232180. doi: 10.1371/journal.pone.0232180 (PMC7188214; doi:10.1371/journal.pone.0232180)
Supplement: S2 Text — (PDF) [file pone.0232180.s002.pdf]

S2 Text – Ancient DNA Analysis

Methods

Decontamination and DNA Extraction

To confirm their species identity and assign them to a sex, ancient DNA (aDNA) analysis was conducted on ten of the archaeological *Oncorhynchus* vertebrae included in our isotopic analysis. Sample decontamination, DNA extraction, and PCR setup procedures were conducted at the Department of Archaeology, Simon Fraser University (Burnaby, BC, Canada), in a dedicated aDNA laboratory and followed strict contamination control protocols (Yang and Watt 2005). All of the analyzed samples were decontaminated prior to DNA extraction using the protocol described by Speller and colleagues (2012). For decontamination, each sample was submerged in bleach (≈5% w/v NaOCl) for 7 min, rinsed twice in distilled water for 1–2 min and 10 min, and then UV irradiated in a crosslinker for 15–30 mins on two sides. DNA was extracted from the decontaminated samples using a modified silica-spin column method (Yang, et al. 1998; Yang, et al. 2008). The decontaminated samples were incubated overnight at 50 °C in 3.5 mL of lysis buffer (0.5 M EDTA pH 8.0, 0.25% SDS, and 0.5 mg/mL proteinase K) in a rotating hybridization oven. Following incubation, the samples were centrifuged, and 2.5 mL of the resulting supernatant was concentrated to ≤100 µL with an Amicon Ultra 10 kDA MWCO centrifugal filter (Millipore, Billerica, MA, USA). The concentrated extracts were then purified using a QIAquick PCR Purification Kit (Qiagen, Valencia, CA, USA). DNA extractions were performed in two batches that each included five samples and a blank extraction control.

Sex Identification

Sex identifications were assigned to the samples using the combined species and sex identification method described by Royle and colleagues (2018). In this method, sex identities are assigned to samples through two PCR-based sex identification assays (termed *clock1a*/*sdY* and D-loop/*sdY)* that co-amplify a 95 bp fragment of the male-specific Y-linked *Oncorhynchus* master sex-determining gene (sexually dimorphic on Y-chromosome gene [*sdY*]) and an internal positive control (IPC) (2018). Depending on the assay, this IPC consists of a 108 bp fragment of the nuclear *clock1a* (*clk1a*) gene amplified with primers *Clk1a*-F50 and *Clk1a*-R60 (*clock1a*/sdY assay) or a 249 bp fragment of the mitochondrial D-loop region amplified with primers Smc7 and Smc8 (D-loop/*sdY* assay) (Table 1). In both assays, *sdY* is amplified with primers *sdY*-F19 and *sdY*-R20 (Table 1).

Table 1. Primers used in this study.

Primer	Locus	Sequences (5'–3')	Amplicon Size	Reference
Clk1a-F50 (F)[1]	*clock1a*	TAGCCATGTCTGTGTGTTTACTTGC	108 bp	Royle, et al. 2018
Clk1a-R60 (R)	*clock1a*	GCAGCCAGCTAATTKGATTTG		Royle, et al. 2018
CytB5 (F)	*cytochrome b*	AAAATCGCTAATGACGCACTAGTCGA	168 bp	Yang, et al. 2004
CytB6 (R)	*cytochrome b*	GCAGACAGAGGAAAAAGCTGTTGA		Yang, et al. 2004
Smc7 (F)	D-loop	AACCCCTAAACCAGGAAGTCTCAA	249 bp	Yang, et al. 2004
Smc8 (R)	D-loop	CGTCTTAACAGCTTCAGTGTTATGCT		Yang, et al. 2004
sdY-F19 (F)	*sdY*	CCCAACACCCTTCCTATCTCC	95 bp	Royle, et al. 2018
sdY-R20 (R)	*sdY*	CCTTCCTCCCTAGAGCTTAAAAC		Royle, et al. 2018

[1]F denotes a forward primer and R indicates a reverse primer.

PCR amplifications were performed in a Mastercycler Gradient (Eppendorf, Mississauga, ON) thermocycler in a 30 µL reaction containing 1.5× PCR Gold Buffer (Applied Biosystems, Carlsbad, CA, USA), 2 mM $MgCl_2$, 0.2 mM of each dNTP, 0.45 µM of primers *sdY*-F19 and *sdY*-R20 (*clock1a*/*sdY* assay) or 0.6 µM of primers *sdY*-F19 and *sdY*-R20 (D-loop/*sdY* assay), 0.3 µM of primers *Clk1a*-F50 and *Clk1a*-R60 (*clock1a*/*sdY* assay) or 0.6 µM of primers Smc7 and Smc8 (D-loop/*sdY* assay), 1 mg/mL BSA, 3 µL DNA solution, and 1 U AmpliTaq Gold (Applied Biosystems, Carlsbad, CA). The thermocycling program for both PCR sex identification assays consisted of an initial denaturation step at 95 °C for 12 min followed by 60 cycles at 95 °C for 30 s (denaturation), 54 °C for 30 s (annealing), and 70 °C for 40 s (extension), and a final extension step at 72 °C for 7 min. To detect instances of contamination, a negative PCR control was included in each PCR run and each assay was applied to the blank extraction controls. All PCR and post-PCR procedures were conducted in a laboratory physically separated from the aDNA laboratory.

Following amplification, 5 µl of PCR product from each sample was pre-stained with SYBR Green I (Life Technologies, Carlsbad, CA), electrophoresed on a 3% agarose gel, and visualized with a Dark Reader transilluminator (Clare Chemical Research, Dolores, CO). Sex identities were assigned to the samples through a visual analysis of the electrophoresis gels using the criteria established by Royle and colleagues (2018). In brief, a sample was identified as male if *sdY* was successfully amplified with both assays, while a female identity was assigned to a sample if *sdY* was not amplified with either assay but both IPCs were amplified. No sex identity was assigned to a sample if the assays yielded discordant results or if one of the assays failed to amplify DNA.

Species Identification

Following Royle and colleagues (2018), we sought to assign species identifications to the samples by sequencing the D-loop fragment co-amplified as an IPC in the D-loop/*sdY* sex identification assay. To confirm the species identifications assigned to the samples, we also sequenced a 168 bp fragment of *cytochrome b*, which was amplified in a singleplex PCR with primers CytB5 and CytB6 (Table 1) (Royle, et al. 2020; Yang, et al. 2004). To improve sequencing quality, D-loop was also amplified from a single sample (IUBC 5226) through a singleplex PCR with the same primers (Smc7 and Smc8) used in the D-loop/*sdY* sex identification assay.

Singleplex PCR amplifications were performed in a Mastercycler Gradient or Personal thermocycler (Eppendorf, Mississauga, ON) in a 30 μL reaction volume that included 1.5× PCR Gold Buffer (Applied Biosystems, Carlsbad, CA), 2 mM $MgCl_2$, 0.2 mM of each dNTP, 0.3 μM of each *cytochrome b* or D-loop primer, 1 mg/mL BSA, 1.5–3 μL DNA solution, and 0.75–1 U AmpliTaq Gold (Applied Biosystems, Carlsbad, CA). The thermocycling program for the singleplex PCRs consisted of an initial denaturation step at 95 °C for 12 min followed by 60 cycles at 95 °C for 30 s (denaturation), 54 °C for 30 s (annealing), and 70 °C for 40 s (extension), and a final extension step at 72 °C for 7 min. Following amplification, 5 μl of PCR product from each sample was pre-stained with SYBR Green I (Life Technologies, Carlsbad, CA), electrophoresed on a 2–3% agarose gel, and visualized with a Dark Reader transilluminator (Clare Chemical Research, Dolores, CO). A negative PCR control was included in each singleplex PCR run in order to monitor for contamination. Singleplex PCRs were also performed on both blank extraction controls.

Successfully amplified D-loop and *cytochrome b* fragments were directly sequenced with the forward or reverse amplification primers at Eurofins Genomics (Toronto, ON). Prior to sequencing, the PCR products obtained from some of the samples were purified with ExoSAP-IT Express (Life Technologies, Carlsbad, CA) following the manufacturer's instructions. The obtained sequences were visually edited, truncated to remove the primer sequences, and compiled in ChromasPro v 2.1.8 (http://technelysium.com.au). To determine their closest taxonomic match, the edited sequences were compared against reference sequences accessioned in GenBank (Sayers, et al. 2019) through a BLASTn search (Altschul, et al. 1990). Multiple alignments of the ancient *cytochrome b* and D-loop sequences and reference sequences from 8 *Oncorhynchus* species (*O. clarkii*, *O. gorbuscha*, *O. keta*, *O. kisutch*, *O. masou*, *O. mykiss*, *O. nerka*, and *O. tshawytscha*) as well as *Salmo salar* were performed with Clustal W (Thompson, et al. 1994) through BioEdit v 7.2.5 (Hall, 1999). The resulting alignment was visually examined in BioEdit and the sequences were trimmed to the same length. For each marker, neighbour-joining trees were constructed in MEGA X (Kumar, et al. 2018) using a Kimura 2-parameter substitution model and 1000 bootstrap replications. Species-level identifications were assigned to samples if the obtained *cytochrome b* and D-loop sequences matched or closely resembled sequences from a single species and differed from closely related species (Yang, et al. 2004)

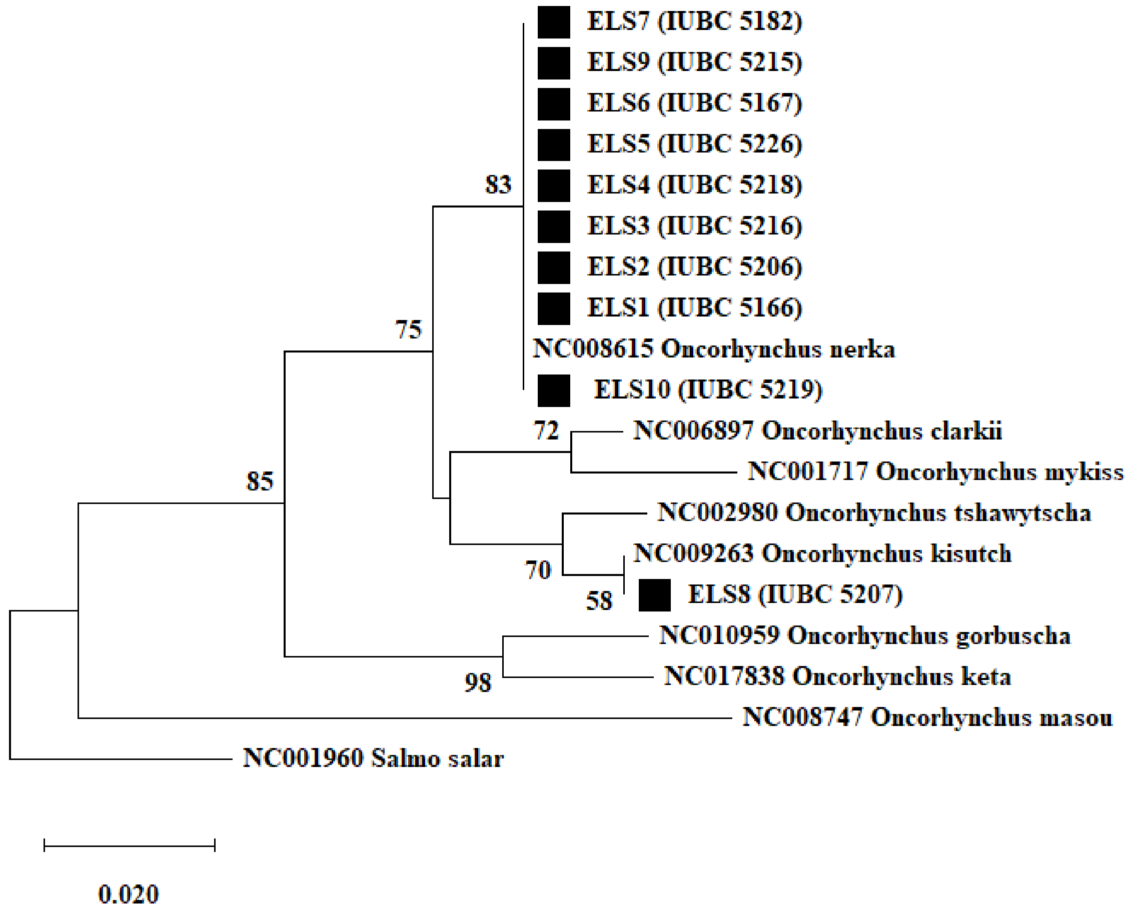

Fig 1. Neighbour-joining tree displaying the phylogenetic relationship between the *cytochrome b* sequences obtained from the archaeological salmonid samples analyzed in this study (denoted with filled squares; aDNA [ELS#] and isotope [IUBC #] lab numbers provided) and *Oncorhynchus* reference sequences (GenBank accession numbers shown). The tree was rooted using an Atlantic salmon (*Salmo salar*) sequence as an outgroup. The numbers at nodes denote the bootstrap values for nodes with ≥50% support after 1000 replications. The scale bar represents the number of nucleotide substitutions per site.

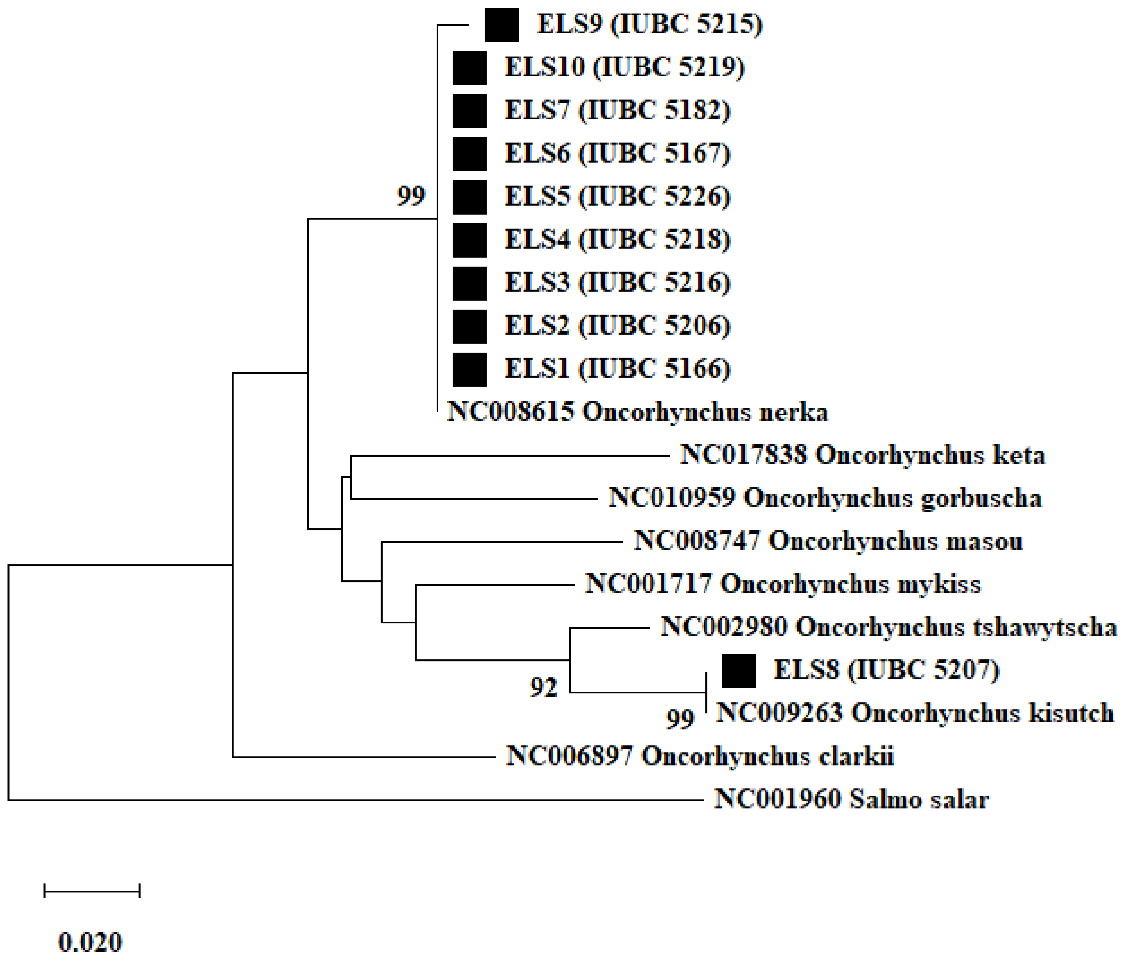

Fig 2. Neighbour-joining tree displaying the phylogenetic relationship between the D-loop sequences obtained from the archaeological salmonid samples analyzed in this study (denoted with filled squares; aDNA [ELS#] and isotope [IUBC #] lab numbers provided) and *Oncorhynchus* reference sequences (GenBank accession numbers shown). The tree was rooted using an Atlantic salmon (*Salmo salar*) sequence as an outgroup. The numbers at nodes denote the bootstrap values for nodes with ≥50% support after 1000 replications. The scale bar represents the number of nucleotide substitutions per site

References

Altschul SF, Gish W, Miller W, Myers EW, Lipman DJ. 1990. Basic local alignment search tool. Journal of Molecular Biology 215(3):403-410.

Sayers EW, Cavanaugh M, Clark K, Ostell J, Pruitt KD, Karsch-Mizrachi I. 2019. GenBank. *Nucleic Acids Research* 47(D1):D94-D99.

Hall TA. 1999. BioEdit: A user-friendly biological sequence alignment editor and analysis program for Windows 95/98/NT. Nucleic Acids Symposium Series 41:95-98.

Kumar S, Stecher G., Li M., Knyaz C., Tamura K. 2018. MEGA X: Molecular evolutionary genetics analysis across computing platforms. Molecular Biology and Evolution 35(6):1547-1549.

Royle TCA, Sakhrani D, Speller CF, Butler VL, Devlin RH, Cannon A, Yang DY. 2018. An efficient and reliable DNA-based sex identification method for archaeological Pacific salmonid (*Oncorhynchus* spp.) remains. PloS ONE 13(3):e0193212.

Royle TCA, Zhang H, Guiry EJ, Orchard TJ, Needs-Howarth S, Yang DY. 2020. Investigating the sex selectivity of a Middle Ontario Iroquoian Atlantic salmon (*Salmo salar*) and lake trout (*Salvelinus namaycush*) fishery through ancient DNA analysis. Journal of Archaeological Science: Reports: Forthcoming.

Speller CF, Hauser L, Lepofsky D, Moore J, Rodrigues AT, Moss ML, McKechnie I, Yang DY. 2012. High potential for using DNA from ancient herring bones to inform modern fisheries management and conservation. PloS ONE 7(11):e51122.

Thompson JD, Higgins DG, Gibson TJ. 1994. CLUSTAL W: Improving the sensitivity of progressive multiple sequence alignment through sequence weighting, position-specific gap penalties and weight matrix choice. Nucleic Acids Research 22(22):4673-4680.

Yang DY, Cannon A, Saunders SR. 2004. DNA species identification of archaeological salmon bone from the Pacific Northwest Coast of North America. Journal of Archaeological Science 31(5):619-631.

Yang DY, Eng B, Waye JS, Dudar JC, Saunders SR. 1998. Improved DNA extraction from ancient bones using silica-based spin columns. American Journal of Physical Anthropology 105(4), 539-543.

Yang DY, Liu L, Chen X, Speller CF. 2008. Wild or domesticated: DNA analysis of ancient water buffalo remains from north China. Journal of Archaeological Science 35(10):2778-2785.

Yang DY, Watt K. 2005. Contamination controls when preparing archaeological remains for ancient DNA analysis. Journal of Archaeological Science 32(3), 331-336.
